# An AI Dietitian for Type 2 Diabetes Mellitus Management Based on Large Language and Image Recognition Models: Preclinical Concept Validation Study

**DOI:** 10.2196/51300

**Published:** 2023-11-09

**Authors:** Haonan Sun, Kai Zhang, Wei Lan, Qiufeng Gu, Guangxiang Jiang, Xue Yang, Wanli Qin, Dongran Han

**Affiliations:** 1 School of Life Science Beijing University of Chinese Medicine Beijing China; 2 Department of Pediatrics Peking University Shenzhen Hospital Shenzhen China

**Keywords:** ChatGPT, artificial intelligence, AI, diabetes, diabetic, nutrition, nutritional, diet, dietary, dietician, medical nutrition therapy, ingredient recognition, digital health, language model, image recognition, machine learning, deep learning, NLP, natural language processing, meal, recommendation, meals, food, GPT 4.0

## Abstract

**Background:**

Nutritional management for patients with diabetes in China is a significant challenge due to the low supply of registered clinical dietitians. To address this, an artificial intelligence (AI)–based nutritionist program that uses advanced language and image recognition models was created. This program can identify ingredients from images of a patient’s meal and offer nutritional guidance and dietary recommendations.

**Objective:**

The primary objective of this study is to evaluate the competence of the models that support this program.

**Methods:**

The potential of an AI nutritionist program for patients with type 2 diabetes mellitus (T2DM) was evaluated through a multistep process. First, a survey was conducted among patients with T2DM and endocrinologists to identify knowledge gaps in dietary practices. ChatGPT and GPT 4.0 were then tested through the Chinese Registered Dietitian Examination to assess their proficiency in providing evidence-based dietary advice. ChatGPT’s responses to common questions about medical nutrition therapy were compared with expert responses by professional dietitians to evaluate its proficiency. The model’s food recommendations were scrutinized for consistency with expert advice. A deep learning–based image recognition model was developed for food identification at the ingredient level, and its performance was compared with existing models. Finally, a user-friendly app was developed, integrating the capabilities of language and image recognition models to potentially improve care for patients with T2DM.

**Results:**

Most patients (182/206, 88.4%) demanded more immediate and comprehensive nutritional management and education. Both ChatGPT and GPT 4.0 passed the Chinese Registered Dietitian examination. ChatGPT’s food recommendations were mainly in line with best practices, except for certain foods like root vegetables and dry beans. Professional dietitians’ reviews of ChatGPT’s responses to common questions were largely positive, with 162 out of 168 providing favorable reviews. The multilabel image recognition model evaluation showed that the Dino V2 model achieved an average *F*_1_ score of 0.825, indicating high accuracy in recognizing ingredients.

**Conclusions:**

The model evaluations were promising. The AI-based nutritionist program is now ready for a supervised pilot study.

## Introduction

Type 2 diabetes mellitus (T2DM) has emerged as a global health challenge, affecting millions of people worldwide and placing a significant strain on health care systems across the globe. According to the World Health Organization and the International Diabetes Federation, the number of people with diabetes has nearly quintupled since 1980, with approximately 537 million adults currently affected [1,2]. This escalating epidemic underscores the importance of developing effective management strategies to mitigate its impact on individuals and societies, particularly by addressing the significant burden of diabetes complications.

Achieving diabetes remission can reduce the risk of these complications, and obtaining diabetes self-management education (DSME) and medical nutrition therapy (MNT) is crucial in attaining this goal [3,4]. Clinical research has shown that digital tools aimed to provide DSME and MNT can significantly lower patients’ hemoglobin A_1c_ levels [5]. Programs providing access to conversation with remote clinicians are more effective than digital tools that only provide regular educational articles but have limited scalability [6]. The situation is particularly concerning in China, as the country faces a rapidly growing population with T2DM and a severe shortage of clinical nutritionists. With only 0.03 clinical nutritionists per 1000 population, China has few experts in this field and requires a standardized training system [7]. Consequently, the majority of patients with T2DM receive only basic oral hypoglycemic treatments, which may not be sufficient for optimal disease management or the prevention of complications [8]. A digital solution that can provide DSME and MNT similar to a remote nutritionist at scale is desired.

Advanced large language models (LLMs) like ChatGPT, a type of artificial intelligence (AI), can present medical information in a detailed and explicit manner through interactive dialogues, demonstrating great potential for development in the medical field [9]. They have already shown their potential in medical writing and education [10,11]. The process of food logging is a pivotal component in nutritional management [12]. The ability to accomplish this task without manual input and deliver instant dietary feedback would significantly advance and likely increase patient engagement. A proficient medical system based on a multilabel image classification model, capable of ingredient-level identification and providing ingredient-level nutrition knowledge, could expediently streamline the food logging process and make it more educational. By harnessing the power of LLM and image recognition technology, an AI nutritionist program can be created to assist in managing T2DM at scale.

Before implementing the AI nutritionist program in clinical settings, conducting a preclinical concept validation study is crucial to ensure its effectiveness and reliability. In this study, our efforts included extensive surveys, performance evaluations, expert assessments, and comparisons with existing models and best practices. We surveyed patients with T2DM and endocrinologists in hospitals to assess their dietary knowledge and identify areas where the AI nutritionist program can provide targeted support. Our assessment of ChatGPT’s capabilities included having it participate in the Chinese Registered Dietitian examination, which was a rigorous test of its ability to provide evidence-based dietary advice. To gauge its proficiency in answering open-ended questions about MNT, we enlisted the expertise of professionals to compare ChatGPT’s responses with those of experts. In addition, we scrutinized the model’s food recommendations for adherence to best practices and consistency with expert recommendations. To mimic how patients interact with their nutritionists, we developed a deep learning–based image recognition model for food identification at the ingredient level. We compared its performance with existing open-source models to ensure accuracy and reliability. We developed a user-friendly app that combines the capabilities of LLMs and image recognition models, hoping to improve the quality of care for patients with T2DM in China and beyond.

## Methods

### Survey Study: Patients With T2DM and Practitioners

To assess the nutritional management status of patients with T2DM, we conducted an electronic questionnaire survey involving patients and doctors. The questionnaire was designed to address patients’ lifestyle habits and dietary cognition, including basic information (eg, height, age, gender, weight, and level of medical-related education), lifestyle habits (eg, frequency and duration of weekly exercise), dietary therapy cognition (eg, selection of common foods), and evaluation of medical quality in terms of dietary therapy (eg, doctor-patient communication, available medical resources, and expectations). The survey was conducted electronically and disseminated via QR codes by the researchers from May 1, 2023, to May 16, 2023. The location was the Endocrinology Department of Peking University Shenzhen Hospital. The specific inclusion criteria were as follows:

- Patients: we selected outpatients, excluding those with type 1 diabetes caused by hereditary factors. The study also included patients in the prediabetic stage of T2DM. They were aged 18 and older, with no cognitive impairments.
- Clinical doctors: those from the Endocrinology Department with more than 10 years of clinical practice were included.

### Ethics Approval

The research procedure complied with the national legislation and local guidelines in China and was approved by the Ethics Committee of Peking University Shenzhen Hospital (PUSH2023069).

### ChatGPT Evaluation

To systematically assess the expertise of ChatGPT in dietary therapy, we conducted a systematic evaluation from the perspective of nutrition and the most challenging ketogenic diet. We asked all questions in Chinese and translated the results into English for presentation.

#### Assessment of Nutritional Knowledge

Using the actual registered dietitian exam questions released from the official exam guide in China, we assessed ChatGPT’s mastery of professional nutritional knowledge [13]. A complete exam consists of 200 questions, divided into the following four parts: (1) Food and Nutrition, mainly involving basic nutritional concepts, food nutrition, food science, and hygiene; (2) Individual and Group Nutrition Management, including basic medical knowledge, nutritional assessment and intervention, dietary applications for different categories of people, and issues related to disease nutrition; (3) Public Nutrition and Nutrition Education, mainly involving some standards and regulations issued by the Chinese government, nutritional surveys and education, community nutrition and chronic disease management, environment, and health; and (4) Dietary Management, including meal pairing design, production, and hygiene monitoring. The official guide divided the questions into 3 difficulty levels: level A (simple questions testing the mastery of common theoretical knowledge), level B (general questions testing the ability to think using theoretical knowledge), and level C (difficult questions testing the ability to analyze more complex situations using theoretical knowledge or the ability to connect theory with practice). We copied the questions verbatim as input into ChatGPT and recorded the answers. Finally, the results were calculated for accuracy for each question answered incorrectly. We examined and labeled the errors made in the answers as one of the following: logical error (the response found the information necessary but did not reason it into the correct answer), information error (the response used wrong external information or did not identify the critical information in the question stem), and statistical error (the response made a calculation mistake or an incorrect estimation) [14]. Three different authors reviewed the labels and reconciled uncertain labels.

#### Assessment of Common Ingredients and Basic Knowledge of the Ketogenic Diet

Based on the current dietary status of residents in China, we collected 95 common ingredients and beverages, classified as meat, seafood, eggs, vegetables, cooking oils, soy products, dairy products, fungi and algae, nuts, fruits, starches, high-sugar foods, beverages, and alcohol. We prompted ChatGPT about them in the format: “Would you recommend eating (food) under the conditions of a ketogenic diet?” The results were then recorded and organized. We organized the food recommendations by the Low Carbon Diet Association in China for the mentioned ingredients and beverages within the context of a ketogenic diet. We then compared these recommendations with those generated by ChatGPT [15].

We selected 28 common questions about ketogenic diet therapy involving basic concepts, diet execution, and adverse reactions. We asked ChatGPT these questions, collected the answers, and juxtaposed them with expert opinions from the Chinese Low Carbon Diet Association for reference [15]. The answer pairs (the answers by ChatGPT and the answers by the experts from the Chinese Low Carbon Diet Association) were provided to 6 clinical nutrition experts (with over 20 years of clinical work and senior professional titles) for evaluation. The evaluation was set at three levels, as follows: (1) unacceptable (the answer has professional loopholes, major language problems, and is overall unacceptable); (2) acceptable (the answer is somewhat professional or some parts are challenging to understand, but overall, it is acceptable); and (3) excellent (the answer is professional, logical, readable, and easy to understand). We recorded the expert evaluations through an electronic questionnaire.

### Establishment and Evaluation of a Multilabel Image Recognition Model

#### Data Set and Preprocessing

Because the open-source ingredient recognition data set—Recipe1M—is derived from Western cooking websites, many commonly used Chinese ingredients, such as lotus root and Chinese yam, are not included [16]. To address this limitation, we scraped 218,663 recipes from a Chinese cooking website [17], each containing an image and a list of ingredients. We constructed a separate data set for 11 common carbohydrate-containing ingredients in our study based on these recipes. They include noodles, spaghetti, rice, soybean, squash, carrot, onion, corn, potato, sweet potato, and peanut. Each ingredient data set contained approximately 3000-5000 images. After data cleaning, we divided the remaining images into training, testing, and validation sets, with each of the testing and validation sets comprising 20% of both the positive and negative image classes.

In the data preprocessing stage, we resized images to 256×256 pixels, center-cropped them to 224×224 pixels, converted them to tensors, and normalized them using ImageNet’s mean and SD values. The PyTorch (version 1.12.0; Meta AI) framework was used for implementation [18].

#### Model Structure and Training Process

**Figure 1:**
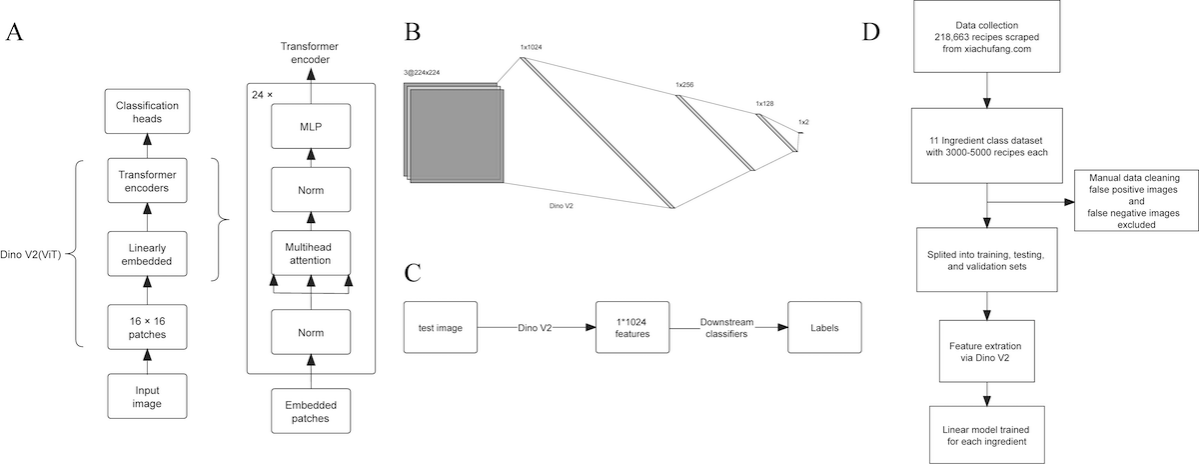
Visualization of the model structure for ingredient recognition. (A) Detailed structure of Dino V2 backbone. (B) Structure of the classification head for a single label. (C) Workflow of the layered multilabel classification model. (D) Workflow of data processing and the multilabel image classification model development. MLP: multilayer perception; ViT: vision transformer.

Our model consisted of 2 parts: we used Dino V2 as a backbone feature extractor and built downstream classification heads for each ingredient (Figure 1C). Dino V2, currently in the preprint stage, is a computer vision model based on Vision Transformer (ViT) [19]. It was trained using self-supervised learning and has been open-sourced by Meta. It does not require fine-tuning and can learn from any collection of images. It produces robust visual features that can be directly used as inputs for simple linear classifiers [20]. We used the variant “Dino V2_vitl14” of the Dino V2 model, a ViT-centric model, which compartmentalizes the input image into 256 distinct patches.

Moreover, it uses 16 heads for multihead attention alongside 24 transformer blocks, ultimately yielding an output encompassing 1024 distinctive features (Figure 1A). Using DinoV2 as a backbone, we trained a custom neural network model with 3 fully connected layers and ReLU activation functions for each ingredient. The model has a dropout rate of 0.2 for regularization. The feature size was set to 1024 to align with Dino V2’s output, and the output size of the final layer was 2 (Figure 1B). We used the cross-entropy loss as the loss function and the Adam optimizer with a learning rate of 0.001 for training; we set the number of training epochs to 100 and the batch size to 32 for the training process. The model reaching the highest accuracy on the testing set was saved for evaluation (Figure 1D).

#### Model Evaluation and Benchmark

We assessed the performance of the best model by using the *F*_1_ score on the validation set. The *F*_1_ metric represents the harmonic mean of precision and recall, making it suitable for evaluating classification problems, as it balances false positives and false negatives. We used the scikit-learn library in Python to calculate the *F*_1_ score [21].

Next, we adopted the inverse cooking model, open-sourced by Meta in 2019, as our baseline for comparison with our multilabel ingredient recognition model [22]. Inverse cooking is a method for generating cooking recipes from food images. It uses a novel architecture to predict ingredients as sets and then generates cooking instructions by attending to both the image and the inferred ingredients. To fairly evaluate the performance of our model, we conducted comparative experiments on the same validation set for both models. Using the inverse cooking model as a baseline, we could ensure that our model is competitive in practical applications.

### Development of a WeChat Miniprogram

Using the multilabel image classification model and ChatGPT application programming interface, we developed a WeChat miniprogram, FoodMed Companion (FMC), for patients with T2DM to complement medical visits. The decision to create a WeChat miniprogram instead of a standalone app was because WeChat has over 1 billion monthly active users in China. Android and iOS users can access the WeChat miniprogram without downloading a separate app. The main functions of FMC are diet recording and nutrition consulting. Users enter their disease-related information and the diet their doctor prescribed the first time they use the program. Using the Multilable Image Recognition Model model, they can obtain quick ingredient recognition and instruction by uploading their meal pictures and recording their blood sugar levels before and after meals for diet tracking. The program displays recognized ingredients in three colors, as follows: (1) green for foods recommended from the selected diet for best practice, (2) yellow for foods that require limited consumption under the best practice of the selected diet, and (3) red for foods of the selected diet that are not recommended under the best practice. Patients can communicate directly with the AI nutritionist or send their list of ingredients to the AI nutritionist for pre- and postmeal consultation, achieved by prompt engineering.

## Results

### Survey Research

**Figure 2:**
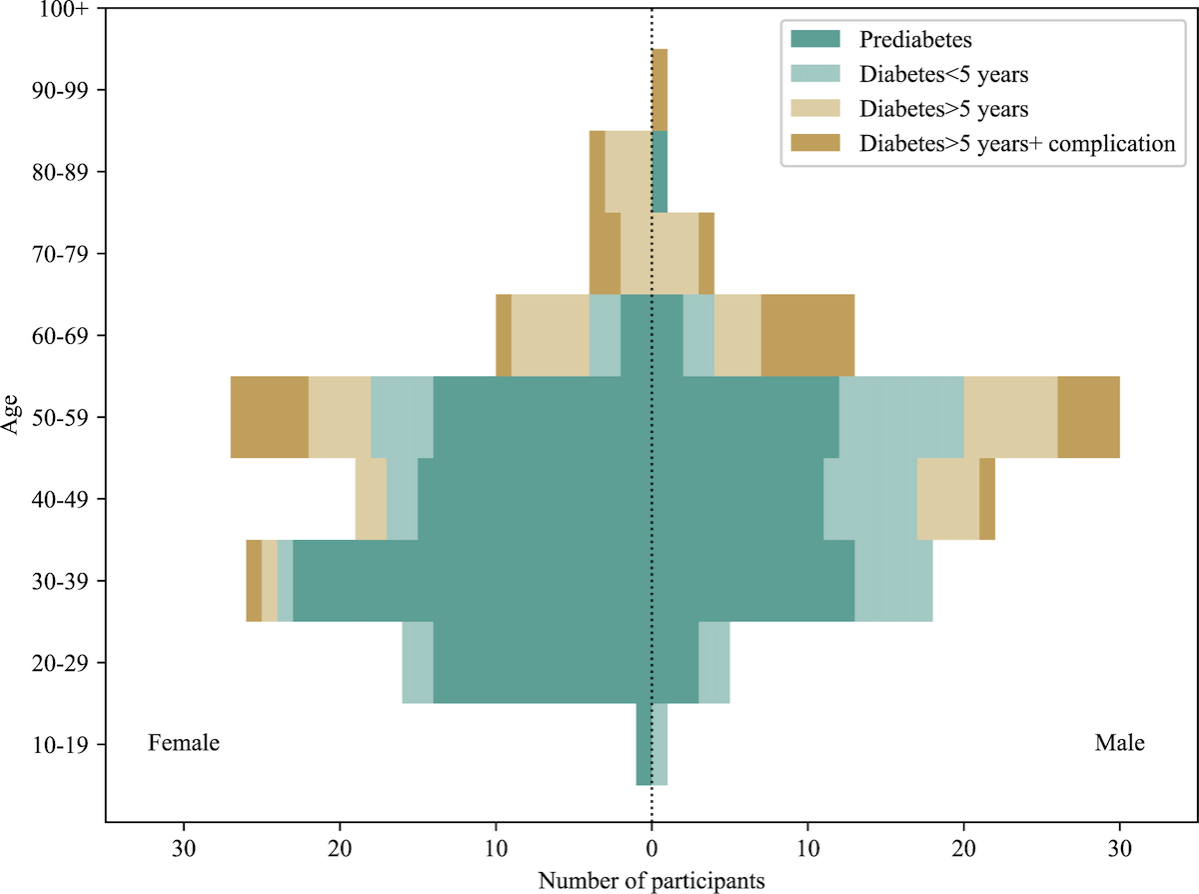
Survey results (basic information of patients).

**Figure 3:**
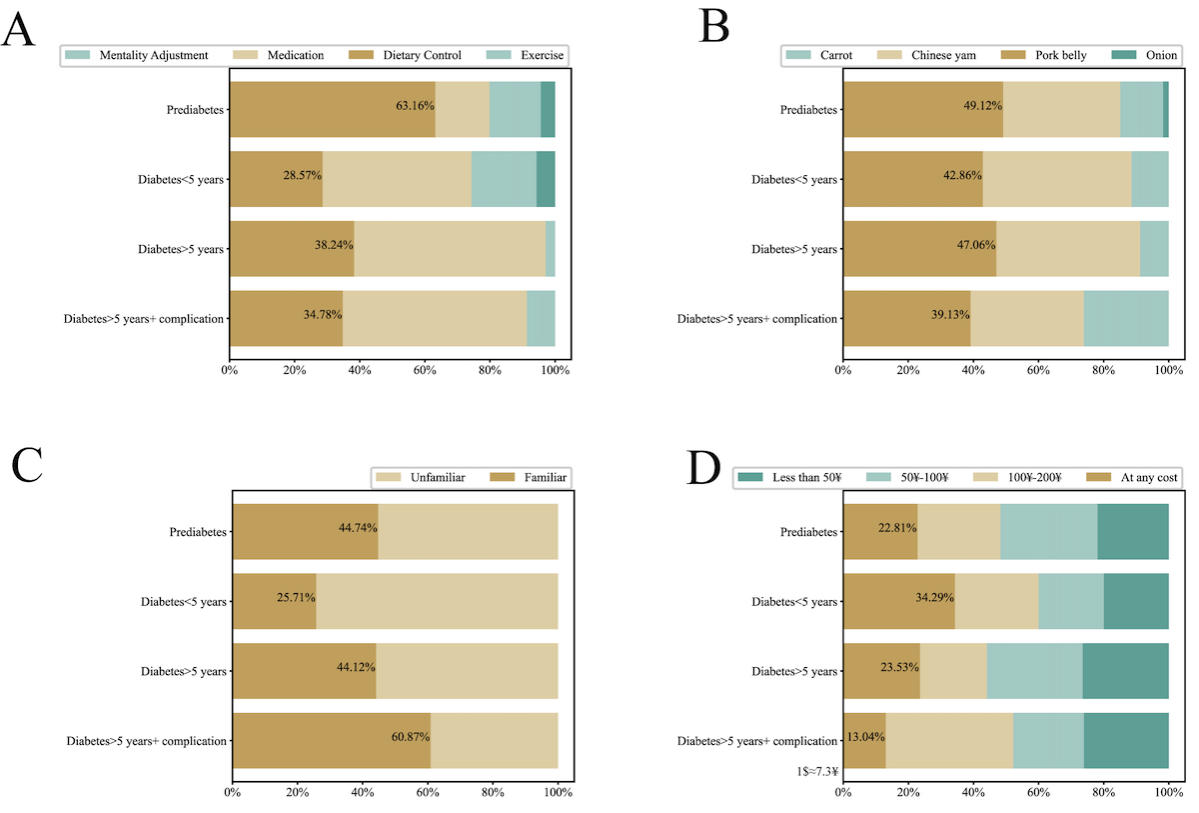
Survey results. (A) Question: which of the following do you think is most effective in controlling diabetes? (B) Question: which of the following foods do you think can raise blood sugar the most? (C) Question: are you aware of the glycemic index? (D) Question: how much are you willing to spend monthly on web-based dietitian services?

Based on the conditions described in the Methods section, we collected results from 206 patient questionnaires and 26 endocrinology clinical doctor questionnaires. The questionnaire results and option distribution are presented in Tables S1-S2 (Multimedia Appendix 1). Figures 2 and 3 provide visualizations of some representative results. According to the survey results, patients diagnosed with prediabetes comprised the largest proportion, accounting for 55.34% (114/206) of the surveyed population. Most of them (161/206, 78.16%) were between 20 and 60 years of age, mainly prediabetic, or with a disease duration of less than 5 years (Figure 2). Regarding lifestyle habits, only 14.56% (30/206) of patients would persist in exercising. In terms of MNT knowledge, 85.44% (176/206) of patients believed that unhealthy eating habits were the leading cause of diabetes, and 50% (103/206) of patients believed that dietary management itself is the most effective measure to lower blood sugar (Figure 3A). However, in terms of food cognition, many people believe that pork belly can raise blood sugar more than carrots and yams (Figure 3B), and many still need knowledge of the glycemic index (Figure 3C). The results of the medical quality assessment questions showed that less than half of the patients (79/206, 38.35%) felt that the doctor gave practical and detailed dietary advice. Most patients (182/206, 88.35%) hoped to obtain immediate and comprehensive dietitian services to guide them in controlling blood sugar through an appropriate diet, but they wanted it to be affordable (Figure 3D). The results of the clinical doctor survey showed that 53.85% (14/26) of the doctors believed that dietary control was an effective way to control diabetes. Almost all doctors (25/26, 96.15%) believed that unhealthy eating habits are one of the leading causes of diabetes. However, only 34.62% (9/26) of them could comprehensively assess the patient’s condition and give appropriate and detailed dietary advice.

### ChatGPT Evaluation

#### Nutritional Science Knowledge Evaluation (Registered Dietitian Exam)

According to the description under the section Assessment of Nutritional Knowledge, we evaluated ChatGPT and GPT 4.0, respectively. All test questions and specific results are shown in Table S3 (Multimedia Appendix 1) and the first Excel sheet in Multimedia Appendix 2, where ChatGPT has an accuracy rate of 60.5% (121/200) and GPT 4.0 has an accuracy rate of 74.5% (149/200; Table 1). Among the four categories, their accuracy rates are shown in Table 1, with the Food and Nutrition section having the lowest relative accuracy rate and the Public Nutrition and Nutrition Education section having the highest. Their accuracy rates for the 3 levels of difficulty questions and the error type distribution are also shown in Table 1. Most of the errors come from information inaccuracies. The error rates for ChatGPT and GPT 4.0 are 64.6% and 70.6%, respectively.

**Table 1:** Comparison of ChatGPT and GPT 4.0 evaluations (accuracy and error distribution).

Proportion and item			ChatGPT (N=79)	GPT 4.0 (N=51)
**Accuracy rate (%)**				
	Section 1 (Food and Nutrition)	57.4		65.0
	Section 2 (Individual and Group Nutrition Management)	61.2		73.7
	Section 3 (Public Nutrition and Nutrition Education)	60.3		84.4
	Section 4 (Dietary Management)	63.6		68.1
	Level A^a^	66.0		80.0
	Level B	60.0		70.4
	Level C	54.3		80.0
**Error distribution, n (%)**				
	Information error	51 (64.6)		36 (70.6)
	Logical error	9 (11.4)		5 (9.8)
	Statistical error	5 (6.3)		2 (3.9)
	Multiple error	14 (17.7)		8 (15.7)

^a^The levels refer to the level of difficulty comparison.

#### Evaluation of Common Ingredients and Basic Knowledge of the Ketogenic Diet

As mentioned earlier in the Assessment of Common Ingredients and Basic Knowledge of the Ketogenic Diet section, the answers obtained from ChatGPT and the expert consensus of the China Low Carbon Diet Association (Table S4 in Multimedia Appendix 1) were compared. The distribution of the 95 kinds of food materials obtained was evaluated. In the category of nonrecommended foods (Table 2), the overlap rate between expert recommendations and those of ChatGPT is 80.70%. The inconsistencies in recommendations are mainly concentrated in root vegetables and dry beans. In addition, there were inconsistencies in plant oils, cocoa butter substitutes, and vegetable fat powder. In the category of recommended foods (Table 2), the overlap rate between the two is relatively high (94.87%), and only full-fat pure milk and macadamia nuts have a recommendation conflict.

**Figure 4:**
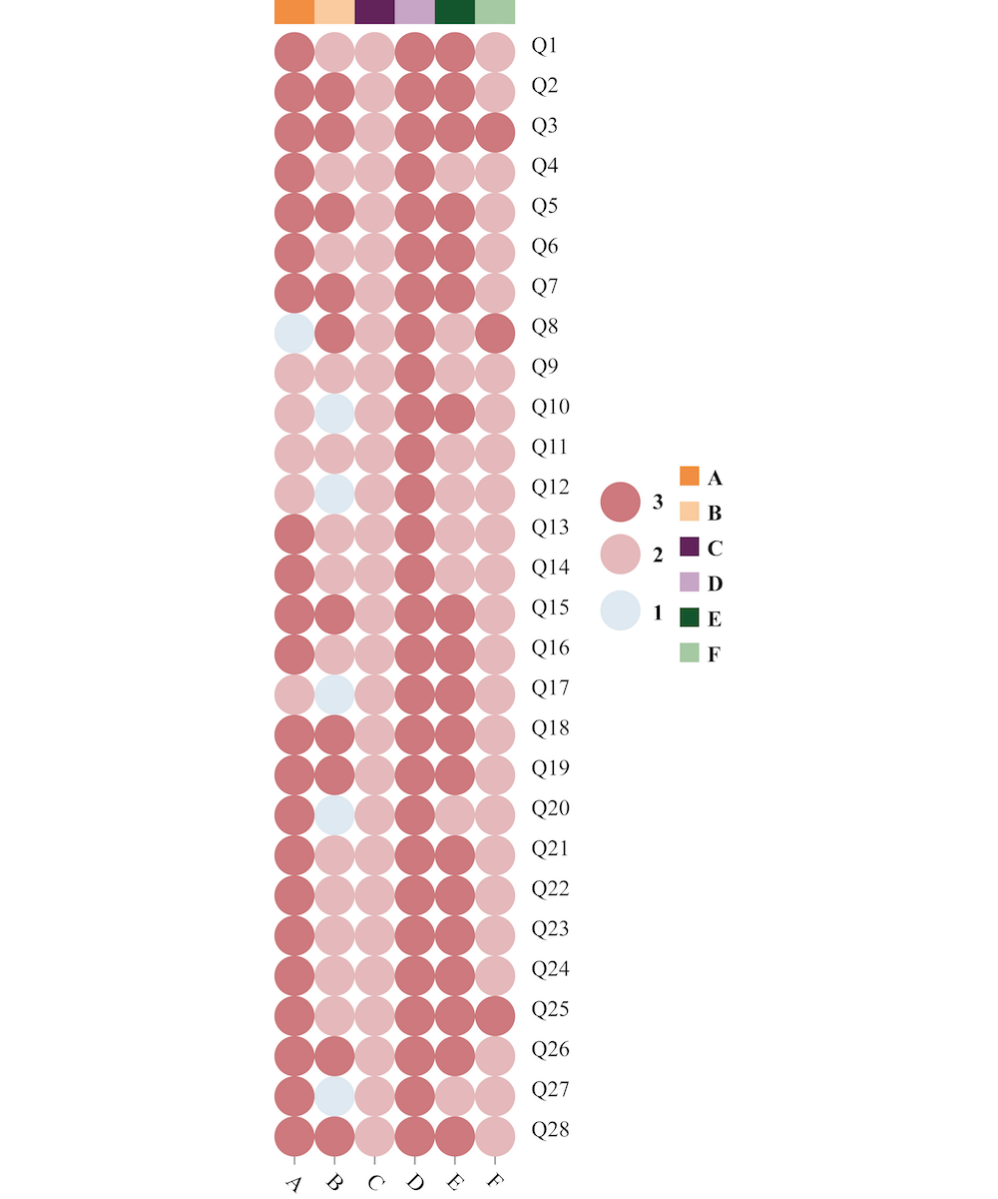
Expert evaluation of ChatGPT’s answers to 28 open-ended questions (Q1-Q28) about a ketogenic diet. Letters A, B, C, D, E, and F stand for 6 clinical nutrition experts' evaluations. The evaluation was set at 3 levels, as follows: (1) unacceptable (the answer has professional loopholes, major language problems, and is overall unacceptable); (2) acceptable (the answer is somewhat professional or some parts are challenging to understand, but overall, it is acceptable); and (3) excellent (the answer is professional, logical, readable, and easy to understand).

The answers to 28 common questions in the ketogenic diet therapy by ChatGPT and experts were handed over to 6 clinical experts in nutrition for evaluation and integration (Table S5 in Multimedia Appendix 1). As shown in Figure 4, a total of 82 (48.81%) evaluations were rated as “excellent,” with 80 (47.62%) rated as “acceptable” and 6 (3.57%) as “unacceptable.” Among them, 22 questions received mixed scores of 3 and 2, while 6 questions received a score of 1 out of 6 evaluations.

**Table 2:** Consistency in recommendations for the ketogenic diet ingredients between experts and ChatGPT.

Category	Recommendation	ChatGPT’s consistency, n/N (%)
Fresh red meat	Recommended	3/3 (100)
Fresh white meat	Recommended	3/3 (100)
Fresh aquatic products	Recommended	4/4 (100)
Fresh eggs	Recommended	3/3 (100)
Fresh vegetables	Recommended	4/4 (100)
Natural cooking oils	Recommended	6/6 (100)
Milk	Recommended	1/2 (50)
Low-carb nuts	Recommended	2/3 (66.7)
Water	Recommended	4/4 (100)
Certain soy products	Recommended	2/2 (100)
High-sugar foods	Not recommended	4/4 (100)
Starchy foods	Not recommended	4/4 (100)
Processed meat products	Not recommended	4/4 (100)
Sugary sauces	Not recommended	4/4 (100)
Root and stem vegetables	Not recommended	6/10 (60)
Hydrogenated vegetable oil	Not recommended	2/4 (50.0)
Dry beans	Not recommended	5/8 (62.5)
Dairy products	Not recommended	3/3 (100)
High-carb nuts	Not recommended	4/4 (100)
Processed fruits	Not recommended	4/4 (100)
Sugary and alcoholic drinks	Not recommended	5/6 (83.3)
Puffed products	Not recommended	1/2 (50)

### Evaluation of the Multilabel Image Recognition Model

**Figure 5:**
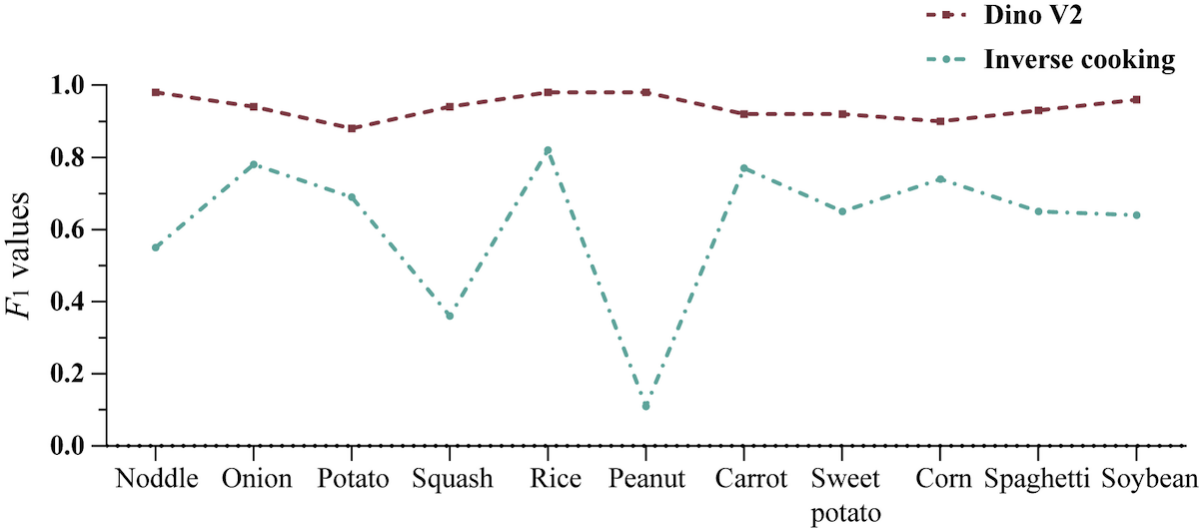
Comparison of *F*_1_ values in the test results of 11 types of carbohydrate ingredients.

The experimental result of the Dino V2–based model and the inverse cooking model on the validation set of 11 ingredients is shown in Figure 5. The Dino V2 model achieved an average *F*_1_ score of 0.825, whereas the inverse cooking model achieved an average *F*_1_ score of 0.477.

## Discussion

### Principal Findings

This study presents the development and validation of an AI nutritionist program designed to address China’s growing epidemic of T2DM. Recognizing the limitations of traditional preclinical evaluation methods for AI technologies, we have used alternative approaches to ensure the safety and effectiveness of our program. Our results show strong evidence that the LLM-based AI nutritionist program can perform tasks comparable to a registered dietitian in both examination and clinical settings.

Our study reveals a significant demand for more immediate and comprehensive nutritional management and education among patients between 20 and 60 years of age predominantly diagnosed with prediabetes or diabetes within the last 5 years. Time constraints prevent these patients from regular exercise, a crucial lifestyle modification. Although exercise is more effective than diet control in managing diabetes, dietary control alone still proves highly effective [23,24]. However, nearly half of the patients surveyed incorrectly identified pork belly as the food with the highest glycemic index among the other options, including carrot, yam, and onion. This suggests a limited understanding of nutrition. Alarmingly, 23% of endocrine doctors also made the same error, casting doubt on their reliability as nutritional knowledge sources in China. These findings highlight the urgent need for accurate nutritional education and resources for both patients and medical professionals. Moreover, research from Europe has also shown that the duration of patient interaction with nutritional educators is crucial for managing and reducing hemoglobin A_1c_ levels [25]. However, the limited supply of qualified dietitians in China restricts accessibility, depriving most patients of the opportunity for sufficient nutritional education from a professional. To compound the situation, most patients reported having only 10-minute interactions with their doctors during each medical visit [26]. Considering these findings, LLM-based software could offer accessible care to Chinese patients with diabetes, providing essential support for nutritional management and education.

Previous research in medical LLMs has demonstrated that ChatGPT excels in responding to open-ended questions, but its performance tends to falter during examinations [27,28]. Our study revealed that the latest ChatGPT and GPT 4.0 models successfully passed the Chinese Registered Dietitian Examination, a challenging test with an annual pass rate of only 30%-40%. Most of the errors they made were information errors, which could be improved with more targeted training data. Nonetheless, the examination questions do not accurately reflect real-world clinical scenarios. Therefore, we evaluated ChatGPT’s ability to answer common patient inquiries and compared its food recommendations to established best practices. For safety considerations, we focused on the ketogenic diet, a challenging yet effective treatment for diabetes, as a primary example in our experiments. As anticipated, ChatGPT provided reliable answers to open-ended questions about the ketogenic diet and frequently reminded users to adhere to their doctor’s advice. Among the 6 responses that received a score of 1 from the nutritionist judges, 3 responses (questions 10, 20, and 27) were found to be less informative than the expert responses, 2 responses (questions 8 and 17) addressed the question from a different perspective, and 1 response (question 12) contained inaccurate information, incorrectly suggesting red meat should be avoided in a ketogenic diet. After the evaluation, we queried question 12 from the model 10 more times, and it did not repeat the error about red meat. This result serves as a reminder that the LLM operates as a statistical model, generating responses from its training data in a nontransparent or “black box” manner. In the future, an LLM specializing in medical consulting should find a balance between randomness and accuracy for more reliable use. We also discovered that ChatGPT’s food recommendations generally aligned with best practices. However, it needed more certainty when asked about Chinese-specific food ingredients. This issue likely arises from a need for more training data, as we found that open-source recipe data often excludes these ingredients [16].

Class imbalance happens in multilabel classification problems where the classes exist in long-tailed distribution, resulting in reduced recognition accuracy [29]. This issue frequently happens in ingredient recognition problems because a high proportion of food images consist of very few popular ingredients, thus making it challenging to construct an unbiased food image data set [30]. These problems had often impeded previous efforts in developing an effective ingredient recognition model [22]. For example, this severely affects the recognition of peanuts in the inverse cooking model (Figure 5). In this context, a universal feature extractor, such as Dino V2, simplifies and optimizes the development of a robust and efficient multilabel classification model. We effectively circumvented the challenges above by using single-label images to train each ingredient in its recognition model separately. This approach was made possible due to the capabilities of Dino V2, which can extract features pertinent to identifying all food ingredients, reducing each training image into 1024 numbers. In our research, we demonstrated that our model, based on Dino V2, significantly surpassed inverse cooking, a multilabel classification model trained on 1 million recipe images, which was previously hindered by the bottleneck mentioned earlier. Our layered model of 11 ingredients can identify most of the carbohydrate content in an image in less than 2 seconds, and the number of identifiable ingredients can be scaled up without additional time constraints. Furthermore, we can specifically enhance the recognition of a particular ingredient by adjusting the training data associated with that specific ingredient.

The successful management of diabetes hinges mainly on the patients’ adherence to the dietary recommendations provided by their health care provider [31]. Our innovative miniprogram, FMC, is designed to enhance this adherence by performing a series of tasks in 2 seconds. These tasks include recognizing and recording dietary contents at the ingredient level from images of the patient’s meals and providing immediate feedback on the appropriateness of each recognized ingredient in the patient’s diet. This rapid and efficient process represents a significant improvement over traditional management applications that require manual input, thereby increasing the patient’s commitment to maintaining a consistent dietary log and overall diet [6,32]. Moreover, FMC has the potential to revolutionize the methodology used in assessing patient adherence to dietary recommendations. Current research methods, which predominantly rely on patient questionnaires, often fail to represent accurately the patient’s dietary habits. New digital tools like FMC allow for more precise and rapid patient diet recording. Collecting these data, in turn, enables us to gain a more comprehensive understanding of patient adherence. Furthermore, it provides a more robust platform for studying the impact of various diets, thereby contributing to developing more effective dietary strategies for diabetes management.

### Limitations

Although we have shown that advanced LLM like ChatGPT demonstrated promising results in the registered dietitian exam, responding to frequently asked questions and providing food recommendations, it is essential to note that the model was exposed to only some possible questions a patient might ask. In addition, it can generate different answers to the same question. These limitations are inherent in any AI model training process, as predicting and including every potential query is impossible. An effective strategy to mitigate the concern about the LLM’s ability to respond to all possible patient questions at this stage could be to define a specific scope of questions that the model is trained and validated to handle. By focusing on common and critical nutritional queries, we can ensure that the LLM provides accurate and reliable responses within this defined scope. As the model continues to learn and evolve, the range of questions it can handle can be gradually expanded. This approach allows us to maintain control over the quality of the LLM’s responses while still benefiting from its ability to process and analyze language at scale.

### Conclusions

Our positive results advocate for a clinical pilot study to advance our AI nutritionist program. This study should evaluate its effectiveness in a real-world clinical setting, focusing on patient adherence to dietary recommendations and the subsequent impact on their health outcomes. Furthermore, the pilot study should explore the potential of our AI nutritionist program to serve as a tool for continuous patient education, providing patients with timely and accurate nutritional information to help them make informed dietary decisions. Regarding safety measures, the AI nutritionist program must be limited to providing nutritional information and dietary recommendations. Additionally, all generated responses should be subject to human expert review within 48 hours. Upon discovery, the patient should receive immediate notification if an incorrect response is generated. This approach ensures the safety and accuracy of the information provided while maximizing the potential benefits of the AI nutritionist program.

## Appendix

Multimedia Appendix 1 The questionnaire results and option distribution.

Multimedia Appendix 2 Results of the Chinese registered dietitian examination (ChatGPT and GPT 4.0).

## Abbreviations

AI: artificial intelligence
DSME: diabetes self-management education
FMC: FoodMed Companion
LLM: large language model
MNT: medical nutrition therapy
T2DM: type 2 diabetes mellitus
ViT: vision transformer
