# Supplementary material for: An AI Dietitian for Type 2 Diabetes Mellitus Management Based on Large Language and Image Recognition Models: Preclinical Concept Validation Study
Source: J Med Internet Res. 2023 Nov 9;25:e51300. doi: 10.2196/51300 (PMC10667983; doi:10.2196/51300)
Supplement: Multimedia Appendix 1 [file jmir_v25i1e51300_app1.doc]

**Multimedia Appendix 1**

**Contents:**

Table S1 Patient questionnaire results

Table S2 Endocrinology clinical doctor questionnaire results

Table S3 ChatGPT's answer about Ketogenic diet (food recommended by experts)

Table S4 ChatGPT's answer about Ketogenic diet (food not advised not to eat by experts)

Table S5 The answers to 28 common questions in the ketogenic diet therapy by ChatGPT and experts

**Table S1 Patient questionnaire results**

| **No** | **Question** | **number** | **Frequency(%)** |
| --- | --- | --- | --- |
| 1 | Which of the following is the course of your diabetes? |  |  |
|  | A. Pre-diabetes | 114 | 55.34 |
|  | B．Within 5 years of diabetes diagnosis | 35 | 16.99 |
|  | C．Diabetes diagnosed for more than 5 years (without complications) | 34 | 16.50 |
|  | D．Diabetes diagnosed for more than 5 years (with complications) | 23 | 11.17 |
| 2 | Which of the following do you think is the most effective for controlling diabetes? |  |  |
|  | A．Adjustment of mind | 9 | 4.37 |
|  | B．Medication control | 68 | 33.01 |
|  | C．Diet control | 103 | 50.00 |
|  | D．Exercise management | 26 | 12.62 |
| 3 | Do you agree with the view that unhealthy eating habits are one of the main causes of diabetes |  |  |
|  | A．Agree | 176 | 85.44 |
|  | B．Disagree | 6 | 2.91 |
|  | C．Not sure | 24 | 11.65 |
| 4 | Do you make dietary changes due to health problems? |  |  |
|  | A．Yes | 191 | 92.72 |
|  | B．No | 3 | 1.46 |
|  | C．I am not sure | 12 | 5.83 |
| 5 | How do you see the role of diet management in diabetes management? |  |  |
|  | A．Diet management is the foundation, and diet management itself can significantly lower blood glucose | 143 | 69.42 |
|  | B．Once you start medication, you don't need to manage your diet | 2 | 0.97 |
|  | C．Dietary management is complementary to medication | 61 | 29.61 |
| 6 | Can you exercise 5 or more times a week for 30-60min each time? |  |  |
|  | A．Yes | 82 | 39.81 |
|  | B．No | 49 | 23.79 |
|  | C．I am not sure | 75 | 36.41 |
| 7 | Do you not exercise because you are busy or for other reasons? |  |  |
|  | A．Yes | 133 | 64.56 |
|  | B．No | 30 | 14.56 |
|  | C．I'm not sure | 43 | 20.87 |
| 8 | How much do you spend (directly or indirectly) on diabetes each year? |  |  |
|  | A．Under ¥1,000 | 102 | 49.51 |
|  | B． ¥1,000-2,000 | 28 | 13.59 |
|  | C． ¥2000-3000 | 17 | 8.25 |
|  | D．More than ¥3000 | 59 | 28.64 |
| 9 | Are you aware of any hospitals in China that have developed clinical practice of diet therapy for diabetes? |  |  |
|  | A．Yes | 78 | 37.86 |
|  | B．Don't know | 128 | 62.14 |
| 10 | Do you know the glycemic index (GI) of food? |  |  |
|  | A．Yes | 89 | 43.20 |
|  | B．Not really | 117 | 56.80 |
| 11 | In order to control blood sugar, which diet do you think is the most effective in lowering sugar? |  |  |
|  | A. Regular diet but less fatty and spicy | 43 | 20.87 |
|  | B．Low-carb diet | 132 | 64.08 |
|  | C．Ketogenic diet | 16 | 7.77 |
|  | D．Other diets | 15 | 7.28 |
| 12 | Which of the following foods do you think is most likely to raise blood sugar? |  |  |
|  | A．Beef | 8 | 3.88 |
|  | B．Pasta | 101 | 49.03 |
|  | C．Sweet potatoes | 94 | 45.63 |
|  | D．Tofu | 3 | 1.46 |
| 13 | Which of the following foods do you think is most likely to raise blood sugar? |  |  |
|  | A．Pork belly | 96 | 46.60 |
|  | B．Chinese Yam | 80 | 38.83 |
|  | C．Carrot | 28 | 13.89 |
|  | D．Onion | 2 | 0.97 |
| 14 | Do you feel that the doctors in the hospital provided you with enough diet-related education? |  |  |
|  | A．They can fully assess the patient's condition and give reasonable and detailed dietary advice | 76 | 38.35 |
|  | B．Give a basic understanding of the patient's condition and give general dietary directions | 92 | 44.66 |
|  | C. Only give a few types of contraindications to the patient's condition | 33 | 16.02 |
|  | D．No mention of diet-related precautions | 5 | 2.43 |
| 15 | Does the diet education/information you currently receive meet your diet management needs? |  |  |
|  | A．Basically satisfied | 110 | 53.40 |
|  | B．Partially satisfied | 84 | 40.78 |
|  | C．Not valuable for my dietary management | 12 | 5.83 |
| 16 | Studies show that diet management can be effective in improving blood sugar. If there was a dietitian service that could monitor whether you eat every meal properly, provide you with guidance as recommended by your doctor, and answer any nutrition-related questions you may have 24 hours a day, would you like to use it? |  |  |
|  | A．Yes | 182 | 88.35 |
|  | B．No | 24 | 11.65 |
| 17 | What is the monthly cost you are willing to invest in this service? |  |  |
|  | A．Less than 50¥ | 47 | 22.82 |
|  | B．50¥-100¥ | 56 | 27.18 |
|  | C．100¥-200¥ | 54 | 26.21 |
|  | D．At any cost | 49 | 23.79 |

**Table S2 Endocrinology clinical doctor questionnaire results**

| **No** | **Question** | **number** | **Frequency(%)** |
| --- | --- | --- | --- |
| 1 | Which of the following do you think is the most effective for controlling diabetes? |  |  |
|  | A．Adjustment of mind | 1 | 3.85 |
|  | B．Medication control | 10 | 38.46 |
|  | C．Diet control | 14 | 53.85 |
|  | D．Exercise management | 1 | 3.85 |
| 2 | Do you agree with the view that unhealthy eating habits are one of the main causes of diabetes |  |  |
|  | A．Agree | 25 | 96.15 |
|  | B．Disagree | 0 | 3.85 |
|  | C．Not sure | 1 | 0.00 |
|  | D．Exercise management | 26 | 12.62 |
| 3 | How do you see the role of diet management in diabetes management? |  |  |
|  | A．Diet management is the foundation, and diet management itself can significantly lower blood glucose | 22 | 84.62 |
|  | B．Once you start medication, you don't need to manage your diet | 0 | 0.00 |
|  | C．Dietary management is complementary to medication | 4 | 15.38 |
| 4 | Do you know the glycemic index (GI) of food? |  |  |
|  | A．Yes | 23 | 88.46 |
|  | B．Not really | 3 | 11.54 |
| 5 | Which of the following foods do you think is most likely to raise blood sugar? |  |  |
|  | A．Beef | 0 | 0.00 |
|  | B．Pasta | 16 | 61.54 |
|  | C．Sweet potatoes | 10 | 38.46 |
|  | D．Tofu | 0 | 0.00 |
| 6 | Which of the following foods do you think is most likely to raise blood sugar? |  |  |
|  | A．Pork belly | 6 | 23.08 |
|  | B．Chinese Yam | 17 | 65.38 |
|  | C．Carrot | 3 | 11.54 |
|  | D．Onion | 0 | 0.00 |
| 7 | Do you think you can provide enough diet related education for patients in the process of diagnosis and treatment? |  |  |
|  | A．Can comprehensively evaluate the patient's condition and give reasonable and detailed dietary suggestions | 9 | 34.62 |
|  | B. Can basically understand the patient's condition and give the general direction of diet | 13 | 50.00 |
|  | C．There are too many patients. According to the condition, only a few kinds of taboos that cannot be eaten are given | 4 | 15.38 |
|  | D．Never mention dietary precautions | 0 | 0.00 |
| 8 | Do you think the dietary education / information received by most diabetic patients can meet their dietary management needs? |  |  |
|  | A．Basically satisfied | 5 | 19.23 |
|  | B．Partially satisfied | 16 | 61.54 |
|  | C．Not satisfied | 5 | 19.23 |
| 9 | If there is an artificial intelligence dietitian service that can monitor whether the patient eats each meal reasonably, provide guidance to the patient according to the dietary plan you have formulated, and answer any nutrition related questions of the patient within 24 hours, are you willing to recommend it to the patient? |  |  |
|  | A．Yes | 26 | 0.00 |
|  | B．No | 0 | 0.00 |

**Table S3** ChatGPT's answer about Ketogenic diet (food recommended by experts)

| **ID** | ***Question*** | **ChatGPT answer** |
| --- | --- | --- |
| 1 | *Whether to promote pork in a ketogenic diet* | Pork is a widely accepted and common source of protein and fat on a ketogenic diet. The following are considerations regarding the consumption of pork on a ketogenic diet: Fat content: Pork provides a moderate amount of fat, both saturated and monounsaturated fats. Fat is one of the main sources of energy in a ketogenic diet. Choosing high quality pork, such as leaner cuts and parts of pork with a lower proportion of fat, can help control fat intake. Protein content: Pork is rich in good quality protein, which is important for maintaining muscle mass and meeting the body's protein requirements. Nutritional value: Pork is rich in many vitamins and minerals, such as vitamin B12, iron, zinc and selenium. These nutrients are essential in a ketogenic diet. Serving style: In a ketogenic diet, you can choose to pan-fry, grill, stir-fry or roast pork and avoid adding seasonings high in sugar or starch. However, it is important to note that different people may react differently to food. Some people may be sensitive to pork or have difficulty digesting it. In addition, pork also contains saturated fat, which may increase the risk of heart disease if consumed in excess. Therefore, moderate intake of pork in a ketogenic diet is recommended and should be adjusted according to individual nutritional needs and health status. It is best to consult a professional dietitian or doctor for personalized advice and guidance to ensure a balanced and appropriate nutritional intake on a ketogenic diet. |
| 2 | *Whether to promote beef consumption under ketogenic diet conditions* | In a ketogenic diet, beef is often widely recommended as an important source of protein and fat. Beef is rich in high quality protein and fat and low in carbohydrates, which makes it an ideal choice in line with the principles of the ketogenic diet. When choosing beef, give preference to fat-rich areas such as lean and fatty cuts of meat such as rib eye, tendon and brisket. These parts contain more fat and help provide the energy needed for a ketogenic diet. However, everyone has different meat preferences and individual differences, so other meat options such as chicken, fish and seafood can be chosen according to individual tastes and preferences, if they are in line with the principles of the ketogenic diet, which are low in carbohydrates, moderate in protein and high in fat. It is important to note that while the ketogenic diet promotes a high fat food intake, it still needs to be kept in moderation and balanced with other vegetables, nuts, seeds, and healthy fat sources such as olive oil and coconut oil. In addition, if you have specific health conditions or allergies to certain foods, it is advisable to consult a medical professional or dietitian for advice. |
| 3 | *Is lamb recommended in a ketogenic diet?* | Lamb is often considered to be one of the recommended sources of protein and fat in a ketogenic diet. Lamb is rich in high quality protein and fat and low in carbohydrates, which makes it compatible with the principles of the ketogenic diet.  When choosing lamb, you can give preference to fat-rich parts such as lamb chops, shoulder, or leg. These parts usually contain more fat and provide the energy source needed for a ketogenic diet. However, individual tastes and preferences may vary, so you can choose other meats such as beef, chicken, fish, or seafood depending on your personal preference, if they are in line with the principles of the ketogenic diet, which are low in carbohydrates, moderate in protein and high in fat. It is important to note that while the ketogenic diet encourages the intake of high-fat foods, this still needs to be moderate and balanced with other foods such as low-carb vegetables, nuts, seeds and healthy fat sources (e.g. olive oil, coconut oil, etc.). If you have specific health conditions or are allergic to certain foods, it is advisable to consult a medical professional or dietitian for advice on the most suitable dietary choices for you. |
| 4 | *Whether chicken is recommended on a ketogenic diet* | Chicken is often widely recommended as an important source of protein and fat in a ketogenic diet. Chicken is rich in high-quality protein and relatively low in fat among meats, which makes it a good choice for a ketogenic diet. When choosing chicken, give preference to fat-rich parts such as thighs, wings, or feet. These parts are relatively high in fat and help provide the energy needed for a ketogenic diet. In addition, chicken breasts are a low-fat, high-protein option for those who wish to control their fat intake. Although chicken breast is low in fat, it is still a good source of protein and can be consumed in combination with other high-fat foods (e.g. olive oil, nuts, seeds, etc.) to meet the needs of a ketogenic diet. It is important to note that in addition to chicken, other meats such as beef, lamb, fish or seafood can be chosen for the ketogenic diet, as long as they are in line with the principles of the ketogenic diet, which are low in carbohydrates, moderate in protein and high in fat. Most importantly, maintain a varied diet that combines the intake of other low-carb vegetables, healthy fats and moderate amounts of protein to ensure you get the full range of nutrients. If you have specific health conditions or are allergic to certain foods, it is advisable to consult a medical professional or dietitian for advice and personalized guidance. |
| 5 | *Is duck meat recommended on a ketogenic diet?* | Under ketogenic conditions, duck meat can often be used as a source of protein and fat. Duck meat is rich in high quality protein and fat and has a relatively high fat content in meat, which makes it compatible with the principles of a ketogenic diet. When choosing duck meat, opt for parts that contain more fat, such as the skin of a duck leg or breast. These parts are rich in fat and help provide the energy needed for a ketogenic diet. As with other meats, duck meat can be combined with other high-fat foods (such as olive oil, nuts and seeds) in a ketogenic diet to meet the needs of the diet. It is important to note that individual tastes and preferences may vary, so you can choose other meats such as chicken, beef, lamb, fish or seafood according to your personal preference, as long as they are in line with the principles of the ketogenic diet, which are low in carbohydrates, moderate in protein and high in fat. It is important to maintain a varied diet on a ketogenic diet, combining the intake of other low-carb vegetables, healthy fats and moderate amounts of protein to ensure you get the full range of nutrients. If you have specific health conditions or are allergic to certain foods, it is advisable to consult a medical professional or dietitian for advice and personalized guidance. |
| 6 | *Whether to promote the consumption of goose meat under ketogenic diet conditions* | Under a ketogenic diet, goose meat can be a source of protein and fat. Goose is rich in high quality protein and fat and has a relatively high fat content among meats, which makes it compatible with the principles of a ketogenic diet. When choosing goose meat, opt for parts that contain more fat, such as the skin of the leg or breast of the goose. These parts are rich in fat and help provide the energy needed for a ketogenic diet. As with other meats, goose can be combined with other high-fat foods (such as olive oil, nuts and seeds) in a ketogenic diet to meet the needs of the diet. It is important to note that individual tastes and preferences may vary, so you can choose other meats such as chicken, beef, lamb, duck, fish or seafood according to your personal preference, as long as they are in line with the principles of the ketogenic diet, which are low in carbohydrates, moderate in protein and high in fat. It is important to maintain a varied diet on a ketogenic diet, combining the intake of other low-carb vegetables, healthy fats and moderate amounts of protein to ensure you get the full range of nutrients. If you have specific health conditions or are allergic to certain foods, it is advisable to consult a medical professional or dietitian for advice and personalized guidance. |
| 7 | *Is fish promoted in a ketogenic diet?* | Fish is widely recommended as an important source of protein and fat in a ketogenic diet. Fish is rich in high quality protein and healthy fats and low in carbohydrates, making it an ideal choice for a ketogenic diet. Fish is particularly rich in omega-3 fatty acids, which are important for heart health and inflammation control. Types of fish rich in fat include salmon, cod, tuna and trout. These fish provide not only high-quality protein and fat, but also a wide range of important vitamins and minerals. Also, for a ketogenic diet, it may be better to choose deep-sea fish rather than fatty fish, as deep-sea fish are usually higher in fat and rich in omega-3 fatty acids. In addition to deep-sea fish, other low-fat fish such as sea bass, snapper, chinook salmon and salmon trout are also good choices. Try to keep your diet pure by choosing fresh or frozen fish and avoid adding too much flavoring or sauce. Overall, fish is a great source of good quality protein and fat in a ketogenic diet and can be a healthy choice in your daily diet. However, individual tastes and preferences may vary, so different types of fish can be chosen according to individual preferences and combined with other foods that are consistent with the principles of the ketogenic diet to maintain nutritional balance and variety. |
| 8 | *Whether shrimp is recommended in a ketogenic diet* | Shrimps are often recommended as a good source of protein and fat in a ketogenic diet. Shrimps are rich in high quality protein and relatively low in fat, which makes them compatible with the principles of a ketogenic diet. Shrimps are also a low-carb option and are therefore widely accepted in ketogenic diets. They are also rich in many important vitamins and minerals such as vitamin B12, selenium and iodine. It is important to note that shrimps are relatively low in fat and therefore may require additional fat supplementation in a ketogenic diet. You can eat foods high in fat, such as olive oil, butter or nuts, with shrimp to meet fat requirements. Prawns are also a good food to combine with other ingredients. You can combine prawns with low-carb vegetables, healthy fats and moderate amounts of protein to keep your diet varied and nutritionally balanced. When choosing prawns, it is best to choose fresh or frozen prawns and avoid adding too much seasoning or sauce to keep your diet pure. Most importantly, individual tastes and preferences may vary and if you have specific health conditions or allergies to certain foods, it is advisable to consult a medical professional or dietitian for personalized guidance. |
| 9 | *Is it advisable to eat crab on a ketogenic diet?* | Crab can often be used as a source of protein and fat in a ketogenic diet. Crab meat is rich in high-quality protein and relatively low in fat, which makes it compatible with the principles of a ketogenic diet. Crab meat is also a low-carb option and is therefore widely accepted in ketogenic diets. They are rich in many important vitamins and minerals such as vitamin B12, selenium and zinc. It is important to note that crab meat is relatively low in fat and therefore may require additional fat supplementation in a ketogenic diet. You can serve high-fat foods such as olive oil, butter or nuts with crab meat to meet fat requirements. Crab meat can also be paired with other ingredients such as low carb vegetables, healthy fats and moderate amounts of protein to maintain a varied and nutritionally balanced diet. When choosing crab, it is best to choose fresh or frozen crab and avoid adding too much seasoning or sauce to keep the diet pure. However, it is important to note that the high sugar content in crabmeat and crab yolk requires moderation when it comes to controlling carbohydrate intake. In addition, some people may be allergic to crab meat, so if you have a specific health condition or are allergic to crab, it is advisable to seek advice from a medical professional or dietitian for personalized guidance. |
| 10 | *Whether shellfish is recommended on a ketogenic diet* | Shellfish is usually considered one of the good choices in line with the dietary principles under a ketogenic diet. Shellfish include clams, scallops, razor clams and oysters, which are rich in high-quality protein and healthy fats and are low in carbohydrates. Shellfish are also a rich source of many important nutrients such as vitamin B12, iron, zinc and selenium. They provide the body with the nutrients it needs, while helping to meet protein requirements. Shellfish can be used as one of the main sources of protein in a ketogenic diet and can be combined with other high-fat foods (such as olive oil, butter and nuts) to meet the energy requirements of the diet. When choosing shellfish, it is best to choose fresh or frozen shellfish and avoid adding too much flavoring or sauces to keep the diet pure. It is important to note that some shellfish can trigger allergic reactions, so if you are allergic to shellfish or have a specific health condition, it is advisable to consult a medical professional or dietitian for personalized guidance. Overall, shellfish is a nutritious and principled food on a ketogenic diet and can be a great source of protein and healthy fats, helping to maintain a varied and balanced diet. |
| 11 | *Are eggs promoted in a ketogenic diet?* | Eggs are often seen as one of the best foods to eat on a ketogenic diet. Eggs are rich in high-quality protein and healthy fats and contain almost no carbohydrates, which makes them ideal for a ketogenic diet. The egg yolk is rich in nutrients including vitamin A, vitamin D, vitamin E, vitamin K, B vitamins, choline, lutein and selenium. Protein, on the other hand, is one of the main nutrients in eggs, providing the body with essential amino acids. Eggs are a very versatile ingredient and can be used as a breakfast, lunch or dinner staple. You can serve them boiled, scrambled, in omelets, make egg salads and many other ways. It is important to note that egg yolks contain a certain amount of cholesterol, so for some people, consuming large amounts of eggs may need to be controlled. However, current research suggests that the cholesterol in egg yolks has a low impact on blood cholesterol levels in most people. In summary, eggs are a good source of protein and fat in a ketogenic diet and are rich in many nutrients. You can combine eggs with other low-carb ingredients (e.g. vegetables, high-fat foods, etc.) according to your personal preference to maintain a varied and balanced diet. |
| 12 | *Are duck eggs recommended on a ketogenic diet?* | Duck eggs can often be one of the egg choices on a ketogenic diet. Similar to eggs, duck eggs are rich in high quality protein and healthy fats, and are virtually carbohydrate free. Duck eggs have a few differences compared to eggs. The yolk of the duck egg is relatively large and contains more fat and is rich in nutrients such as vitamin A, vitamin D, vitamin E, vitamin K, B vitamins, choline, lutein and selenium. The protein content of duck eggs is similar to that of eggs and provides the amino acids required by the body. Although there are some differences in nutritional content between duck eggs and eggs, duck eggs are often considered edible in a ketogenic diet. You can consume duck eggs in a variety of ways such as boiling them, frying them and making a duck egg salad. However, it is important to note that some people may be allergic to duck eggs or they may not be readily available in certain areas. Therefore, if you are allergic to duck eggs or have limitations in obtaining them, you can choose other available eggs or other egg types to satisfy your egg needs. In summary, duck eggs can be used as an alternative to eggs in a ketogenic diet and are rich in protein, fat and many nutrients. Depending on individual taste and availability, you can choose duck eggs as part of your diet to maintain nutritional balance and variety. For any specific health conditions or allergic reactions, please consult a medical professional or dietitian for advice. |
| 13 | *Are quail eggs recommended on a ketogenic diet?* | Quail eggs can often be one of the egg choices on a ketogenic diet. Quail eggs are smaller than eggs and duck eggs, but they are also rich in high quality protein and healthy fats, and contain almost no carbohydrates. The nutritional content of quail eggs is similar to that of eggs, including protein, fat and a wide range of vitamins and minerals. Despite their small size, quail eggs have a high nutrient density, so they can be used as one of the sources of nutrients in a ketogenic diet. You can boil quail eggs, fry them, make a quail egg salad or add them to other low carb ingredients to increase your protein and fat intake. It is important to note that the quantity of quail eggs is relatively small, so you need to consider the appropriate amount when calculating your nutritional intake. If you wish to increase your egg intake, you may need to consume more quail eggs or combine them with other egg types. In summary, quail eggs can be used as an alternative choice to eggs in a ketogenic diet, providing protein, fat and a wide range of nutrients. Depending on individual taste and availability, you can choose quail eggs as part of your diet to maintain nutritional balance and variety. If there are any specific health conditions or allergic reactions, please consult a medical professional or dietitian for advice. |
| 14 | *Is it advisable to consume broccoli under a ketogenic diet?* | Broccoli is one of the most widely recommended vegetable choices on a ketogenic diet. Broccoli is a low-carbohydrate and low-calorie food that is also rich in fiber, vitamin C, vitamin K, folic acid and other nutrients. The fiber in broccoli helps to increase satiety and is important for maintaining good digestive function. In addition, it contains a variety of antioxidants such as flavonoids and sulphides, which have anti-inflammatory and anti-cancer potential. Broccoli is also a good choice for a ketogenic diet as it is relatively low in carbohydrates and its fiber content helps with net carbohydrate counting. You can serve broccoli as a main course or side dish with other high-fat foods (e.g. olive oil, butter) to increase satiety and nutritional value. You can steam, boil, stir-fry or roast broccoli, depending on your personal preference. When preparing broccoli, try to avoid sauces that are high in sugar or starch to keep it low in carbohydrates. In conclusion, broccoli is one of the popular vegetables on the ketogenic diet that is nutritious and low in carbohydrates. Combining broccoli with other ketogenic-friendly ingredients can help you maintain a varied and balanced diet. |
| 15 | *Are green leafy vegetables recommended on a ketogenic diet?* | Green leafy vegetables are one of the highly recommended foods on a ketogenic diet. Green leafy vegetables are usually low in carbohydrates and calories and are rich in fiber, vitamins, minerals and antioxidants. Green leafy vegetables such as spinach, lettuce, kale, beet leaves, romaine lettuce and kale provide a wealth of nutrients including vitamin A, vitamin C, vitamin K, folic acid, iron and potassium. At the same time, they are relatively low in carbohydrates, which helps maintain a ketogenic state. Due to their high fiber content, leafy greens help to increase satiety, regulate blood sugar levels and promote good digestion. In addition, they have anti-inflammatory and antioxidant properties that help protect the body from the risk of chronic diseases. On a ketogenic diet, you can use leafy greens as your main vegetable choice. You can use them as a base for salads with high-fat sauces (such as olive oil or yoghurt dressing), eggs, nuts or high-fat meat products. Alternatively, leafy greens can be steamed, stir-fried, boiled or added to soups. It is important to note that different green leafy vegetables vary slightly in carbohydrate content and nutrient content. If you need to watch your carbohydrate intake when calculating net carbohydrates, please refer to the Nutrition Information Sheet or consult a professional for advice. In conclusion, green leafy vegetables are highly recommended foods in a ketogenic diet. They provide a wealth of nutrients and help maintain the principles of a low-carb diet while providing fiber and other healthy ingredients. Combining green leafy vegetables with other ingredients suitable for a ketogenic diet will maintain a varied and nutritionally balanced diet. 16. |
| 16 | *Is it advisable to consume winter squash on a ketogenic diet?* | Winter melon is a moderate vegetable choice to be consumed on a ketogenic diet. Winter melon is relatively low in carbohydrates and is rich in water, fiber, vitamin C and minerals. Despite its low carbohydrate content, winter squash derives its carbohydrates mainly from the sucrose it contains. Therefore, the sucrose content needs to be taken into account when calculating net carbohydrates. The high fiber content of winter melon helps increase satiety, promotes proper digestion and helps regulate blood sugar levels. In addition, winter melon is diuretic, which helps to eliminate excess water and waste. On a ketogenic diet, you can consume winter melon in moderation. The best way to do this is to add winter melon to other low-carb ingredients, such as meat, fish or nuts that are high in fat. Winter melon can be cubed or sliced and steamed, stewed, sautéed or roasted. It is important to note that individual reactions to the carbohydrates in winter squash may vary. For some people, the carbohydrate content of winter squash may have an impact on ketogenic status. Therefore, if you are pursuing a strict ketogenic diet, you may need to control your intake of winter melon or consult with a professional. In summary, winter melon is a vegetable that is moderately consumed on a ketogenic diet. It is rich in water, fiber and some nutrients, but the sucrose content needs to be taken into account when calculating your carbohydrate intake. Depending on individual circumstances and dietary goals, winter squash can be included as part of the vegetable selection to maintain a varied and nutritionally balanced diet. For any specific health conditions, consult a medical professional or dietitian for advice. |
| 17 | *Is it advisable to consume white radish under ketogenic diet conditions?* | White radish can be one of the vegetables consumed in moderation on a ketogenic diet. White radish is a low-carbohydrate vegetable, rich in fiber, vitamin C, vitamin K, minerals and antioxidants. White radish is relatively low in carbohydrates and high in fiber. This makes it one of the vegetables to choose from in a ketogenic diet. The fiber helps to increase satiety, improve digestion and regulate blood sugar levels. White radish also has some antioxidant and anti-inflammatory properties, which may be very beneficial to health. It also contains some minerals, such as potassium and calcium, which help to maintain normal body functions. On a ketogenic diet, you can consume white radish in moderation. It can be eaten raw or added to cooking, such as sliced and added to salads, steamed, stewed, sautéed or roasted. If you are concerned about net carbohydrate intake, you need to be aware of the carbohydrate content of white radish in order to calculate it sensibly. It is important to note that individuals may respond differently to the carbohydrate in white radish. Some people may be more sensitive to the carbohydrate content of white radish and may need to do so with a controlled intake or in consultation with a professional. To conclude, white radish is one of the vegetables consumed in moderation on a ketogenic diet. It is rich in fiber, vitamins and minerals and is relatively low in carbohydrates. Depending on individual circumstances and dietary goals, white radish can be used as part of the vegetable selection to maintain a varied and nutritionally balanced diet. For any specific health conditions, consult a medical professional or dietitian for advice. |
| 18 | *Is olive oil recommended in a ketogenic diet?* | Olive oil is one of the highly recommended sources of fat in a ketogenic diet. Olive oil is a healthy fat, consisting mainly of monounsaturated fatty acids, the most important of which is oleic acid. Rich in antioxidants and compounds with anti-inflammatory properties, such as polyphenols and vitamin E, olive oil is thought to help reduce the risk of chronic inflammation, improve cardiovascular health and is associated with other health benefits. In a ketogenic diet, olive oil is an ideal fat choice as it is mainly fat and free of carbohydrates. The fat content of olive oil helps provide energy and helps maintain a feeling of satiety. It also helps to absorb fat-soluble vitamins and other fat-soluble nutrients. You can use olive oil as a cooking oil or condiment in a ketogenic diet. It can be used for sautéing, grilling, chilling vegetables and making salad dressings. Olive oil has a mellow flavour and can add a delicious taste to food. It is important to note that olive oil is a high fat food, providing 9 calories of energy per gram of fat. Therefore, while controlling your total energy intake, you need to use olive oil wisely according to your individual energy needs and dietary goals. In conclusion, olive oil is a highly recommended source of fat in a ketogenic diet. It is rich in healthy monounsaturated fatty acids and antioxidants, which are associated with a variety of health benefits. The use of olive oil for cooking and flavouring provides nutrition and flavour and helps to maintain the principles of the ketogenic diet. For any specific health conditions, consult a medical professional or dietitian for advice. |
| 19 | *Is the consumption of tea seed oil promoted under ketogenic diet conditions?* | Under ketogenic diet conditions, tea seed oil can be a moderate source of fat for consumption. Tea seed oil is an oil extracted from the seeds of the tea tree and is rich in polyunsaturated fatty acids, particularly linoleic and linolenic acids. The fatty acid composition of tea seed oil makes it a healthy choice. While polyunsaturated fatty acids are beneficial for cardiovascular health, linoleic and linolenic acids are essential fatty acids that the body cannot synthesise on its own and need to be obtained through the diet. Tea seed oil also contains vitamin E and other antioxidants, which have antioxidant and anti-inflammatory properties. It also has some anti-bacterial and anti-fungal properties. In a ketogenic diet, tea seed oil can be used as a source of fat for cooking and flavouring. It has a high smoke point and is suitable for use in high temperature cooking methods such as stir-frying, deep-frying food and roasting. In addition, tea seed oil has a relatively mild flavour and does not affect the taste of food too much. It should be noted that tea seed oil has a relatively high fat content, providing 9 calories of energy per gram of fat. Therefore, while controlling the total energy intake, tea seed oil needs to be used wisely according to individual energy requirements and dietary goals. In summary, tea seed oil is a moderate source of fat for consumption in a ketogenic diet. It is rich in polyunsaturated fatty acids, vitamin E and antioxidants, and has many health benefits. Cooking and flavouring with tea seed oil can provide nutrition and flavour while meeting the principles of the ketogenic diet. Consult a medical professional or dietitian for advice on any specific health conditions. |
| 20 | *Is coconut oil recommended for consumption on a ketogenic diet?* | Coconut oil is one of the commonly recommended sources of fat on a ketogenic diet. Coconut oil consists mainly of saturated fatty acids, of which about 90% are medium-chain triglycerides (MCT). The MCT in coconut oil can be quickly converted into an energy supply and is not easily stored as fat. This makes coconut oil ideal for use in a ketogenic diet, which emphasises high fat intake and low carbohydrate intake. Coconut oil also has a number of other potential health benefits. It can provide some antibacterial and anti-inflammatory effects, which can help support immune system function. In addition, coconut oil can provide some antioxidants such as vitamin E. You can use coconut oil as a cooking oil or cooking oil in a ketogenic diet. It is suitable for high-temperature cooking methods such as stir-frying, roasting and braising. Coconut oil has a strong flavour and can add a special flavour to food. It is important to note that coconut oil is high in saturated fatty acids. Although the ketogenic diet promotes high fat intake, excessive intake of saturated fatty acids may be associated with cardiovascular health. Therefore, it is recommended to moderate your intake when using coconut oil and to balance other sources of fat in your diet. In conclusion, coconut oil is one of the recommended sources of fat in a ketogenic diet, especially due to its MCT content. It can provide energy and has some potential health benefits. When using coconut oil, please moderate your intake and ensure that you are getting a balanced diet. For any specific health conditions, please consult a medical professional or dietitian for advice. |
| 21 | *Is the consumption of safflower seed oil promoted under ketogenic diet conditions* | Safflower seed oil, also known as safflower oil or safflower seed oil, is an oil extracted from the seeds of safflower (Carthamus tinctorius). Its fatty acid composition consists mainly of unsaturated fatty acids, both monounsaturated and polyunsaturated. In a ketogenic diet, safflower seed oil can be used as a moderate source of fat for consumption. It is mainly rich in linoleic acid, an essential polyunsaturated fatty acid that the body cannot synthesise on its own and needs to be obtained through the diet. Safflower seed oil also contains a certain amount of vitamin E and antioxidants, which are important for maintaining cell health and fighting free radical damage. You can use safflower seed oil for cooking and flavouring. Its high smoke point makes it suitable for high-temperature cooking methods such as stir-frying, roasting and braising. Safflower seed oil has a relatively mild flavour and does not affect the taste of food too much. It should be noted that safflower seed oil has a high fat content, providing 9 calories of energy per gram of fat. Therefore, while controlling the total energy intake, safflower seed oil needs to be used in moderation according to individual energy requirements and dietary goals. In summary, safflower seed oil is a moderate source of fat for consumption in a ketogenic diet. It is rich in linoleic acid and other healthy ingredients that provide polyunsaturated fatty acids and antioxidants to the diet. Proper use of safflower seed oil for cooking and flavouring can satisfy fat requirements and add flavour. For any specific health conditions, consult a medical professional or dietitian for advice. |
| 22 | *Is it advisable to consume butter on a ketogenic diet?* | In a ketogenic diet, butter is often widely recommended and used. Butter is the fatty part of dairy products and consists mainly of saturated fatty acids and a small proportion of unsaturated fatty acids. One of the reasons for butter's popularity in a ketogenic diet is its high fat content. The ketogenic diet emphasises a high fat, low carbohydrate intake, and butter provides a rich source of fat. Each gram of fat provides 9 calories of energy, so a moderate intake of butter helps to meet the fat requirements of a ketogenic diet. Butter is also rich in fat-soluble vitamins such as vitamin A, vitamin D, vitamin E and vitamin K. These vitamins are essential for the proper functioning of the body. However, it is important to note that butter is primarily a source of saturated fatty acids. Although the results of recent studies on saturated fatty acids vary, some studies suggest that a high intake of saturated fatty acids may be associated with cardiovascular health. Therefore, when consuming butter, it is advisable to moderate your intake and to ensure that you obtain a balanced diet. In addition, if you have problems such as lactose intolerance or milk protein allergy, you need to be aware that butter is a dairy product and may contain lactose and milk protein. Some people may need to choose a lactose-free or dairy protein-free alternative, such as vegetable-based butter. In summary, butter is a high-fat food commonly used in ketogenic diets. It meets fat requirements and provides fat-soluble vitamins. However, moderate control of intake combined with a balanced diet is required to achieve an optimal nutritional balance. If there are any specific health conditions, please consult a medical professional or dietitian for advice. |
| 23 | *Is lard promoted in a ketogenic diet?* | Lard is usually one of the advocated sources of fat in a ketogenic diet. Lard is a fat derived from pork and consists mainly of saturated fatty acids. The ketogenic diet emphasises a high fat, low carbohydrate intake, and lard provides a rich source of fat. Each gram of fat provides 9 calories of energy, so a moderate intake of lard can help meet the fat requirements of a ketogenic diet. Lard also contains some fat-soluble vitamins such as vitamin E. In addition, it can be used for high-temperature cooking such as stir-frying and pan-frying as it has a high smoke point. However, it is important to note that lard is primarily a source of saturated fatty acids. Although the results of studies on saturated fatty acids have varied in recent years, certain studies have suggested that high intake of saturated fatty acids may be associated with cardiovascular health. Therefore, when consuming lard, it is advisable to moderate your intake and ensure that you obtain a balanced diet. In addition, if you have other health conditions such as high blood pressure or high cholesterol, you may need to consult your doctor or dietitian for advice on choosing a source of fat. To conclude, lard is one of the high-fat foods commonly used in a ketogenic diet. It can meet fat requirements and is used in high-temperature cooking. However, moderate control of intake combined with a balanced diet is required to achieve the best nutritional balance. If there are any specific health conditions, consult a medical professional or dietitian for advice. |
| 24 | *Is sugar-free, dregs-free soymilk recommended under a ketogenic diet?* | In a ketogenic diet, sugar-free and dregs-free soya milk is often considered a suitable beverage choice. Soya milk is a liquid made from blended and strained soybeans and is rich in protein, fat and some carbohydrates. The reasons why sugar-free, dregs-free soya milk is suitable for a ketogenic diet are as follows: Low carbohydrate content: A ketogenic diet emphasises limiting carbohydrate intake, whereas sugar-free, dregs-free soya milk usually contains fewer carbohydrates without added sugar. Moderate fat content: The relatively low fat content of sugar-free, dregs-free soya milk does not usually have a significant impact on the fat restriction of a ketogenic diet. Protein source: Soya milk is a good source of plant-based protein and for some vegetarians or those who do not like animal protein, sugar-free, dregs-free soya milk can provide protein. However, it is important to note that sugar-free, dregs-free soya milk may not be suitable for everyone. Some people may be allergic to soya or have digestive problems, so you need to be aware of your personal physical reactions when choosing soya milk. In addition, although soya milk is a plant-based source of protein, it is not a complete protein and lacks some essential amino acids. Therefore, other sources of protein such as meat, fish, poultry and dairy products need to be combined in a ketogenic diet to obtain an adequate supply of protein. In summary, sugar-free and dregs-free soya milk can be a moderate beverage choice in a ketogenic diet, but attention needs to be paid to the individual's body response and the balance of protein intake. Please consult a medical professional or dietitian for advice on any specific health conditions. |
| 25 | *Whether to promote the consumption of tender/watery tofu in a ketogenic diet* | Tender or watery tofu is usually one of the foods considered suitable in a ketogenic diet. Tofu is a soy product made from soybeans and is rich in protein and some fat. The following are reasons why tender/watery tofu is suitable for a ketogenic diet: Low carbohydrate content: A ketogenic diet emphasises limiting carbohydrate intake, and tofu is relatively low in carbohydrates, especially tender or watery tofu. Protein source: Tofu is a good source of plant-based protein, and for some vegetarians or those who do not like animal protein, tender or buff tofu can provide protein. Moderate fat content: Tofu is relatively low in fat, which can meet the fat requirements of a ketogenic diet. However, it is important to note that each person's body type and the requirements of a ketogenic diet may be different. Some people may be allergic to soya or have digestive problems, so you need to be aware of your individual body's reactions when choosing tofu. In addition, tofu is not a complete source of protein and lacks some essential amino acids. Therefore, other sources of protein such as meat, fish, poultry and dairy products need to be combined in a ketogenic diet to obtain an adequate supply of protein. In summary, tender tofu or buffalo tofu can be a moderate food choice in a ketogenic diet, but attention needs to be paid to the individual's body response and the balance of protein intake. If there are any specific health conditions, please consult a medical professional or dietitian for advice. |
| 26 | *Is the consumption of whole, plain milk (≤200ml per day) advocated under ketogenic diet conditions* | In a traditional ketogenic diet, high carbohydrate milk intake, including whole, plain milk, is usually discouraged because of the relatively high content of lactose (a carbohydrate) in milk. The ketogenic diet focuses on a low-carbohydrate, high-fat dietary pattern. However, dietary patterns vary according to individual needs and variants of the ketogenic diet exist. Some people may include a small amount of whole, plain milk in their ketogenic diet, but limit their intake to 200ml per day. This is done to control carbohydrate intake and to meet individual taste and dietary needs. It is important to note that whole, plain milk contains protein and some micronutrients in addition to fat and lactose. Fat provides satiety and energy, while lactose is a carbohydrate that can affect ketogenic status. Therefore, you should be aware of the carbohydrate content of your overall diet when consuming whole, pure milk. If you are considering consuming whole, pure milk as part of a ketogenic diet, it is advisable to consult a dietitian or doctor for advice. They can provide more specific guidance based on your individual circumstances and ensure that your dietary regimen is in line with your health goals. |
| 27 | *Is unsweetened yoghurt promoted in a ketogenic diet?* | In a ketogenic diet, unsweetened sour milk is often considered a suitable beverage choice. Unsweetened sour milk is milk that has had lactose (a carbohydrate in milk) removed and fermented with a culture of acidophilus. Here are some of the reasons why sugar-free yoghurt is suitable for the ketogenic diet: Low carbohydrate content: The ketogenic diet emphasises limiting carbohydrate intake, and sugar-free yoghurt is usually lactose-free or very low in lactose, thus reducing carbohydrate intake. Fat source: Unsweetened sour milk is usually fermented from whole milk, which provides a moderate amount of fat and helps to meet the fat requirements of the ketogenic diet. Protein source: Unsweetened sour milk is still a form of milk that provides protein and can be used as a plant-based protein substitute for vegetarians or those who do not like animal protein. Unsweetened sour milk also has probiotic benefits, which are also helpful in maintaining intestinal health and digestive function. However, it is important to note that each person's body and metabolism may respond differently to a ketogenic diet. Some people may be allergic to or have digestive problems with the lactose or milk proteins in dairy products. Therefore, when choosing unsweetened yoghurt milk, one needs to be aware of one's body's reaction. In summary, unsweetened sour milk is usually a moderate beverage choice for a ketogenic diet that provides protein, fat and probiotics, while limiting carbohydrate intake. If you have a specific health condition or are concerned about the effects of certain ingredients, consult a medical professional or dietitian for advice. |
| 28 | *Is it advisable to consume mushrooms on a ketogenic diet?* | Mushrooms are often considered as one of the appropriate food choices under ketogenic diet conditions. Mushrooms are a low-carbohydrate, low-sugar food and can therefore be consumed as part of a vegetable in a ketogenic diet. Here are the reasons why mushrooms are suitable for a ketogenic diet: Low carbohydrate content: Mushrooms are relatively low in carbohydrates, especially red and white mushrooms. This makes them ideal for use in a ketogenic diet. Dietary fiber: Mushrooms are rich in dietary fiber, which helps to provide a sense of satiety and is beneficial for digestive health. Vitamins and minerals: Mushrooms are a natural source of vitamin D. They also contain vitamin B, vitamin C and minerals such as iron, potassium and selenium. Low calorie: Mushrooms are relatively low in calories, making them suitable for controlling energy intake. It is important to note that the use of mushrooms in a ketogenic diet also depends on individual carbohydrate restriction and diet plan. Some mushroom varieties may be higher in carbohydrates and therefore need to be chosen with care. Overall, mushrooms are one of the nutrient-rich, low-carb foods that can be consumed as part of a ketogenic diet as a vegetable. As with other ingredients, it is advisable to plan meals according to individual needs and health conditions, and to consult a dietitian or doctor for advice when needed. |
| 29 | *Is the consumption of kelp promoted in a ketogenic diet?* | The intake of kelp should be moderate under ketogenic diet conditions. Kelp is a seaweed that is rich in dietary fiber, vitamins, minerals and other nutrients. The following are considerations regarding kelp in a ketogenic diet: Carbohydrate content: The carbohydrate content of kelp is relatively high, especially the content of algal gel (a polysaccharide). Therefore, care needs to be taken when consuming kelp in a ketogenic diet to control the amount in order to avoid excessive carbohydrate intake. Dietary fiber: Kelp is a good source of dietary fiber. Dietary fiber contributes to a healthy digestive system, promotes satiety, slows down the absorption of carbohydrates and helps to maintain stable blood sugar. Minerals and vitamins: Kelp is rich in minerals and vitamins such as iodine, calcium, magnesium, iron and vitamin K. These nutrients are important for proper body function and health. It is important to note that although kelp has its nutritional value, excessive intake may interfere with the goals of a ketogenic diet due to its high carbohydrate content. Therefore, moderate intake of kelp in a ketogenic diet is recommended and adjusted according to individual carbohydrate intake restrictions and nutritional needs. It is advisable to consult a professional dietitian or doctor for personalized advice and guidance to ensure that the appropriate amount of kelp is consumed on a ketogenic diet. |
| 30 | *Is it advisable to consume macadamia nuts on a ketogenic diet?* | The edibility of macadamia nuts under ketogenic diet conditions may be limited by their high carbohydrate content. Although macadamia nuts are rich in fat and fiber, they contain about 17 g of carbohydrate per 100 g of macadamia nuts. In a typical ketogenic diet, it is required to limit the amount of total carbohydrates consumed to around 20-50 grams per day in order to put the body into ketosis. Therefore, if you plan to consume macadamia nuts on a ketogenic diet, you need to be careful to control your intake and factor it into your total carbohydrate intake.You can discuss with a dietitian or doctor to determine the individual carbohydrate restriction and diet plan that is right for you. They can provide personalized advice to ensure that you are getting the right nutritional intake and maintaining the required metabolic status on a ketogenic diet. |
| 31 | *Are Brazil nuts promoted in a ketogenic diet?* | Brazil nuts are one of the food choices that can be consumed in moderation on a ketogenic diet. Brazil nuts are rich in healthy fats, protein and fiber and are low in carbohydrates, which makes them an ideal option for a ketogenic diet. Here are the reasons why Brazil nuts are suitable for a ketogenic diet: Fat content: Brazil nuts are rich in fat and can contain over 66 grams per 100 grams. These fats are mainly healthy monounsaturated fats and polyunsaturated fatty acids, which help to provide energy and meet the fat requirements of a ketogenic diet. Protein content: Brazil nuts are also rich in protein, with around 14 grams per 100 grams. Protein is an important nutrient in a ketogenic diet, helping to maintain muscle tissue and a sense of satiety. Fiber content: Brazil nuts contain dietary fiber, which helps to promote digestive health and stabilise blood sugar levels. Nutritional value: Brazil nuts are rich in vitamin E, magnesium, phosphorus and other minerals, which are beneficial for maintaining normal body function. Despite their nutritional benefits, Brazil nuts are recommended to be consumed in moderation due to their high fat and calorie content. A moderate daily intake of Brazil nuts as a snack or added to other foods can help meet fat and protein requirements. Overall, it is possible to consume Brazil nuts in moderation on a ketogenic diet, but remember that individual nutritional needs and health conditions will vary. It is best to consult a professional dietitian or doctor for advice when developing and adjusting your diet plan to ensure that your diet plan is appropriate for your individual situation. |
| 32 | *Is it advisable to consume walnuts on a ketogenic diet?* | Walnuts are a popular and suitable food choice on a ketogenic diet. Walnuts are rich in healthy fats, protein and fiber and are low in carbohydrates, which makes them an ideal option for a ketogenic diet. Here are some of the reasons why walnuts are suitable for a ketogenic diet: Fat content: Walnuts are rich in fat, which can reach over 60 grams per 100 grams. These fats are mainly healthy polyunsaturated fatty acids, such as omega-3 fatty acids, which are good for heart health. Protein content: Walnuts are also rich in protein, with around 15 grams per 100 grams. Protein is an important nutrient in a ketogenic diet, helping to maintain muscle tissue and a sense of satiety. Fiber content: Walnuts contain dietary fiber, which helps to promote digestive health and stabilise blood sugar levels. Nutritional value: Walnuts are rich in vitamin E, magnesium, manganese and other minerals, which are beneficial for maintaining normal body function. It is feasible to consume walnuts in moderation as a high-fat, low-carb snack on a ketogenic diet. You can eat walnuts on their own as a snack or add them to salads, baked goods or other diets. However, walnuts are also a high energy density food, providing approximately 650 calories per 100g. Therefore, it is important to consume walnuts in moderation while controlling overall energy intake. The bottom line is that individual nutritional needs and health conditions will vary. It is best to consult a professional dietitian or doctor for advice when developing and adjusting your diet plan to ensure that your diet plan is suitable for your individual situation. |
| 33 | *Is the consumption of tomatoes (tomatoes) recommended on a ketogenic diet?* | Tomatoes (tomatoes) can be consumed in moderation on a ketogenic diet. Tomatoes are a low-carbohydrate, low-calorie vegetable, containing only about 3-4 g of carbohydrate per 100 g of tomato. Here are the reasons why tomatoes are suitable for a ketogenic diet: Low carbohydrate content: Tomatoes are relatively low in carbohydrates, which makes them suitable for a ketogenic diet. Moderate consumption of tomatoes can provide some fiber and vitamins while controlling overall carbohydrate intake. Nutritional value: Tomatoes are rich in vitamin C, vitamin A, vitamin K and antioxidants, which are essential for maintaining good health. Moisture content: Tomatoes are high in water, which helps to keep the body hydrated and balanced. Low calorie content: Tomatoes are low in calories, at around 18-20 calories per 100g. This makes tomatoes one of the lighter options in the ketogenic diet. However, it is important to note that although tomatoes are low in carbohydrates, you still need to manage your intake wisely on a ketogenic diet. Everyone has a different tolerance to carbohydrates, so it is best to tailor your diet plan to your individual health status and goals when developing and adjusting it. In summary, tomatoes can be consumed in moderation on a ketogenic diet. As a low-carb, low-calorie vegetable, tomatoes provide some important nutrients and hydration. However, please remember to consume tomatoes in moderation according to your individual needs and goals within the context of overall carbohydrate restriction and adjust according to your own response. If necessary, seek the advice of a professional dietitian or doctor to ensure that your diet plan is appropriate for your individual situation. |
| 34 | *Is the consumption of cucumber advocated under ketogenic diet conditions* | Cucumber is an excellent vegetable choice under ketogenic diet conditions. Cucumber is a low-carb, low-calorie food, containing only about 2-3 grams of carbohydrates per 100 grams of cucumber. Here's why cucumber is suitable for a ketogenic diet: Low carbohydrate content: Cucumber is very low in carbohydrates, which makes it ideal for use in a ketogenic diet. Eating cucumber in moderation can provide some fiber and water while controlling overall carbohydrate intake. Water content: Cucumber is very high in water, which helps to keep the body hydrated and balanced while helping to provide a feeling of satiety. Nutritional value: Cucumber is rich in vitamin K, vitamin C and some minerals such as potassium and magnesium. These nutrients are essential for maintaining a healthy and properly functioning body. Low calorie content: Cucumber is very low in calories, at around 15-20 calories per 100g. This makes cucumber one of the low energy density options in a ketogenic diet. Cucumber can be used as the vegetable part of a ketogenic diet and can be eaten raw, added to salads or used as a garnish. However, cucumber needs to be consumed in moderation according to individual health status and goals, and adjusted within the context of overall carbohydrate restriction. In summary, cucumber is a great vegetable choice for the ketogenic diet. The low carbohydrate, low calorie and high-water content make it one of the ideal ketogenic vegetables. However, please consume cucumber in moderation according to your individual health needs and goals, and adjust according to your own response. If necessary, seek advice from a professional dietitian or doctor to ensure that your diet plan is suitable for your individual situation. |
| 35 | *Is it advisable to consume plain water on a ketogenic diet?* | In a ketogenic diet, plain water is one of the most ideal beverages. With no sugar, carbohydrates or calories, plain water is important for maintaining water balance and meeting the body's water needs. Here's why plain water is suitable for the ketogenic diet: Zero sugar and carbohydrates: plain water contains no sugar or carbohydrates, which is in line with the ketogenic diet's principle of limiting carbohydrate intake. No calories: plain water is zero calorie and does not contribute to the energy intake in the diet. Maintaining water balance: Drinking enough water is important to maintain the body's water balance, boost metabolism and maintain kidney function. On a ketogenic diet, it is especially important to ensure adequate hydration. Although plain water is the ideal drink on a ketogenic diet, this does not mean that you should only drink plain water. You can also choose to drink decaffeinated tea, plain water, water mixed with lemon juice or water with certain sugar-free flavourings added. However, it is important to remember that each person's water needs vary according to individual differences, environmental conditions, and physical activity levels. It is vital to ensure that you drink enough water each day to meet your individual hydration needs. To summarise, plain water is one of the most suitable drinks on a ketogenic diet as it has no sugar, carbohydrates or calories. Maintaining adequate water intake is vital to the success of a ketogenic diet, so make sure you drink enough water each day, adjusting it to your individual needs. |
| 36 | *Is the consumption of light tea water promoted under ketogenic diet conditions* | Under ketogenic diet conditions, light tea water can be consumed in moderation. Light tea is usually defined as tea without any added sugar or other additives, such as green tea, white tea, black tea and oolong tea. Here's why light tea water is suitable for the ketogenic diet: Low carbohydrate content: Light tea water usually contains no sugar or only trace amounts of sugar, so it can be used as a low-carb drink option in the ketogenic diet. Antioxidants: tea is rich in antioxidants such as tea polyphenols and catechins, which are beneficial for health. Stimulates metabolism: The caffeine and theophylline in tea can increase metabolism and help with fat burning. Provides hydration: Light tea water can be an option for hydration and helps to keep the body hydrated and balanced. It is important to note that some teas may contain high levels of caffeine, which may have a stimulating effect for some people. If you are sensitive to caffeine or have a specific health condition, please consult your doctor for advice before drinking. Also, if you like to add lemon or other citrus fruit slices to your tea, be aware that citrus fruits may contain a certain amount of carbohydrates. Make sure you add a moderate amount to avoid exceeding your carbohydrate limit. In summary, light tea water is a drink that can be consumed in moderation on a ketogenic diet. It has the advantage of being low in carbohydrates, providing antioxidants, boosting metabolism and providing hydration. However, there are individual differences, so please consume light tea water in moderation according to individual needs and goals, and consult a professional dietitian or doctor for advice when needed. |
| 37 | *Is it advisable to consume unsweetened coffee on a ketogenic diet?* | Sugar-free coffee can be consumed in moderation under a ketogenic diet. Sugar-free coffee refers to coffee with no added sugar or other high carbohydrate content. Here's why sugar-free coffee is suitable for the ketogenic diet: Low carbohydrate content: Pure black coffee has no carbohydrates of its own and can therefore be used as a low-carb drink option on the ketogenic diet. However, if you add milk or dairy products, you need to be aware that they may contain some carbohydrates. Caffeine boosts: The caffeine in coffee can boost the mind and increase alertness and concentration. This is helpful for staying energised and focused on a ketogenic diet. Antioxidants: Coffee is rich in antioxidants, such as polyphenols and bioketones, which help fight free radical damage. Fat metabolism: The caffeine in coffee is thought to stimulate fat metabolism and fat oxidation, helping to increase energy expenditure and fat burning. It is important to note that everyone has different tolerances and sensitivities to caffeine. Some people may be allergic to caffeine or have a negative reaction to it. In addition, if you consume coffee at night it may affect the quality of your sleep, so it is best to consume it at an appropriate time to avoid disrupting your sleep. To conclude, unsweetened coffee is a drink that can be consumed in moderation on a ketogenic diet. It is low in carbohydrates and provides a caffeine boost, antioxidant substances and a possible stimulation of fat metabolism. However, please consume it in moderation according to individual tolerance and needs, and consult a professional dietitian or doctor for advice if needed. |
| 38 | *Is broth recommended on a ketogenic diet?* | Broth is one of the foods promoted in a ketogenic diet. Broth is a soup made from meat, bones or other ingredients of animal origin that have been cooked and stewed. Here's why broths are suitable for a ketogenic diet: Low carbohydrate: Broths usually contain no or only trace amounts of carbohydrates, especially when no starchy ingredients are added. High in protein: The meat and bones in broth provide high quality protein, which helps maintain muscle mass and a sense of satiety. Nutrient-rich: The meat and bones simmered in the broth release nutrients such as collagen, amino acids, minerals, and vitamins that are beneficial to your health. Replenishes electrolytes: Broth contains a certain amount of electrolytes such as sodium, potassium and magnesium, which help maintain water-electrolyte balance. In addition, broths can also provide a soothing effect and may be helpful for indigestion or for comfort when you have a cold. When choosing a broth, it is best to choose one that is made from natural ingredients without the addition of extra flavourings, starches, or sugars. Chicken broth, beef bone broth and fish broth are all common types of broth, so you can choose the right meat and bones for your personal preference and nutritional needs. To conclude, broth is one of the foods that is promoted in the ketogenic diet. It is low in carbohydrates, high in protein, nutritious and offers the benefit of replenishing electrolytes. Choose homemade broths and ensure that the ingredients are pure and meet your personal taste and health needs. It is also advisable to consult a professional nutritionist or doctor for advice if required. |

Table S4 ChatGPT's answer about Ketogenic diet (experts do not recommend food)

| **ID** | ***Question*** | **ChatGPT answer** |
| --- | --- | --- |
| 1 | *Under the conditions of a ketogenic diet, is it recommended to consume candies?* | Generally speaking, it is not recommended to consume candies on a ketogenic diet. The ketogenic diet is a high-fat, low-carbohydrate dietary approach designed to stimulate the body to enter a state of ketosis (where the body burns fat for energy) by reducing carbohydrate intake. Since candies typically contain simple carbohydrates and sugars, these foods can cause an increase in blood sugar levels, preventing the body from maintaining ketosis.  Furthermore, the goal of a ketogenic diet is to promote fat metabolism in the body. Therefore, it is recommended to consume foods high in fat, moderate in protein, and low in carbohydrates, such as nuts, meat, fish, dairy products, and low-starch vegetables. Consuming candies may result in an excessive intake of carbohydrates and sugars, affecting the maintenance of ketosis and potentially leading to weight gain and insulin sensitivity rebound.  If you are considering a ketogenic diet, it is advisable to consult a nutritionist or doctor to ensure that your diet is reasonable, balanced, and aligned with your specific health needs and condition. |
| 2 | *Under the conditions of a ketogenic diet, is it recommended to consume pastries?* | Generally speaking, it is not recommended to consume pastries on a ketogenic diet. The ketogenic diet is a high-fat, moderate-protein, low-carbohydrate dietary approach designed to stimulate the body to enter a state of ketosis (where the body burns fat for energy) by reducing carbohydrate intake. Pastries typically contain a significant amount of carbohydrates, especially sugars and starches, which can cause an increase in blood sugar levels, preventing the body from maintaining ketosis.  Furthermore, the goal of a ketogenic diet is to promote fat metabolism in the body. Therefore, it is recommended to consume foods high in fat, moderate in protein, and low in carbohydrates, such as nuts, meat, fish, dairy products, and low-starch vegetables. Consuming pastries may result in an excessive intake of carbohydrates and sugars, affecting the maintenance of ketosis and potentially leading to weight gain and insulin sensitivity rebound.  If you are considering a ketogenic diet, it is advisable to consult a nutritionist or doctor to ensure that your diet is reasonable, balanced, and aligned with your specific health needs and condition. |
| 3 | *Under the conditions of a ketogenic diet, is it recommended to consume cookies?* | Generally, it is not recommended to consume cookies on a ketogenic diet. A ketogenic diet is a high-fat, moderate-protein, low-carbohydrate dietary approach designed to stimulate the body to enter a state of ketosis (where the body burns fat for energy) by reducing carbohydrate intake. Cookies typically contain a significant amount of carbohydrates, especially sugars and starches, which can cause an increase in blood sugar levels, preventing the body from maintaining ketosis.  Furthermore, the goal of a ketogenic diet is to promote fat metabolism in the body. Therefore, it is recommended to consume foods high in fat, moderate in protein, and low in carbohydrates, such as nuts, meat, fish, dairy products, and low-starch vegetables. Consuming cookies may result in an excessive intake of carbohydrates and sugars, affecting the maintenance of ketosis and potentially leading to weight gain and insulin sensitivity rebound.  If you are considering a ketogenic diet, it is advisable to consult a nutritionist or doctor to ensure that your diet is reasonable, balanced, and aligned with your specific health needs and condition. |
| 4 | *Under the conditions of a ketogenic diet, is it recommended to consume ice cream?* | Generally, it is not recommended to consume ice cream on a ketogenic diet. The principles of a ketogenic diet involve high fat, moderate protein, and low carbohydrates, while ice cream typically contains a significant amount of sugar and carbohydrates, causing an increase in blood sugar levels and preventing the body from maintaining ketosis. Additionally, ice cream may contain fats and proteins, but it may not provide sufficient dietary fat and protein. It may also contain additives and artificial colors that are not beneficial for health.  If you are considering a ketogenic diet, it is advisable to avoid consuming ice cream and instead choose other low-carbohydrate, high-fat foods such as nuts, meat, fish, dairy products, and low-starch vegetables. If you have any concerns or questions about adjusting your diet, it is recommended to consult a nutritionist or doctor to ensure that your diet is reasonable, balanced, and aligned with your specific health needs and condition. |
| 5 | *Under the conditions of a ketogenic diet, is it recommended to consume rice?* | It is not recommended to consume rice on a ketogenic diet. The principles of a ketogenic diet involve high fat, moderate protein, and low carbohydrates, while rice is a high-carbohydrate food that can cause an increase in blood sugar levels, preventing the body from maintaining ketosis. Additionally, rice lacks sufficient dietary fat and protein.  If you are considering a ketogenic diet, it is advisable to choose other low-carbohydrate, high-fat foods such as nuts, meat, fish, dairy products, and low-starch vegetables. If you need to consume carbohydrates, you can consider selecting other low-carbohydrate grains or legumes. It is also recommended to consult a nutritionist or doctor to ensure that your diet is reasonable, balanced, and aligned with your specific health needs and condition. |
| 6 | *Under the conditions of a ketogenic diet, is it recommended to consume noodles?* | It is not recommended to consume traditional noodles on a ketogenic diet because they are primarily made of starch and are high in carbohydrates, causing an increase in blood sugar levels and making it difficult for the body to maintain ketosis. Additionally, traditional noodles lack sufficient dietary fat and protein.  However, there are low-carbohydrate alternatives to noodles available. For example, you can use vegetables such as cucumber, pumpkin, or zucchini, cut into thin strips to create vegetable noodles, or use tofu or shirataki noodles made from tofu or konjac as substitutes. These low-carbohydrate options can serve as alternatives in a ketogenic diet while providing some dietary fiber and nutrients.  Overall, a ketogenic diet suggests limiting carbohydrate intake but allows for the selection of other low-carbohydrate alternatives to satisfy cravings while ensuring dietary balance and nutrient intake. |
| 7 | *Under the conditions of a ketogenic diet, is it recommended to consume flour?* | On a ketogenic diet, traditional flour-based foods are not encouraged because they are typically made from high-carbohydrate ingredients such as wheat flour and starch, which can cause an increase in blood sugar levels and make it difficult for the body to maintain ketosis.  However, there are low-carbohydrate alternatives to flour, such as almond flour, coconut flour, flaxseed flour, etc. These low-carbohydrate options can serve as substitutes in a ketogenic diet while providing dietary fat, protein, and some nutrients.  Overall, a ketogenic diet suggests limiting carbohydrate intake but allows for the selection of other low-carbohydrate alternatives to satisfy cravings while ensuring dietary balance and nutrient intake. |
| 8 | *Under the conditions of a ketogenic diet, is it recommended to consume whole grains and their products?* | Whole grains and their products typically contain higher amounts of carbohydrates, so they are generally not encouraged to be consumed on a strict ketogenic diet. For example, grains like rice, wheat, corn, glutinous rice, oats, etc., are high in carbohydrates and are not suitable for a ketogenic diet.  However, in a ketogenic diet, you can choose low-carbohydrate grain substitutes or alternatives such as coconut flour, almond flour, cheese flour, etc. These substitutes can not only satisfy your cravings but also provide dietary fat, protein, and some nutrients.  In conclusion, if you are strictly following a ketogenic diet, whole grains and their products should be limited. However, you can opt for low-carbohydrate substitutes to replace high-carbohydrate foods while maintaining ketosis and meeting your dietary needs. |
| 9 | *Under the conditions of a ketogenic diet, is it recommended to consume processed meat products like sausages?* | Processed meat products like sausages often contain higher levels of fat and protein, but they may also include additives and seasonings, some of which might have small amounts of carbohydrates. Therefore, in a ketogenic diet, it is important to choose sausages that are low in carbohydrates and avoid those with added sugars or high-carbohydrate seasonings.  Specifically, cured meat products such as bacon, ham, and cured sausages are generally considered suitable sources of high-fat and high-protein for a ketogenic diet. However, please note that these products often contain additives such as preservatives and flavorings, so it's best to choose natural products without additives and seasonings.  In general, while processed meat products like sausages may not be the ideal foods for a ketogenic diet, they can be consumed in moderation if you carefully select low-carbohydrate options and avoid additives and seasonings. |
| 10 | *Under the conditions of a ketogenic diet, is it recommended to consume meat floss or similar processed meat products?* | Meat floss is a processed meat product that typically contains higher levels of protein and fat, but it may also have some carbohydrates and additives. Therefore, in a ketogenic diet, it is important to choose meat floss that is low in carbohydrates and avoid those with seasonings that contain sugars.  Specifically, some sugar-free or low-sugar meat floss products may be considered as high-protein, high-fat snacks in a ketogenic diet. However, please note that these products often contain additives such as preservatives and flavorings, so it's best to choose natural products without additives and seasonings.  In general, while meat floss or similar processed meat products may not be the ideal foods for a ketogenic diet, they can be consumed in moderation if you carefully select low-carbohydrate options and avoid additives and seasonings. |
| 11 | *Under the conditions of a ketogenic diet, is it recommended to consume beef jerky?* | Beef jerky is a type of processed meat product that typically contains higher levels of protein and fat, but it may also have some carbohydrates and additives. Therefore, in a ketogenic diet, it is important to choose beef jerky that is low in carbohydrates and avoid those with seasonings that contain sugars.  Specifically, some sugar-free or low-sugar beef jerky products may be considered as high-protein, high-fat snacks in a ketogenic diet. However, please note that these products often contain additives such as preservatives and flavorings, so it's best to choose natural products without additives and seasonings.  In general, while beef jerky may not be the ideal food for a ketogenic diet, it can be consumed in moderation if you carefully select low-carbohydrate options and avoid additives and seasonings. |
| 12 | *Under the conditions of a ketogenic diet, is it recommended to consume canned meat?* | Canned meat products often contain protein and fat, but they may also contain additives and other ingredients such as seasonings and starches. In a ketogenic diet, it is important to choose canned meat that is low in carbohydrates and avoid products that are high in sugars or starches.  Specifically, some sugar-free or low-sugar canned meats may be considered as high-protein, high-fat options in a ketogenic diet. However, please note that these products often contain additives such as preservatives and flavorings, so it's best to choose natural products without additives and seasonings.  In general, while canned meat may not be the ideal food for a ketogenic diet, moderate consumption of low-carbohydrate products can be considered. However, it's important to choose natural products without additives as much as possible |
| 13 | *Under the conditions of a ketogenic diet, is it recommended to consume oyster sauce or other sauces containing sugar?* | Sauces containing sugar should be avoided as much as possible in a ketogenic diet because they typically contain higher levels of carbohydrates and sugars. This includes oyster sauce and other sauces containing sugar. It is recommended to choose oyster sauce products without added sugars and other carbohydrates, and also to control the amount used. |
| 14 | *Under the conditions of a ketogenic diet, is it recommended to consume sweet bean sauce?* | Sweet bean sauce is a sauce that contains sugar and should be avoided as much as possible in a ketogenic diet. It usually contains a high amount of carbohydrates and sugars, which can raise blood sugar levels and go against the principles of a ketogenic diet. If you need to use a sauce, it is recommended to choose low-carbohydrate and low-sugar options or use other low-carbohydrate seasonings as substitutes. |
| 15 | *Under the conditions of a ketogenic diet, is it recommended to consume ketchup?* | Ketchup is a sauce that relatively high in carbohydrates, and therefore not an ideal choice for a ketogenic diet. A serving of 30 grams of ketchup typically contains around 5-6 grams of carbohydrates, which can exceed the desired limit of carbohydrate intake. Therefore, if you are following a ketogenic diet, it is best to limit or avoid consuming ketchup. |
| 16 | *Under the conditions of a ketogenic diet, is it recommended to consume strawberry jam?* | Strawberry jam contains a significant amount of sugar, so it is not recommended to consume it in large amounts in a ketogenic diet. Generally, a ketogenic diet emphasizes a high-fat, low-carbohydrate eating pattern, while strawberry jam typically contains high levels of carbohydrates and sugars, which goes against the principles of a ketogenic diet. It is recommended to choose low-sugar fruits or other low-carbohydrate foods as alternatives. |
| 17 | *Under the conditions of a ketogenic diet, is it recommended to consume potatoes?* | In a ketogenic diet, it is generally not recommended to consume potatoes because they have a high carbohydrate content, which is not conducive to maintaining a state of ketosis. A small portion of potatoes may contain low enough carbohydrates to be consumed in moderation while controlling overall intake, but this would need to be assessed on a case-by-case basis. If you are following a ketogenic diet, it's best to consult with a healthcare professional or a registered dietitian for advice. |
| 18 | *Under the conditions of a ketogenic diet, is it recommended to consume sweet potatoes?* | Sweet potatoes have a higher sugar content compared to regular potatoes, so they are not recommended for consumption in large amounts on a ketogenic diet. Sweet potatoes also have a relatively high glycemic index, which can cause an increase in blood sugar levels and affect ketone production. However, moderate amounts of sweet potatoes can be included as a natural food source that contains fiber and vitamins, serving as an alternative source of carbohydrates in a ketogenic diet. Overall, if you are following a ketogenic diet, it's advisable to reduce the intake of both regular potatoes and sweet potatoes. |
| 19 | *Under the conditions of a ketogenic diet, is it recommended to consume cassava?* | In a ketogenic diet, it is generally not recommended to consume cassava (also known as yam, manioc) in large amounts because it has a high starch and carbohydrate content. In moderate amounts, cassava may be acceptable, but specific recommendations depend on individual circumstances and dietary plans. It's important to note that raw cassava contains toxic cyanide compounds and requires special processing to be safely consumed. |
| 20 | *Under the conditions of a ketogenic diet, is it recommended to consume yam?* | In a ketogenic diet, yam can be consumed in moderate amounts. It contains a small amount of carbohydrates and dietary fiber, and it is rich in vitamins and minerals, which are beneficial for the body. However, it's important to note that yam's carbohydrate content is not very low, so intake should be controlled while following a ketogenic diet. |
| 21 | *Under the conditions of a ketogenic diet, is it recommended to consume taro?* | In a ketogenic diet, it is generally not recommended to consume taro in large amounts. Although taro is rich in dietary fiber and certain nutrients, it has a high starch content and contains a significant amount of carbohydrates, which may affect ketosis if consumed excessively. If you wish to consume taro, it can be done in moderation, but it's important to balance it with other low-carbohydrate ingredients to maintain the effectiveness of the ketogenic diet. |
| 22 | *Under the conditions of a ketogenic diet, is it recommended to consume lotus root?* | In a ketogenic diet, lotus root is relatively low in carbohydrates and can be consumed as a vegetable in controlled amounts. However, it's important to note that lotus root contains a relatively high amount of starch. While it doesn't contain fat, every 100 grams of lotus root contains about 15 grams of carbohydrates, mostly in the form of starch. Therefore, moderate consumption of lotus root may be acceptable while controlling total carbohydrate intake, but the specific amount consumed should be based on individual dietary plans and nutritional needs. |
| 23 | *Under the conditions of a ketogenic diet, is it recommended to consume water chestnuts?* | In a ketogenic diet, water chestnuts can be included as a food source because they primarily consist of water and fiber. Every 100 grams of water chestnuts contain about 4.5 grams of carbohydrates, with most of it being fiber. Thus, water chestnuts are relatively low in carbohydrates and suitable for a ketogenic diet. However, it's important to note that water chestnuts also contain small amounts of glucose and starch, so total carbohydrate intake should still be monitored when consuming them. |
| 24 | *Under the conditions of a ketogenic diet, is it recommended to consume carrots?* | Carrots have a relatively high carbohydrate content. Raw carrots contain approximately 7 grams of carbohydrates per 100 grams, while cooked carrots contain about 8 grams of carbohydrates per 100 grams. Therefore, in a strict ketogenic diet, it is recommended to limit the intake of carrots, especially when consumed raw. However, in more flexible versions of the ketogenic diet, moderate consumption of carrots may be allowed. The specific recommendation may vary depending on individual circumstances and nutritional needs. |
| 25 | *Under the conditions of a ketogenic diet, is it recommended to consume onions?* | In a ketogenic diet, moderate consumption of onions is generally considered acceptable. Onions are low in carbohydrates and sugars, making them a suitable choice for a ketogenic diet. Additionally, onions are rich in nutrients such as vitamin C, B vitamins, potassium, and sulfur. However, it's important to note that carbohydrate intake needs to be limited in a ketogenic diet. Therefore, if a large amount of onions is consumed, it may exceed the recommended carbohydrate intake. It is recommended to consume onions in moderation and combine them with other low-carbohydrate vegetables. |
| 26 | *Under the conditions of a ketogenic diet, is it recommended to consume dried vegetables or dehydrated vegetable products?* | The acceptability of dried vegetables or dehydrated vegetable products in a ketogenic diet can vary depending on the type of vegetables. Generally, these products tend to have low sugar content but often contain higher levels of sodium. Therefore, dried vegetables or dehydrated vegetable products may not be the preferred food choice in a ketogenic diet. If you want to try these products, it's best to check the nutrition facts on the packaging and incorporate them into your accurately measured daily food plan. |
| 27 | *Under the conditions of a ketogenic diet, is it recommended to consume vegetable oil powder?* | In a ketogenic diet, vegetable oil powder can be used as a substitute for plant-based fats to meet the fat requirements. Vegetable oil powder is typically derived from various plant oils, providing a range of fatty acids and is considered a healthier alternative to animal fats. However, vegetable oil powder is still a high-fat, high-calorie food and should be used with caution in a ketogenic diet by controlling the intake. Additionally, some brands of vegetable oil powder may contain added sugar-based ingredients, so it's important to check the ingredient list and nutritional labels when purchasing. |
| 28 | *Under the conditions of a ketogenic diet, is it recommended to consume non-dairy creamer?* | In a ketogenic diet, non-dairy creamer is generally not recommended for consumption. Non-dairy creamer is a low-fat, low-calorie, and lactose-free substitute. However, it often contains a significant amount of artificial additives and chemical ingredients such as artificial sweeteners and preservatives, which may have negative effects on health. Moreover, non-dairy creamer is not a natural food and is not typically considered a healthy choice in a ketogenic diet. It is recommended to opt for natural full-fat milk or dairy products in a ketogenic diet, as they are nutrient-rich and more beneficial for health. |
| 29 | *Under the conditions of a ketogenic diet, is it recommended to consume cocoa butter?* | Cocoa butter can be consumed in moderation as a source of high-fat, low-carbohydrate in a ketogenic diet. It is a vegetable fat extracted from cocoa beans and primarily consists of fatty acids. The fatty acid composition of cocoa butter is suitable for a ketogenic diet, and it contains minimal carbohydrates. Therefore, cocoa butter can be one of the choices in a ketogenic diet when consumed in appropriate amounts. However, it's important not to consume excessive amounts of cocoa butter as it is also high in calories, and overconsumption can affect the effectiveness of the ketogenic diet. |
| 30 | *Under the conditions of a ketogenic diet, is it recommended to consume artificial butter or products containing trans fats?* | It is not recommended to consume foods containing trans fats in a ketogenic diet. Trans fats can increase low-density lipoprotein (LDL) cholesterol levels, decrease high-density lipoprotein (HDL) cholesterol levels, and increase the risk of cardiovascular diseases. Artificial butter and similar products often contain high amounts of trans fats, and therefore, they are not recommended for consumption in a ketogenic diet. |
| 31 | *Under the conditions of a ketogenic diet, is it recommended to consume mung beans?* | Mung beans are a food with a relatively high carbohydrate content. 100 grams of mung beans contain approximately 62 grams of carbohydrates, which makes them less suitable for a ketogenic diet. In a ketogenic diet, it is advised to control carbohydrate intake and usually limit the total daily carbohydrate intake to around 20-50 grams. Therefore, if you want to consume mung beans in a ketogenic diet, you need to control the portion size and consider other foods with lower carbohydrate content to incorporate into your diet. |
| 32 | *Under the conditions of a ketogenic diet, is it recommended to consume red beans?* | In a ketogenic diet, red beans are generally considered high in carbohydrates, and therefore, they are not encouraged to be consumed. If you plan to strictly adhere to a ketogenic diet, it is advisable to limit or avoid the intake of red beans. |
| 33 | *Under the conditions of a ketogenic diet, is it recommended to consume lentils?* | A ketogenic diet focuses on low-carbohydrate and high-fat foods, so the consumption of lentils is generally not encouraged. Lentils are legumes with a relatively high carbohydrate content. 100 grams of lentils contain approximately 17 grams of carbohydrates, mostly in the form of starch. Therefore, if you are following a ketogenic diet, it is advisable to reduce or avoid the consumption of lentils to maintain the goals of a low-carbohydrate diet. |
| 34 | *Under the conditions of a ketogenic diet, is it recommended to consume edamame (green soybeans)?* | In a ketogenic diet, you can consume edamame in moderation, but it's important to be mindful of the portion size due to its carbohydrate content. 100 grams of edamame contains approximately 7 grams of carbohydrates. Therefore, while controlling the overall carbohydrate intake, the amount of edamame consumed should be limited to an appropriate range. It is also recommended to choose unprocessed edamame to avoid excessive additives and seasonings. |
| 35 | *Under the conditions of a ketogenic diet, is it recommended to consume soybeans?* | Soybeans are generally not recommended to be consumed in large quantities in a ketogenic diet because they contain relatively high amounts of carbohydrates and protein, and they have a lower fat content. In some strict ketogenic diet plans, soybeans are often considered high in sugar content. Therefore, it is advised to limit or avoid their consumption. However, moderate amounts of soybean products such as tofu and soy milk can be included as a source of protein, but it's important to be mindful of their carbohydrate and protein content to ensure they do not exceed the limits of an individual's ketogenic diet. |
| 36 | *Under the conditions of a ketogenic diet, is it recommended to consume tofu skin (yuba)?* | Tofu skin, also known as yuba, can be consumed in a ketogenic diet. Tofu skin is a dried product made from baked tofu, mainly composed of soybean protein and fiber. It is rich in dietary fiber and high-quality protein, while containing relatively fewer carbohydrates, making it suitable for a ketogenic diet. However, tofu skin is higher in calories, so it is advisable to consume it in moderation. |
| 37 | *Under the conditions of a ketogenic diet, is it recommended to consume bamboo shoots?* | Bamboo shoots are a food ingredient derived from bamboo and typically require starch processing, resulting in a higher starch content. Therefore, they are not suitable for consumption in a ketogenic diet. The main principle of a ketogenic diet is to limit carbohydrate intake and choose low-carbohydrate ingredients such as vegetables, meats, fish, and nuts. Therefore, it is not recommended to consume bamboo shoots in a ketogenic diet. |
| 38 | *Under the conditions of a ketogenic diet, is it recommended to consume tofu skin (yuba)?* | Tofu skin, or yuba, is edible in a ketogenic diet because it is rich in protein and fiber while being low in carbohydrates and fat. However, it's important to note that tofu skin also contains some plant-based carbohydrates, so it should be considered in moderation as part of your overall dietary plan. Additionally, some processing methods may add sugar or starch, so it's important to check the ingredients. |
| 39 | *Under the conditions of a ketogenic diet, is it recommended to consume flavored and processed dairy products?* | It's generally not recommended to consume flavored and processed dairy products in a ketogenic diet, as these products often contain added sugars and other carbohydrates that can increase the overall net carbohydrate content of your diet, making it challenging to maintain a state of ketosis. Additionally, these products may also contain other ingredients that are not beneficial for health, such as artificial flavors and preservatives. If you want to include dairy in your ketogenic diet, it's best to choose natural, unflavored, high-fat dairy products such as heavy cream, butter, and cheese. |
| 40 | *Under the conditions of a ketogenic diet, is it recommended to consume soy milk?* | Soy milk, which is made from soybeans, is generally higher in carbohydrates and may not be suitable for regular and high consumption in a ketogenic diet. The recommended approach is to limit carbohydrate intake in a ketogenic diet. However, if consumed in moderation and within the appropriate portion size, soy milk can be included as one of the beverage options in a ketogenic diet. Additionally, some low-carbohydrate legumes such as soybeans and black beans can be consumed in moderation, but it's important to consider the intake amount within the appropriate range for a ketogenic diet. |
| 41 | *Under the conditions of a ketogenic diet, is it recommended to consume yogurt with added sugars?* | Generally, it is not recommended to consume yogurt with added sugars in a ketogenic diet because it contains higher amounts of carbohydrates and sugars, which can disrupt the carbohydrate restriction of the ketogenic diet. If you want to consume dairy products, it is advisable to choose full-fat yogurt or unsweetened dairy products. |
| 42 | *Under the conditions of a ketogenic diet, is it recommended to consume dried shiitake mushrooms?* | Dried shiitake mushrooms can be a good ingredient to include in a ketogenic diet as they are rich in protein, fiber, and various vitamins. However, it's important to note that dried shiitake mushrooms have a relatively higher calorie content and contain a certain amount of carbohydrates. Therefore, portion control is important to avoid affecting the effectiveness of the ketogenic diet. It is recommended to consume dried shiitake mushrooms in moderation and be mindful of the intake amount. |
| 43 | *Under the conditions of a ketogenic diet, is it recommended to consume potato chips?* | Generally, potato chips tend to be high in carbohydrates and unhealthy fats, and they have low nutritional value. In a ketogenic diet, it is advised to avoid consuming potato chips and instead choose healthy, low-carbohydrate, high-protein, and healthy-fat foods. |
| 44 | *Under the conditions of a ketogenic diet, is it recommended to consume peanuts?* | Peanuts are not the optimal choice in a ketogenic diet because they contain higher amounts of carbohydrates and protein. One ounce (28 grams) of peanuts contains about 6 grams of carbohydrates, with most of it being starch, and only 2.7 grams of fiber. Additionally, peanuts also contain some protein, but the protein content is not as high as in meats or fish. Therefore, in a ketogenic diet, it may be necessary to limit the intake of peanuts to ensure a balance of low carbohydrates, moderate protein, and high fat intake. |
| 45 | *Under the conditions of a ketogenic diet, is it recommended to consume sunflower seeds?* | Sunflower seeds are not the preferred food in a ketogenic diet because they contain relatively high amounts of carbohydrates and fats, although they are also a healthy plant-based source of protein. When consumed in moderation, sunflower seeds can be chosen as a snack option, but it's important to keep track of the total intake throughout the day to ensure it doesn't exceed the daily allowance of carbohydrates and calorie limits. |
| 46 | *Under the conditions of a ketogenic diet, is it recommended to consume chestnuts?* | Chestnuts are a high-carbohydrate food, containing about 30 grams of carbohydrates per 100 grams of food. Therefore, in a strict ketogenic diet, chestnuts are not an ideal choice. However, if the intake is controlled and carbohydrate consumption is properly managed in the overall daily diet, consuming chestnuts in moderation should not significantly impact ketosis. |
| 47 | *Under the conditions of a ketogenic diet, is it recommended to consume cashews?* | Cashews should be consumed in moderation in a ketogenic diet. While cashews are rich in healthy fats and protein, they also contain relatively high amounts of carbohydrates and a small amount of sugar, which can push carbohydrate intake above the daily limit in a ketogenic diet. Additionally, cashews may also contain mycotoxins, which can be harmful to the body. Therefore, while cashews can be included as a snack or ingredient in a ketogenic diet in moderate amounts, their intake should be controlled. |
| 48 | *Under the conditions of a ketogenic diet, is it recommended to consume dried fruits?* | Generally, it is not recommended to consume dried fruits in large quantities in a ketogenic diet because they are concentrated sources of natural sugars and carbohydrates. Dried fruits have a higher glycemic index compared to fresh fruits, which can potentially affect blood sugar levels and hinder ketosis. If you choose to consume dried fruits, it is important to select options with lower sugar content and limit the portion size to stay within the carbohydrate limits of the ketogenic diet. |
| 49 | *Under the conditions of a ketogenic diet, is it recommended to consume dried fruits?* | In a ketogenic diet, it is generally not recommended to consume dried fruits excessively. Dried fruits are fruits that have had their water content evaporated, resulting in concentrated sugars and higher calorie content. They can easily cause blood sugar spikes, which is not beneficial for maintaining a state of ketosis. Additionally, some dried fruits may contain additives and preservatives that are not conducive to health. If you choose to consume dried fruits, it's best to select options without additives and consume them in moderation. |
| 50 | *Under the conditions of a ketogenic diet, is it recommended to consume fruit preserves?* | In a ketogenic diet, it is not highly recommended to consume fruit preserves. Fruit preserves often contain a significant amount of sugar, and even the fruits themselves can become sweeter due to the addition of sugar during the preservation process. In a ketogenic diet, where carbohydrate intake needs to be limited, consuming fruit preserves can increase the overall carbohydrate intake and potentially lead to blood sugar fluctuations. If you really want to consume fruit preserves, it's advisable to choose options with little to no added sugar and still control the portion size. |
| 51 | *Under the conditions of a ketogenic diet, is it recommended to consume fruit jam?* | Generally, fruit jam is not recommended in a ketogenic diet because it typically contains a high amount of sugar and carbohydrates. Even if natural sweeteners like fruits and beets are used, it is difficult for fruit jam to meet the carbohydrate restrictions of a ketogenic diet. Therefore, if you are following a ketogenic diet, it's best to avoid consuming fruit jam. |
| 52 | *Under the conditions of a ketogenic diet, is it recommended to consume carbonated beverages?* | In a ketogenic diet, it is generally not recommended to consume carbonated beverages because they often contain a significant amount of sugar and high calories, which contradicts the principles of a ketogenic diet. Furthermore, carbonated beverages may also affect ketone production and blood sugar control. It is recommended to choose sugar-free beverages such as water, tea, and coffee instead. |
| 53 | *Under the conditions of a ketogenic diet, is it recommended to consume fruit juice?* | In a ketogenic diet, it is not advisable to consume large quantities of fruit juice because many fruit juices are high in sugar content, which can easily exceed the carbohydrate intake limits of a ketogenic diet. Additionally, most of the fiber in the fruits is removed during the juicing process, resulting in faster absorption and digestion of the sugars in the juice. If you want to consume fruit juice, it's recommended to choose options with low sugar content and mix them with high-fiber vegetables and fruits to slow down the absorption of sugars. The best option is to consume whole fruits instead of fruit juice because the fiber in the fruits slows down the absorption of sugars. |
| 54 | *Under the conditions of a ketogenic diet, is it recommended to consume milk tea?* | A ketogenic diet advocates for restricting carbohydrate intake, and milk tea typically contains a significant amount of sugar and carbohydrates, making it not recommended for a ketogenic diet. If you must have milk tea, you can choose low-sugar or sugar-free options and substitute the sugar and carbohydrates in milk tea with low-carbohydrate alternatives. However, the best choice is to avoid milk tea or other high-sugar beverages as much as possible to prevent compromising the effectiveness of the ketogenic diet. |
| 55 | *Under the conditions of a ketogenic diet, is it recommended to consume white wine?* | Under a ketogenic diet, the restrictions on alcohol consumption can vary from person to person, but it is generally advisable to limit alcohol intake. Although white wine does not contain sugar, it contains high-calorie alcohol. Consuming excessive amounts can lead to weight gain and affect ketosis. Additionally, alcohol consumption can also affect metabolism and detoxification capacity, which may have a negative impact on the effectiveness of the ketogenic diet. Therefore, it is recommended to consume alcohol in moderation or avoid it altogether during a ketogenic diet. |
| 56 | *Under the conditions of a ketogenic diet, is it recommended to consume beer?* | In a ketogenic diet, it is generally not recommended to consume beer in large quantities. Beer contains carbohydrates, especially maltose, which can potentially disrupt the low carbohydrate intake required by a ketogenic diet. Additionally, beer contains alcohol, and excessive consumption can have negative effects on overall health. If you choose to drink alcohol, it is advisable to opt for healthier options such as sake or red wine, but moderation is still key. |
| 57 | *Under the conditions of a ketogenic diet, is it recommended to consume red wine?* | Under a ketogenic diet, moderate consumption of red wine is acceptable. Red wine contains polyphenolic compounds such as anthocyanins, flavonoids, and resveratrol, which have antioxidant properties. It also has relatively low sugar content and can be consumed without significantly affecting ketosis. However, it's important to note that alcohol does contribute calories, and excessive alcohol consumption can increase calorie intake and potentially interfere with weight loss. Therefore, in a ketogenic diet, it is recommended to consume red wine in moderation and keep alcohol intake to less than one glass per day. |

**Table S5** The answers to 28 common questions in the ketogenic diet therapy by ChatGPT and experts

| **ID** | ***Question*** | **Expert answer** | **ChatGPT answer** |
| --- | --- | --- | --- |
| 1 | *What is a low-carbohydrate diet?* | Carbohydrates refer to substances composed of carbon, hydrogen, and oxygen. Carbohydrates in the diet, also known as sugars, mainly include fructose, starch, and fiber, and they are the most inexpensive nutrient. A low-carbohydrate diet is a type of diet that involves consuming fewer carbohydrates, such as those containing fructose and starch. It gained popularity globally due to the publication of the book "The Atkins Diet Revolution" by Dr. Atkins in the United States. Atkins advocated a very low carbohydrate intake and believed that when the body does not receive carbohydrates for energy, it will seek other fuel sources such as proteins or fats. Therefore, this dietary method was initially used for weight loss. However, it is not scientifically sound to solely focus on reducing carbohydrate intake without considering the balance of other nutrients, especially excluding fats. A purely low-carbohydrate diet can often lead to poor mental state, muscle wasting, and nutritional deficiencies. | A low-carbohydrate diet refers to limiting the intake of carbohydrates in food, particularly fast-digesting carbohydrates such as sugar and starch. This diet primarily emphasizes consuming high amounts of protein, high amounts of fat, and a small amount of carbohydrates. The goal of a low-carbohydrate diet is to induce a state of "ketosis," where the body begins to utilize fat as an energy source while reducing carbohydrate intake. A low-carbohydrate diet typically includes foods such as meat, fish, poultry, vegetables, fruits, nuts, and healthy oils like olive oil, coconut oil, and butter. Compared to traditional high-carbohydrate diets, a low-carbohydrate diet can help with weight loss, lower blood sugar levels, improve cardiovascular health, and reduce inflammation. However, it's important to note that a low-carbohydrate diet is not suitable for everyone, especially those with kidney issues, who should consult with a doctor for appropriate adjustments. |
| 2 | *What are ketones?* | Ketones are a collective term for intermediate products of fatty acid oxidation in the liver, including acetoacetate, beta-hydroxybutyrate, and acetone. Once the body ingests enough dietary fat to meet its daily energy needs, the excess portion is converted by the liver into ketones, which serve as a small-molecule fuel reserve for the entire body, including the brain. When the demand for glucose (blood sugar) in the body exceeds supply, such as during a low-carbohydrate ketogenic diet or fasting, the body utilizes stored ketones as an alternative fuel source. Ketones are a clean energy source, as when cells utilize glucose as fuel, they produce various "waste products" such as free radicals. However, when ketones are used as fuel, they only produce carbon dioxide and water, without generating waste. Among ketone types, beta-hydroxybutyrate is the main form found in the bloodstream, accounting for 80% to 90% of total ketone bodies. Research has shown that ketones have antioxidant, anti-inflammatory, and anti-aging effects. | Ketones are compounds produced by the breakdown of fats in the liver when the body has insufficient glycogen (stored glucose) available. They include acetoacetate, beta-hydroxybutyrate, and acetone. These compounds serve as an energy source, particularly during periods of prolonged fasting or high-intensity exercise when glucose is not readily available. In certain circumstances, such as a low-carbohydrate, high-fat diet (e.g., ketogenic diet), ketone levels can also be increased through dietary means. Additionally, in certain conditions such as diabetic ketoacidosis and alcoholic ketoacidosis, ketone levels may be elevated. |
| 3 | *What is a nutritional ketosis state?* | Dietary intake of low-carbohydrate foods is easily broken down into blood glucose. Consuming a high-carbohydrate diet can cause elevated blood sugar levels, leading to a phenomenon known medically as hyperinsulinemia, where the pancreas needs to secrete large amounts of insulin. Over time, the body develops insulin resistance, which can lead to diabetes. It is widely known that individuals with diabetes should avoid consuming "sugar," meaning they must consume fewer foods containing carbohydrates. However, it is important to note that if protein intake is excessive, it can also be converted into blood glucose. Only when fat intake is relatively high can the body convert the excess portion into ketones for storage, which can be released as fuel when needed. Therefore, compared to the threat of carbohydrates and protein causing elevated blood sugar, maintaining a certain level of ketones in the body is much safer. Under normal circumstances, blood ketone levels range from 0.1 to 0.2 mmol/L, and during a ketogenic diet, they can rise to 0.5 to 5.0 mmol/L. This is known as a nutritional ketosis state, which is a normal and healthy state of fat utilization for energy. Generally, blood ketone levels exceeding 6 mmol/L indicate a pathological state. A proper low-carbohydrate ketogenic diet should not only emphasize carbohydrate restriction but also ensure the intake of a certain proportion of protein and fat to keep the body in a state of nutritional ketosis. It is particularly important to emphasize that the intake of fat should be much higher than that of protein. This is the core aspect of a low-carbohydrate ketogenic diet, which is why some people simply refer to this diet as a ketogenic diet. | Nutritional ketosis refers to a state of ketosis achieved through dietary control. It is typically achieved by restricting carbohydrate intake, which lowers blood glucose levels and prompts the liver to break down fat, resulting in the production of ketones for energy. In this dietary pattern, protein and fat become the primary sources of energy, while carbohydrate intake is strictly limited. Nutritional ketosis can be achieved through various diet patterns, including the ketogenic diet, low-carbohydrate high-fat (LCHF) diet, and intermittent fasting. This dietary approach can have potential health benefits such as weight loss, blood sugar control, and reduced cholesterol levels. However, if not implemented correctly or if carbohydrate intake is excessively restricted, it can also have negative effects such as dizziness, fatigue, muscle soreness, and digestive issues. Therefore, it is advisable to follow this dietary pattern under the guidance of a doctor or a professional nutritionist. |
| 4 | *Why advocate for a low-carbohydrate ketogenic diet and what are the benefits?* | The advocacy for a low-carbohydrate ketogenic diet is based on its numerous health benefits. Here are some of the benefits known so far: Weight loss: A low-carbohydrate ketogenic diet can transform your body into a fat-burning machine, leading to significant weight loss. With reduced insulin levels, the rate and extent of fat burning increase. This creates an ideal internal environment for fat reduction without experiencing significant hunger. Extensive scientific research has shown that low-carbohydrate ketogenic diets are more effective for weight loss compared to other dietary patterns. Appetite control: Adopting a low-carbohydrate ketogenic diet can help you better control your appetite. When the body is in a state of burning fat, it taps into its own stored energy for weeks or months, greatly reducing hunger. This not only helps address sugar or food addiction but also improves certain eating disorders such as binge eating. Sustained energy and mental clarity: A low-carbohydrate ketogenic diet provides a continuous supply of energy to the brain. The body converts fat into ketones, which are an energy source that enhances focus, mental clarity, and helps avoid significant blood sugar fluctuations. This can improve attention and alleviate symptoms of "brain fog." Many people experience increased energy levels after adopting a low-carbohydrate ketogenic diet. Blood sugar control and alleviating type 2 diabetes: A low-carbohydrate ketogenic diet can help control blood sugar levels, which is beneficial for managing type 2 diabetes. Research has shown that this type of diet plays a significant role in reducing blood sugar levels and mitigating the negative effects of high insulin levels. Improved health markers: Many studies have shown that a low-carbohydrate ketogenic diet can improve important health indicators, including blood lipid levels, blood sugar levels, insulin levels, and blood pressure. Improved gastric comfort: Adopting a low-carbohydrate ketogenic diet can alleviate symptoms such as stomach spasms, bloating, and pain, leading to a more comfortable feeling. Increased endurance during exercise: A low-carbohydrate ketogenic diet mobilizes stored fat for continuous energy supply, greatly increasing endurance. While glycogen stores (carbohydrate fuel) in the body can only provide energy for a few hours or less during intense exercise, fat stores can easily provide energy for weeks or months. Epilepsy control: Low-carbohydrate ketogenic diets have been used to control epilepsy since the 1920s. Initially used primarily for treating childhood epilepsy, it has gradually expanded to treat adult patients as well. Adopting this diet helps reduce or discontinue the use of antiepileptic drugs, reducing the adverse effects of medication and improving the quality of life for patients. In addition to the common benefits mentioned above, for some individuals, a low-carbohydrate ketogenic diet can also reduce skin acne, alleviate migraines, treat celiac disease, and even aid in the management of certain mental illnesses. Some studies have also suggested that a low-carbohydrate ketogenic diet may extend lifespan, reduce the risk of cancer, and be explored for preventive and therapeutic purposes in Parkinson's disease and Alzheimer's disease. | Low-carb ketogenic diet is a dietary approach that restricts carbohydrate intake while increasing fat and protein intake. In this diet, the body enters a state of ketosis, where it metabolizes fat to produce ketones for energy. The main benefits of advocating for a low-carb ketogenic diet are as follows: Promotes weight loss: A low-carb ketogenic diet can facilitate weight loss by limiting carbohydrate intake, which reduces insulin secretion and enhances fat metabolism. Additionally, it provides satiety, leading to reduced food intake and better calorie control. Improves insulin sensitivity: Adopting a low-carb ketogenic diet can improve the body's sensitivity to insulin, helping to regulate blood sugar and lipid levels. This reduces the risk of metabolic diseases such as diabetes, hypertension, and cardiovascular disorders. Enhances lipid metabolism: A low-carb ketogenic diet can improve lipid metabolism by lowering plasma triglyceride and cholesterol levels while increasing the concentration of high-density lipoprotein cholesterol (good cholesterol) in the blood.  Increases muscle mass: By appropriately increasing protein intake and combining it with moderate exercise, a low-carb ketogenic diet can promote muscle synthesis and increase muscle mass. |
| 5 | *What is the difference between nutritional ketosis and ketoacidosis?* | Ketosis refers to the presence of ketones in the blood, which typically occurs during fasting, low-carbohydrate ketogenic diets, or after excessive alcohol consumption when the body is using fat for energy. Some people associate "ketones" with ketoacidosis and become concerned, but it is unnecessary. Ketoacidosis, also known as diabetic ketoacidosis, is commonly seen in type 1 or poorly controlled type 2 diabetes, or in diabetic patients experiencing severe infections, improper treatment, or stress from injuries, surgeries, or pregnancy. During ketoacidosis, fats are mobilized rapidly, leading to the excessive production of ketones and severe insulin deficiency. As a result, glucose cannot enter the cells, leading to abnormally high blood sugar levels. If the produced ketones exceed the capacity of extrahepatic tissues for utilization and cannot be stored, they accumulate and cause harm. These patients often have significantly elevated levels of ketones and blood sugar (ketones > 15mmol/L, sugar > 13mmol/L) accompanied by severe dehydration. Due to the acidic nature of ketones, metabolic disturbances and acidosis occur, which can progress rapidly and even pose a life-threatening risk within 24 hours, requiring immediate medical intervention. However, rest assured that the production of ketones is fundamentally different from ketoacidosis. They are not the same thing, and mild elevation of ketones can be beneficial for both the mind and body. In individuals with normal insulin function following a low-carbohydrate ketogenic diet, the ketone levels in the blood typically range from 0.5 to 3mmol/L and rarely exceed 5mmol/L. This level of ketosis does not acidify the blood significantly or have a major impact on blood pH. Moreover, maintaining a certain level of blood ketones can be beneficial for improving mental and physical performance and insulin sensitivity. Therefore, we refer to this level of ketosis as "nutritional ketosis," which is a normal physiological response. As you may know, in most people, consuming starchy foods or sugary beverages leads to an increase in blood sugar, triggering insulin secretion to transport the sugar from the bloodstream into cells. However, in the two types of diabetes mentioned earlier, the elevated blood sugar persists due to severe insulin deficiency, resulting in a state of hyperglycemia known as uncontrolled diabetic sugar metabolism. From the perspective of cells, they need a source of energy, and since glucose is unavailable, they are forced to switch to an alternative energy source—ketones. The liver accelerates the breakdown of fats to produce ketones, which cells can utilize as an alternative fuel. In pathological conditions, with a lack of insulin, blood ketone levels can become very high, exceeding the liver's capacity for utilization, which affects blood pH and requires urgent medical attention. However, in individuals with normal insulin function following a ketogenic diet, the increase in blood ketone levels is minimal. Why is that? It's because moderate insulin secretion inhibits ketone production. If blood ketones do not exceed 6mmol/L, it is considered safe.  Although individuals in the prediabetes, early-stage, and mid-stage of diabetes can surprisingly control their blood sugar quickly and even restore it to normal levels through a proper low-carbohydrate ketogenic diet, there is indeed a risk of ketoacidosis for those with type 1 diabetes or severe type 2 diabetes if their insulin secretion is already insufficient. Therefore, if diabetic patients want to follow a low-carbohydrate ketogenic diet, it is recommended for safety reasons to have continuous monitoring of blood sugar and ketone changes by a professional physician and nutritionist. The blood sugar should not exceed 13mmol/L, and the ketone levels should not exceed 6mmol/L. However, this does not mean that all diabetic patients are unsuitable for a low-carbohydrate ketogenic diet. The majority of individuals do not experience any severe issues with their internal body environment, so there is no need to give up on a ketogenic diet that can be beneficial for their health. | Nutritional ketosis and ketoacidosis are two different concepts. Nutritional ketosis is a normal metabolic state in which the body produces ketones from fat metabolism when carbohydrate intake is inadequate or restricted, and utilizes them as an energy source. In this state, the ketone levels in the blood increase but remain within a normal range, and the body does not experience any discomfort or pathological changes. Ketoacidosis, on the other hand, is a pathological condition characterized by excessively high levels of ketones in the blood, leading to increased blood acidity and pathological changes in multiple systems of the body. Ketoacidosis typically occurs in situations such as diabetes, prolonged fasting, or excessive restriction of carbohydrate intake. In these cases, low blood sugar prompts the body to metabolize fat for energy, resulting in the production of a large amount of ketones. However, if the ketone levels become too high and the liver cannot process them in a timely manner, it can lead to increased blood acidity and trigger ketoacidosis. Therefore, nutritional ketosis and ketoacidosis are two different metabolic states, with the former being a normal physiological response and the latter being a pathological condition that requires prompt diagnosis and treatment. When adopting a ketogenic diet or any other dietary approach, it is important to maintain a balanced and appropriate diet to avoid adverse reactions such as ketoacidosis. |
| 6 | *What is the correct nutrient ratio in a low-carbohydrate ketogenic diet?* | Experts suggest that the correct nutrient ratio in a low-carbohydrate ketogenic diet should be as follows: carbohydrates providing 5% of the energy (the lower, the better), protein providing 15% to 25% of the energy (preferably high-quality protein but not excessive), and fat providing at least 70% of the energy. Based on our clinical practice, this ratio of diet is beneficial for health. | The correct nutrient ratio varies from person to person and depends on factors such as individual body condition, age, gender, activity level, and metabolic rate. However, the correct nutrient ratio in a low-carbohydrate ketogenic diet should be approximately as follows: Protein: About 10% to 35% of total energy intake. For the average person, a daily intake of approximately 0.8 grams of protein per kilogram of body weight is recommended. In a low-carbohydrate ketogenic diet, protein intake should be relatively higher to support muscle growth and repair. Fat: About 60% to 80% of total energy intake. In a low-carbohydrate ketogenic diet, fat is the primary source of energy and the raw material for ketone synthesis in the body. It is advisable to choose foods rich in monounsaturated and polyunsaturated fats, such as olive oil and fish oil. Carbohydrates: About 5% to 10% of total energy intake. In a low-carbohydrate ketogenic diet, carbohydrate intake is very low and mainly comes from green leafy vegetables, nuts, and some berries. Furthermore, it is important to ensure an adequate intake of fiber, vitamins, minerals, and other nutrients in a low-carbohydrate ketogenic diet to maintain overall health. To ensure nutritional balance, it is recommended to consult a healthcare professional or dietitian before adopting a low-carbohydrate ketogenic diet. |
| 7 | *Who is a low-carbohydrate ketogenic diet suitable for? Who is it not suitable for?* | A low-carbohydrate ketogenic diet is generally very safe, and it can be adopted by generally healthy and sub-healthy individuals according to their own needs. However, for individuals with diabetes, kidney disease, severe liver disease, or hypertension who intend to use this diet for therapeutic purposes, or for lactating women, children, and adolescents who intend to use this diet to increase nutrition, it is important to seek guidance from a professional physician or nutritionist skilled in low-carbohydrate ketogenic diets. Although most people can benefit from a low-carbohydrate ketogenic diet, strict adherence to it is not suitable for pregnant women. Pregnant women with gestational diabetes, for example, are only suitable for a relatively lenient low-carbohydrate diet. Patients with malnutrition should not mechanically adhere to the proportions of the three major nutrients. The diet should be evaluated and adjusted according to the specific condition of malnutrition. | A low-carbohydrate ketogenic diet is suitable for certain populations but not for others. Here are some examples of who it is suitable for and who it is not suitable for: Suitable for:  Overweight or obese individuals: A low-carbohydrate ketogenic diet can help reduce weight and decrease the accumulation of body fat. Individuals with diabetes: A low-carbohydrate ketogenic diet can help control blood sugar levels and reduce the need for insulin. Hypertensive individuals: A low-carbohydrate ketogenic diet can help lower blood pressure and improve vascular function. Individuals with metabolic syndrome or cardiovascular diseases: A low-carbohydrate ketogenic diet can improve symptoms of metabolic syndrome and cardiovascular diseases. People who want to improve their physique or enhance physical performance: A low-carbohydrate ketogenic diet can increase energy levels and resilience, thereby improving physique and enhancing physical capabilities. Not suitable for: Pregnant and lactating women: A low-carbohydrate ketogenic diet may affect fetal development and the nutritional content of breast milk. Individuals with hypothyroidism: A low-carbohydrate ketogenic diet may impact the synthesis and metabolism of thyroid hormones, leading to decreased thyroid function. Individuals with liver diseases: A low-carbohydrate ketogenic diet may increase the burden on the liver and result in abnormal liver function. Individuals with kidney diseases: A low-carbohydrate ketogenic diet may increase the burden on the kidneys and lead to abnormal kidney function. Children and adolescents: A low-carbohydrate ketogenic diet may affect growth and development, and therefore is not suitable for children and adolescents to adopt. |
| 8 | *How safe is the low-carbohydrate ketogenic diet? Can it be followed for a lifetime?* | The safety of a low-carbohydrate ketogenic diet is generally well-established. Recent research suggests that it is safe to follow this diet in the short term (3-6 months), and many individuals have followed it for up to 2 years without adverse effects. Most people experience significant benefits from a nutritional ketosis state, such as weight loss, improved non-alcoholic fatty liver, better sleep, reduced blood sugar and lipid levels, among others. If possible, it is recommended to continue with the diet because it can lead to improved mental clarity, better sleep, increased endurance, reduced waist circumference, decreased body fat, stable blood sugar, and muscle growth. It is indeed an advantageous choice! Currently, there are ongoing intervention clinical studies evaluating the long-term safety and health outcomes, including sustainable treatment effects and economic impacts, of a low-carbohydrate ketogenic diet for individuals with type 2 diabetes and prediabetes. More research will provide insights into the safety of long-term adherence to this diet. An example that illustrates the persuasiveness of the diet is the case of a woman who has been following a low-carbohydrate ketogenic diet since May 2007, and as of July 2019, she is continuing with it, albeit with some intermittent periods. Although she did not strictly adhere to the diet, her sporadic use demonstrates that compared to other weight-loss diets, the low-carbohydrate ketogenic diet is still relatively easy to follow. At her first consultation, her initial weight was 342 lb (155.1 kg), and by March 2017, her weight had dropped to 264 lb (119.7 kg), with the most recent weight recorded as 281 lb (127.5 kg) in July 2019. It is currently established that a low-carbohydrate ketogenic diet is a paradigm-shifting dietary approach. If you ensure sufficient intake of minerals and antioxidants and do not experience adverse reactions or abnormalities in blood tests, then you can continue with the low-carbohydrate ketogenic diet indefinitely and maintain a state of ketosis. However, it is advisable to undergo a comprehensive health check-up before starting a low-carbohydrate ketogenic diet. During the implementation of the diet, it is important to be aware of any adverse reactions that may occur after the initial adaptation phase. If any nutrient deficiencies are detected, timely and appropriate supplementation is recommended. It is also advisable to undergo another comprehensive check-up after approximately two months to assess any changes compared to the initial baseline. If there are elevated triglycerides, decreased levels of beneficial cholesterol, or other abnormalities, it indicates a need for reevaluation. It may be necessary to reduce calorie intake, improve the quality of food sources (especially fatty acids), or consider adjusting the diet with the guidance of a healthcare professional or using nutritional supplements. Of course, if you feel tired of following a low-carbohydrate ketogenic diet or miss certain snacks, fruits, or pastries, you can adopt a more flexible low-carbohydrate diet. However, returning to a high-carbohydrate diet is generally not recommended, as it can lead to obesity, blood sugar issues, and cardiovascular problems. A high-carbohydrate diet is inherently incompatible with human genetics, particularly in the modern lifestyle context, and refined starches and sugary beverages, in particular, are the root cause of many diseases. All chronic illnesses are closely related to these modern high-carbohydrate diets. Therefore, it is advisable to adhere to a low-carbohydrate diet. | The safety of the low-carbohydrate ketogenic diet varies among different population groups, and there may be risks associated with its long-term use. Here are some details regarding the safety of the low-carbohydrate ketogenic diet: Long-term low-carbohydrate ketogenic diet may lead to nutrient deficiencies: Following this diet for an extended period can result in deficiencies of certain essential nutrients such as fiber, vitamin C, folate, etc. Long-term low-carbohydrate ketogenic diet may increase the risk of cardiovascular disease: Some studies suggest that prolonged adherence to a low-carbohydrate ketogenic diet may elevate the risk of cardiovascular diseases. Long-term low-carbohydrate ketogenic diet may impact kidney function: Continuously following a low-carbohydrate ketogenic diet for an extended duration may impose additional stress on the kidneys, potentially leading to renal abnormalities. Long-term low-carbohydrate ketogenic diet may result in ketoacidosis: Excessive production of ketone bodies in the body can lead to a condition called ketoacidosis, which is a severe metabolic disorder that can be life-threatening. Therefore, the low-carbohydrate ketogenic diet is not suitable for everyone and should be pursued under the guidance of a doctor or a nutritionist. If considering lifelong adherence to the low-carbohydrate ketogenic diet, it is important to regularly monitor one's health and ensure adequate intake of essential nutrients. If any adverse symptoms occur, it is recommended to discontinue the low-carbohydrate ketogenic diet immediately. |
| 9 | *What are the recommended meat dishes and condiments for the low-carbohydrate ketogenic diet?* | (1) Organic and grass-fed meats: Unprocessed meats are low-carbohydrate foods and beneficial for a nutritious ketogenic diet. Organic and grass-fed meats are the healthiest options. However, it's important to remember that the low-carbohydrate ketogenic diet is a high-fat diet rather than a high-protein diet. You don't need a large quantity of meat (as meat contains a decent amount of protein). Excessive protein intake beyond what your body needs can be converted to glucose, making it harder for your body to enter a state of nutritional ketosis. Pay attention to processed meats such as sausages and meatballs, as they often contain added carbohydrates like starch or sugar. If you're unsure about their ingredients, check the food labels and aim for foods with carbohydrate content below 5%. (2) Seafood: Seafood is an excellent source of healthy fats, such as salmon, shrimp, and shellfish. However, when processing seafood, try to avoid using thickening agents like starch, as they contain carbohydrates. (3) Eggs: Various types of eggs such as chicken eggs, duck eggs, and quail eggs can be cooked in different ways, such as steaming, boiling, frying, or scrambling. If possible, opt for fresh eggs from free-range chickens.  (4) Natural fats and high-fat sauces: The majority of calories in the ketogenic diet should come from fat. You can obtain sufficient fat from natural sources like meat, poultry, fish, and eggs. Add butter or coconut oil during cooking or incorporate generous amounts of olive oil in salads. Remember not to be afraid of fat; in the ketogenic diet, fat is your friend. However, we don't recommend condiments and canned foods that contain sugar. | The low-carbohydrate ketogenic diet promotes a high-fat, moderate-protein, low-carbohydrate eating structure. When selecting meat dishes and condiments, it's important to avoid consuming excessive carbohydrates. Here are some meat dishes and condiments suitable for the low-carbohydrate ketogenic diet: Meat dishes: Poultry: Chicken, turkey, duck, goose, quail, pigeon, etc. Livestock: Beef, lamb, pork, lean meats, etc. Fish: Salmon, trout, cod, bass, etc. Seafood: Shrimp, crab, shellfish, etc. Condiments: Olive oil, coconut oil, butter, fish oil, etc. Seasonings: Garlic, onion, ginger, chili, etc. Dairy products (in moderation): Yogurt, cheese, etc. Protein supplements: Protein powder, protein drinks, etc. It's important to note that different types of meat and seafood contain varying amounts of fat. Therefore, when choosing, consider your fat intake requirements. Additionally, dairy products like yogurt and cheese contain protein but also have a certain amount of carbohydrates, so it's essential to consume them in moderation. |
| 10 | *Does the low-carbohydrate ketogenic diet include vegetables and fruits?* | Certainly, it does. However, it's important to consume vegetables and fruits with high starch content in moderation. We recommend the following options: (1) Above-Ground Vegetables: It's best to choose green vegetables that grow above the ground, such as cauliflower, cabbage, broccoli, and zucchini. In the low-carbohydrate ketogenic diet, you can increase your fat intake while consuming vegetables, such as sautéing them in coconut oil or butter and adding plenty of olive oil to salads. Some even consider vegetables as a medium for fats, as they not only help increase fat intake but also add variety, flavors, and colors to the food. Since vegetables replace carbohydrate-rich root vegetables like rice, noodles, and potatoes, most people tend to consume more vegetables after starting the low-carbohydrate ketogenic diet. (2) Nuts: Nuts can be consumed in moderation, but caution should be exercised when using them as snacks, as it's easy to overeat. Cashews, in particular, have relatively higher carbohydrate content, so alternatives like macadamia nuts or walnuts can be chosen. (3) Berries: Most berries can be consumed in moderation on a low-carbohydrate ketogenic diet. | The low-carbohydrate ketogenic diet includes a small amount of vegetables and fruits, but the intake needs to be controlled to maintain a low-carbohydrate range of 20-50 grams per day. Some low-carbohydrate vegetables include spinach, cauliflower, asparagus, broccoli, tofu, pumpkin, mushrooms, and leafy greens. Low-sugar fruits include raspberries, blackberries, strawberries, blueberries, lemons, watermelon, oranges, and grapefruits. When selecting vegetables and fruits, it's advisable to choose low-sugar, low-starch, and high-fiber options. |
| 11 | *How should beverages and dairy products be selected in a low-carbohydrate ketogenic diet?* | (1) Beverages: Water is the preferred and best choice of beverage. You can enhance the flavor by adding lemon slices or natural flavorings. During the low-carbohydrate ketogenic diet, if you experience headaches or "flu-like" symptoms, you can add a small amount of salt to water. Unsweetened coffee or tea is also good. Ideally, avoid using sweeteners. To obtain additional energy from fats, you can add a small amount of coconut oil, butter, or cream to coffee or tea. Coffee with added butter, cream, or coconut oil and medium-chain triglycerides is known as "bulletproof coffee" and has become popular, but be cautious of the sugar content in latte coffee. You can drink tea as long as you don't add sugar, and most teas are allowed. Avoid consuming alcohol or sugary drinks. (2) High-Fat Dairy Products: Generally, the higher the fat content, the better, such as butter and high-fat cheese. Cream can also enhance the taste of cooking. For patients with conditions like diabetes or metabolic syndrome following a strict low-carbohydrate ketogenic diet, doctors may advise temporarily avoiding or consuming less milk because milk has a relatively high carbohydrate content. Additionally, be mindful that a small amount of milk can be added to coffee, but it's best to avoid lattes as they contain a significant amount of sugar and high carbohydrate content (a 350ml cup of latte coffee contains about 18g of carbohydrates). Avoid consuming low-fat yogurt as it often contains a significant amount of added sugar. Lastly, it is not advisable to regularly eat cheese when not feeling very hungry as it can affect the rate of weight loss. (3) Bone Broth: Bone broth is a good beverage option rich in nutrients and electrolytes. It is simple to prepare and can be enhanced with a little butter for extra energy. | In a low-carbohydrate ketogenic diet, beverages should be zero-calorie or low-calorie options, such as unsweetened drinks, tea, coffee, and water. Avoid consuming sugary beverages, fruit juices, sweet wines, beer, and other high-sugar drinks. If you enjoy milk, it is recommended to choose low-fat or skim milk. In a low-carbohydrate ketogenic diet, dairy products are generally considered good sources of protein and fat. Choose high-fat, low-carbohydrate dairy products such as cheese, butter, cream, and yogurt. Avoid sugary yogurt and low-fat dairy products as they often contain significant amounts of added sugar and starch. |
| 12 | *What foods should be avoided in a low-carbohydrate ketogenic diet?* | (1) Sugar: This is a major taboo, and all sugary drinks should be eliminated, including juices, sports drinks, and "vitamin water" (which are essentially sugary water). Avoid candies, cakes, cookies, chocolates, doughnuts, frozen snacks, and breakfast cereals (Figure 2-2-14). Learn to read food labels for hidden sugars, especially in sauces, condiments, beverages, seasonings, and pre-packaged foods. Honey and agave nectar also contain sugar, so it's best to avoid or limit the intake of artificial sweeteners. (2) Starch: This includes foods such as bread, noodles, pasta, rice, rice noodles, potatoes, sweet potatoes, french fries, potato chips, porridge, and oatmeal (Figure 2-2-15). Legumes like soybeans and lentils also have high carbohydrate content. Some starchy root vegetables or fruits (such as potatoes, yams, pumpkins, etc.) have relatively high starch content. (3) Beer: Beer is often referred to as liquid bread due to its high carbohydrate content that can be rapidly absorbed. While a few beers may have lower carbohydrate content (Figure 2-2-17), most beers should be avoided. (4) Fruit: Fruits are generally sweet and can be considered a natural form of candy due to their high sugar content. They can be occasionally consumed, but severely overweight individuals and those with metabolic diseases should try to avoid fruits (Figure 2-2-18). (5) Margarine: Margarine is an artificial butter substitute commonly produced in the industry. It contains high levels of 0-6 fatty acids that are detrimental to health and has an unpleasant taste. Margarine has been associated with asthma, allergies, and other inflammatory conditions. | The goal of a low-carbohydrate ketogenic diet is to reduce carbohydrate intake while increasing protein and healthy fat intake. Here are the foods that should be avoided or limited: Sugars and sugary foods, such as candies, chocolates, preserves, cakes, cookies, ice cream, etc. Starchy foods, including bread, rice, noodles, potatoes, corn, taro, sweet potatoes, etc. Fruits because they have relatively high carbohydrate content, such as bananas, oranges, apples, pears, etc. Grains and grain products, like oats, cereals, wheat, cornflakes, etc. Legumes and legume products, such as tofu, soy milk, soybeans, black beans, green beans, etc. Low-fat dairy products as they often contain higher lactose content, such as milk, yogurt, cheese, etc. Red meat and processed meat products, like pork, beef, lamb, sausages, bacon, etc. In a low-carbohydrate ketogenic diet, beer is generally considered unsuitable due to its high carbohydrate and sugar content. A typical beer can contain over 10 grams of carbohydrates, and certain specialty beers may have even higher carbohydrate and sugar content. Therefore, it's best to avoid beer as a high-carbohydrate beverage in this diet. If you wish to consume alcohol, you can opt for low-carbohydrate options like dry red wine or spirits. It's important to note that while these foods should be restricted, they don't necessarily need to be completely avoided. The needs and limitations can vary from person to person in a low-carbohydrate ketogenic diet, so it's advisable to consult a doctor or nutritionist for personalized advice. |
| 13 | *What should be noted when purchasing, using, and cooking food for a low-carbohydrate ketogenic diet?* | (1) Avoid adding sugar, thickeners, or coating with flour when cooking. It is recommended to have most meals at home. (2) When buying packaged foods, learn to read the "ingredients list" and look for products that do not contain white sugar, honey, and artificial sweeteners (such as aspartame, saccharin, acesulfame, and sucralose). (3) When purchasing food, carefully read the food labels and look for products with a carbohydrate content of ≤5g per 100g. Recently, low-carbohydrate ketogenic diets have become very popular, and many food companies add labels like "low-carb" to their new products to make money. However, we need to be cautious about these "low-carb" products. Foods such as pasta, chocolate bars, energy bars, protein powders, snacks, cakes, cookies, and other foods or snacks labeled as "low-carb" are not necessarily true low-carbohydrate foods. Therefore, when purchasing food, it is important to read all labels carefully and understand which ones are truly low in carbohydrates, with fewer additional ingredients being preferable. Beware of deceptive marketing tactics that hide the actual carbohydrate content. (4) If strict adherence to a low-carbohydrate ketogenic diet is desired, it is best to also limit or avoid dairy products (milk, cheese, yogurt). If you are not accustomed to eating nuts, it is recommended not to start consuming them as excessive nut consumption can lead to an excessive intake of carbohydrates. (5) It is preferable to focus on consuming high-quality natural foods or minimally processed foods. | Choose high-quality protein foods: Select foods rich in high-quality protein, such as meat, fish, poultry, shrimp, and crab, to ensure an adequate protein intake. Choose healthy fat foods: Select foods rich in healthy fats, such as fish oil, olive oil, flaxseed oil, and coconut oil, and avoid consuming unhealthy fats, such as trans fats. Choose low-carbohydrate vegetables: Choose vegetables that are high in dietary fiber and low in carbohydrates, such as spinach, cauliflower, broccoli, asparagus, onions, and cucumbers. Avoid processed foods: Try to avoid purchasing processed foods, such as canned foods, fast food, deli meats, and cakes, as they often contain high amounts of carbohydrates and additives. Control portion sizes: A low-carbohydrate ketogenic diet does not mean you can freely consume high-fat and high-protein foods, so it is important to pay attention to portion control. Cooking methods: Opt for lighter cooking methods, such as steaming, boiling, and stewing, and avoid excessive grilling, frying, and deep-frying. Most importantly, if you plan to start a low-carbohydrate ketogenic diet, it is recommended to consult with a doctor or nutritionist for advice to ensure that your dietary plan is safe, healthy, and scientifically sound. |
| 14 | *How to understand nutrition labels when shopping at supermarkets?* | When shopping at supermarkets, the first thing to pay attention to is the freshness of the ingredients. For fish, meat, poultry, and eggs, check the production date and expiration date. When faced with various packaged foods to purchase, it is essential to learn how to read nutrition labels to determine what to buy and what not to buy, especially when following a low-carbohydrate ketogenic diet. There are mainly two types of nutrition labels.  "Nutrition Facts": This is a standardized table that includes the names, amounts, and percentages of the reference nutrient intake (RNI) of the food's nutritional components. It is a required part of nutrition labels and provides information about the nutrients that the body can obtain from consuming the food, including energy, protein, fat, carbohydrates, sodium (the component of salt), etc. Generally, the values are given per 100g and/or per 100ml and/or per serving. "Ingredients List": This lists the components and composition of the food, following the principle of "ingredients listed in descending order of weight." Many foods do not specifically list the amount of sugar, but the carbohydrate content can indicate the level of sugar content. The main categories of ingredients are described as follows: Sugars: Various added sugars are labeled as white sugar, sucrose, fructose, icing sugar, glucose, maple syrup, maltose, powdered sugar, fruit glucose syrup, malt syrup, high fructose syrup, honey, floral honey, etc. Foods that are rich in added sugars include beverages, candies, bread, cookies, hamburgers, cakes, ice cream, etc. Grains: Be cautious of starchy carbohydrate-rich grain and potato-based foods. Grains include wheat flour, rice, corn, sorghum, etc., and their products such as rice, steamed buns, pancakes, corn tortillas, bread, cookies, cereals, etc. Tubers include sweet potatoes (also known as yams), potatoes, cassava (also known as tapioca), and taro. Cooking oil and dairy: Choose oils such as coconut oil, olive oil, palm oil, lard, perilla oil, rapeseed oil, etc. Properly select seasonings, dried goods, dairy products, and other processed foods based on the nutrition facts. For example, even for milk products, the sugar content can vary significantly. When choosing milk in the market, try to select products with lower carbohydrate content per 100ml. Regular milk contains lactose, usually around 5%. However, some flavored milk and yogurts may have additional sugar added for taste improvement, so it is best to avoid milk with added sugar or condensed milk as ingredients. It is important to understand nutrition labels when shopping at supermarkets. By reading the labels, you can make informed choices about what to buy and ensure that the food aligns with your dietary requirements, especially when following a low-carbohydrate ketogenic diet. | Food Composition Table: The food composition table on the nutrition label should list the nutritional components per serving, including calories, carbohydrates, fats, proteins, fibers, sodium, etc. Pay attention to the serving size to accurately calculate your daily intake. Daily Nutritional Requirements: Nutrition labels often provide the percentage of daily nutritional requirements to help you understand the contribution of each food item towards meeting your daily nutritional needs. Note that daily nutritional requirements may vary from person to person, so you need to calculate the percentages based on your specific circumstances. Additives: Some foods may contain chemical substances such as artificial colors, preservatives, thickeners, etc., which may have an impact on human health. When choosing foods, try to avoid those with high levels of additives. Key Nutrients: Pay particular attention to key nutrients such as calories, fats, proteins, carbohydrates, and sodium, as these components can have a significant impact on your health and require special attention. In addition, you can consider the following suggestions: Compare different brands and types of food to choose the healthiest options. If you need to control the intake of certain nutrients, such as sugar or sodium, pay attention to the content of these components in the food composition table. Opt for natural and fresh foods rather than processed ones, as processed foods often contain more additives and sugars. If you have specific dietary requirements or health issues, consult a doctor or nutritionist for advice. |
| 15 | *How can you tell if you have entered a nutritional ketosis state?* | How can you determine if you are in a nutritional ketosis state? It can be judged based on certain symptoms such as dry mouth, increased urination, fruity-smelling breath, reduced hunger, increased energy, etc. However, a more accurate assessment can be made through urine, blood, or breath gas testing. Figure 2-2-24 shows the changes in blood ketone levels when using blood ketones as a reference. Let's explain the blood ketone level variations seen in Figure 2-2-24: (1) Dark green blood ketone level zone: Blood ketone concentration of 1.5-3 mmol/L is considered the optimal nutritional ketosis state. At this point, both mental and physical states are at their best. This is also the state where fat burning is the fastest, leading to sustained weight loss. (2) Light green blood ketone level zone: Blood ketone concentration of 0.5-1.5 mmol/L is classified as mild nutritional ketosis. This helps with weight loss but is not the optimal state. (3) Gray blood ketone level zone: Blood ketone concentration ≤0.5 mmol/L cannot be considered a nutritional ketosis state. Although some suggest that a value of 0.2 mmol/L is close to nutritional ketosis, this level is far from maximizing fat burning. (4) Yellow blood ketone level zone: Blood ketone concentration >3 mmol/L is actually unnecessary, but it does indicate a higher ketosis state. However, excessively high blood ketone levels may sometimes indicate insufficient food intake (starvation ketosis). In the case of type 1 diabetes, it may be due to severe insulin deficiency, requiring urgent attention and treatment. (5) Orange-red blood ketone level zone: Blood ketone concentration >8 mmol/L is generally not achievable with a simple low-carbohydrate ketogenic diet. This indicates a pathological state. Currently, this condition is mainly seen in type 1 diabetes with severe insulin deficiency. Common symptoms include nausea, vomiting, abdominal pain, and confusion. This can lead to diabetic ketoacidosis, even life-threatening, and requires urgent rescue treatment. | To determine if you have entered a nutritional ketosis state, there are several methods you can choose from: Measuring ketone levels: Use urine test strips or blood ketone meters to measure your ketone levels. In a nutritional ketosis diet, ketone levels typically range between 0.5 to 3.0 mmol/L, although actual values may vary among individuals. Observing physical signs: After entering a nutritional ketosis state, your body may exhibit characteristic symptoms such as dry mouth, bad breath, increased urination, and fatigue. Conversely, you may also experience positive signs such as increased energy and mental clarity. Monitoring food intake: In a nutritional ketosis diet, carbohydrate intake is usually restricted to around 5-10% of total daily calories, while fat and protein make up approximately 70% and 20% of total calories, respectively. If you can accurately control the intake of these nutrients and observe changes in your ketone levels, you can infer whether you have entered a nutritional ketosis state. Please note that everyone's body condition and metabolic rate are different, so the time and process required to enter a state of ketosis may vary. If you feel uncomfortable while attempting to enter ketosis, please stop and consult a healthcare professional for advice. It's also important to maintain a balanced and diverse diet, not overly focusing on a nutritional ketosis diet while neglecting other nutritional needs of your body. |
| 16 | *How long does it take to enter a state of nutritional ketosis with a low-carbohydrate ketogenic diet? What happens if you consume more carbohydrates?* | At the Low-Carb Medical Clinic in Bao'an District Central Hospital in Shenzhen, we have observed that patients reach a blood ketone level of 0.5mmol/L or higher after strictly following a low-carbohydrate ketogenic diet for one week. Blood ketone testing can monitor and assess whether a patient's metabolism has changed in response to the low-carbohydrate ketogenic diet, indicating compliance and the body's state of burning fat (producing ketones). When your body has entered a good state of nutritional ketosis and has adapted to this state (keto-adaptation), it means that your body is using fats and ketones as fuel, and blood ketone levels will be maintained at 0.5mmol/L or higher. When in this state, if you consume carbohydrates ≥75g, your blood sugar will immediately rise, insulin will be secreted by the body, fat burning will stop, and the fat will be locked in the cells. Even if you continue with a strict low-carbohydrate diet afterward, it may take up to two weeks for your body to regain the ketone-adapted state you had before. Therefore, if you want to continue burning body fat and reap the benefits of a low-carbohydrate ketogenic diet, you cannot cheat or exceed the carbohydrate intake limits during the diet. If you do so, fat burning will immediately cease, and it will take a considerable amount of time to restore the fat-burning state. If you want to reenter and adapt to that nutritional ketosis state, you may also experience adverse reactions that occurred when you initially adopted the strict low-carbohydrate ketogenic diet due to the transition in energy metabolism. | The time it takes to enter a state of nutritional ketosis can vary from person to person and depends on individual factors such as body condition, dietary composition, level of physical activity, and other factors. Typically, it takes 2-7 days to enter a state of nutritional ketosis, but in some cases, it may take longer. During the diet, you can assess whether you have entered a state of nutritional ketosis by measuring the levels of ketones in your blood. If you consume too many carbohydrates on a low-carbohydrate ketogenic diet, it can potentially take your body out of the state of nutritional ketosis. Excessive carbohydrate intake stimulates insulin secretion, which lowers the levels of ketones in the blood. If this happens, it is generally recommended to reduce carbohydrate intake to re-enter the state of nutritional ketosis. It is important to note that both excessively low and high carbohydrate intake can have negative effects on the body. Therefore, when following a low-carbohydrate ketogenic diet, it is important to choose an appropriate amount of carbohydrates based on individual circumstances and health conditions. Additionally, it is crucial to pay attention to the quality and types of foods consumed to ensure an adequate intake of essential nutrients. |
| 17 | *I heard that the low-carbohydrate ketogenic diet can have some potential adverse reactions. What are they, and is there a way to deal with them?* | When you suddenly switch your body's energy source from burning carbohydrates (glucose) to burning fat, there may be some adverse reactions. The common adverse reactions include flu-like symptoms, leg cramps, constipation, bad breath, palpitations, and reduced exercise performance. Less common adverse reactions include potential risks during breastfeeding, gallstones, temporary hair loss, increased cholesterol, decreased alcohol tolerance, rash, gout, and increased fasting blood sugar. If flu-like symptoms (headaches, fatigue, etc.), cramps, and palpitations are mild and short-lived, you can reduce or even eliminate these symptoms by increasing your intake of water and salt. One simple method is to drink meat broth 1-2 times a day. Additionally, you can supplement with some multivitamins. For the majority of people, these adverse reactions will quickly disappear. | While the low-carbohydrate ketogenic diet has many benefits, it also has some potential adverse reactions. Here are some common ones: Nutrient deficiencies: Restricting carbohydrate-rich and fiber-rich foods such as fruits, vegetables, and whole grains in a low-carbohydrate ketogenic diet can lead to deficiencies in fiber, vitamins, minerals, and other nutrients, resulting in symptoms such as constipation, diarrhea, and fatigue. Ketoacidosis: If the intake of fats is too high and the body cannot metabolize them into ketones in a timely manner, it can result in excessively high levels of ketones in the blood, leading to ketoacidosis. Symptoms include deep and rapid breathing, nausea, vomiting, abdominal pain, and impaired consciousness. In severe cases, it can be life-threatening. Dehydration: In a low-carbohydrate ketogenic diet, as the body metabolizes fats to produce ketones, it requires water for ketone metabolism. This can potentially lead to dehydration, with symptoms such as dry mouth, dizziness, and fatigue. Selective eating: Due to the restrictions of a low-carbohydrate ketogenic diet, individuals may end up consuming a high-fat, high-cholesterol, and low-fiber diet, which can increase the risk of chronic diseases such as cardiovascular disease, hypertension, and diabetes. To avoid these adverse reactions, it is important to choose high-quality sources of fats and proteins, ensure an adequate intake of fiber and other nutrients, maintain sufficient hydration, and consider individual circumstances and health conditions when deciding whether to adopt a low-carbohydrate ketogenic diet. It is also crucial to follow the guidance of healthcare professionals or experts in the field. |
| 18 | *Why do flu-like symptoms occur when adopting a low-carbohydrate ketogenic diet, and how to cope with them?* | After adopting a low-carbohydrate ketogenic diet, most people will experience flu-like symptoms, especially in the first week, particularly within the first 2-5 days. These symptoms mainly include headaches, drowsiness, nausea, brain fog, and irritability. The most common symptom during this transition period is headaches, often accompanied by fatigue, drowsiness, and lethargy. Nausea may also be experienced, along with a sense of cognitive fuzziness or brain fog. Additionally, becoming easily irritable is a noticeable feeling reported by family members. Fortunately, these symptoms will usually resolve on their own within a few days. The main cause of flu-like symptoms is dehydration due to increased urine output and/or salt deficiency. The following methods can help address these symptoms: Increase water and salt intake: Adequate hydration and moderate salt intake can effectively alleviate these adverse reactions, and some symptoms may even disappear quickly. You can add 0.2g of salt to a glass of water (200-250ml) and consume it. Symptoms should alleviate within 15-30 minutes. If you experience discomfort during the first week of the low-carbohydrate ketogenic diet, you can drink a glass of saltwater every day. Alternatively, you can choose flavorful meat broths like chicken, beef, or bone broth. If you have hypertension or other cardiovascular issues that require restricted salt and water intake, do not use salt, and limit salt intake to a maximum of 2g per day, with a water intake of no more than 2 liters per day. Additionally, ensure sufficient rest and consider taking pain relievers to alleviate headaches and body pain. Consume an adequate amount of fat: Insufficient fat intake while on a low-carbohydrate diet can lead to intense hunger and increased fatigue. Some individuals may struggle with adapting to the feeling of hunger. A proper low-carbohydrate ketogenic diet should include an ample amount of fat to provide a good sense of satiety, energy, and to shorten the adaptation period, reducing feelings of low energy. To ensure sufficient fat intake during a low-carbohydrate ketogenic diet, you can add fats like cooking oils and butter to your meals. If the flu-like symptoms persist even after consuming saltwater, the best approach is to temporarily ignore them. As your body adapts to the low-carbohydrate ketogenic diet and begins to utilize fats for energy, all symptoms should gradually disappear within a few days. However, if necessary, you can also consume a small amount of carbohydrates to make the transition period gentler. Keep in mind that this should not be the primary approach, as it can prolong the transition phase and slow down weight loss and the extent of health improvements. If symptoms are severe or prolonged, it is advisable to consult a healthcare professional for guidance. | During the adoption of a low-carb-ketogenic diet, the so-called "ketogenic flu" or "low-carb reaction" may occur. This is due to a decrease in blood sugar and insulin levels as the body adjusts to the new eating pattern, as well as changes in the body's water and electrolyte balance, resulting in a number of discomforting symptoms. These symptoms include headache, dizziness, fatigue, constipation, thirst, nausea and diarrhoea, similar to the symptoms of a cold or flu, hence the name 'ketogenic flu'. The following measures can be taken to combat these discomforts: Get enough water and electrolytes, such as sodium, potassium and magnesium, by drinking plenty of water or low-carbohydrate drinks, adding salt or eating foods containing potassium and magnesium. Moderate increase in carbohydrate intake, e.g. increased intake of vegetables, nuts, seeds and berry fruits to ease discomfort. Gradually transition to a low-carbohydrate - ketogenic diet and avoid changing eating patterns too quickly. If symptoms are severe or prolonged, consult your doctor for advice. |
| 19 | *How to deal with leg cramps, constipation, and bad breath when following a low-carbohydrate ketogenic diet?* | Experiencing these symptoms often indicates that after a period of low-carbohydrate and ketogenic diet, your body has entered a good state of nutritional ketosis, and rapid fat loss is underway. (1) Leg cramps: Leg cramps are quite common during the initial phase of a low-carbohydrate and ketogenic diet and can cause pain. This is mainly due to increased urine output leading to mineral loss, especially magnesium ions. How can you avoid leg cramps? There are three methods: 1) Drink an adequate amount of water and consume necessary salt to reduce the loss of magnesium ions and prevent leg cramps. 2) If necessary, supplement magnesium ions. For the first 20 days, you can take three slow-release magnesium tablets daily, and later reduce it to one tablet per day. 3) If the above methods are ineffective, you can consider increasing your carbohydrate intake to alleviate leg cramps, but the more carbohydrates you consume, the less effective it will be for improving health. (2) Constipation: During the initial phase of a low-carbohydrate and ketogenic diet, the digestive system is in a transitional period, and constipation may occur. There are five methods to prevent constipation: 1) Drink an adequate amount of water and consume necessary salt. Dehydration is the main cause of constipation. Increased water absorption in the colon leads to drier and harder stools. Therefore, it is recommended to drink water and supplement an appropriate amount of salt. 2) Eat more vegetables or supplement dietary fiber from other sources. Adequate intake of high-quality dietary fiber can promote intestinal motility and reduce the risk of constipation. A low-carbohydrate and ketogenic diet may limit the sources of certain dietary fibers, leading to constipation. Increasing the intake of non-starchy vegetables can help solve this problem. 3) If the above suggestions are still ineffective, you can take milk of magnesia, which contains magnesium hydroxide, to relieve constipation. 4) If necessary, you can slightly increase your carbohydrate intake. 5) Avoid eating too many nuts and develop a habit of regular bowel movements. Drinking cold saltwater before bowel movements can promote intestinal motility and facilitate defecation. (3) Bad breath: Exhaling with a smell resembling nail polish remover indicates the presence of acetone, which signifies that your body is burning a significant amount of fat and converting it into ketones to provide energy to the brain. Sometimes the body may emit this odor, especially after exercising and sweating heavily. Not everyone on a low-carbohydrate and ketogenic diet will experience this bad breath. For most people, the bad breath disappears after 1 to 2 weeks as the body gradually adapts and stops eliminating ketones through breath and sweat. However, for some individuals, the bad breath may persist. Here are some suggestions for these individuals: 1) Ensure an adequate diet and supplement a suitable amount of salt. If you experience dry mouth, it indicates a decrease in saliva that helps wash away bacteria, leading to bad breath. Therefore, it is important to ensure sufficient water intake. 2) Maintain oral hygiene. Brushing your teeth twice a day cannot guarantee the disappearance of ketone odor since it originates from the lungs, but oral cleanliness ensures that no additional flavors mix in. 3) Use breath fresheners regularly to mask the ketone odor. If you have been dealing with chronic bad breath and want to get rid of it, a simple solution is to reduce the intensity of the low-carbohydrate and ketogenic diet. This means slightly increasing your carbohydrate intake to around 50-70g per day. However, keep in mind that by doing so, the effectiveness of weight loss and alleviating conditions like diabetes may be compromised. Nevertheless, this approach may still work for some individuals.4) Combine it with intermittent fasting. By consuming 50-70g of carbohydrates per day and incorporating appropriate intermittent fasting, the effects can be nearly equivalent to a low-carbohydrate and ketogenic diet, without the bad breath.5) The first two measures mentioned above are more general, while the last three are specifically targeted at addressing the bad breath caused by ketosis. After implementing these methods, it is advisable to observe for 1-2 weeks and see if the bad breath disappears or improves. In most cases, bad breath is temporary and will resolve with these adjustments. | During the process of adopting a low-carbohydrate ketogenic diet, there may be some adverse reactions such as leg cramps, constipation, and bad breath. Here are some methods to deal with them: Leg cramps: This could be due to the loss of certain electrolytes, especially sodium, magnesium, and potassium. It can be relieved by increasing the intake of foods that contain these electrolytes, such as codfish, spinach, almonds, sesame seeds, broccoli, etc. Constipation: Adopting a low-carbohydrate ketogenic diet may reduce the intake of dietary fiber, which can lead to constipation. Therefore, it is important to increase the intake of high-fiber vegetables and fruits, such as spinach, asparagus, broccoli, berries, etc. Bad breath: Following a low-carbohydrate ketogenic diet may cause bad breath, which could be attributed to the volatile compounds produced by oral bacteria breaking down protein and fat. It is recommended to brush teeth more frequently, use sugar-free gum, or use mouthwash containing chlorine. It is important to note that if these adverse reactions persist for a prolonged period or if the symptoms are severe, it is advisable to seek medical advice. |
| 20 | *What should I do if I feel palpitations after adopting a low-carbohydrate ketogenic diet?* | During the initial phase of adopting a low-carbohydrate ketogenic diet, it is common to experience a slight increase in heart rate or heightened heart palpitations, often feeling uneasy or palpitations. This is normal and there is no need to worry excessively. The main reason is dehydration and electrolyte deficiency, which leads to a decrease in blood volume in the body. In such cases, the heart compensates by increasing its rhythm or intensity to maintain normal blood pressure. A quick solution is to drink an adequate amount of water and consume sufficient salt. If increasing water and salt intake does not completely eliminate palpitations, then the palpitations may also involve increased regulation of hormone secretion in the central nervous system, often aimed at maintaining blood sugar levels, especially when you are taking hypoglycemic medication. However, these palpitations are also temporary, and after 1-2 weeks, when the body adapts to the low-carbohydrate ketogenic diet, the symptoms will disappear. In special cases, some individuals may experience persistent palpitations. If this becomes bothersome, it is advisable to increase carbohydrate intake to a suitable level. Of course, this will result in a reduction in the effectiveness of improving health, and it is necessary to find a balance for oneself. There are several points to note for individuals with diabetes or hypertension who are currently on medication: (1) Diabetes: Adopting a low-carbohydrate ketogenic diet helps to avoid the consumption of carbohydrates that can cause a rise in blood sugar, resulting in a corresponding decrease in the need for hypoglycemic medications. If you are still using the same dosage of insulin as before, it may lead to symptoms of hypoglycemia, one of which is palpitations. Therefore, it is important to monitor blood sugar levels frequently and adjust medication promptly. It is recommended to do this under the guidance of an internist. If you are a diabetic patient who can be controlled solely through diet and/or metformin, there is no need to worry about this point. (2) Hypertension: After adopting a low-carbohydrate ketogenic diet, the condition of hypertension tends to improve, resulting in a reduced need for medication. If you continue to take the same dosage of medication as before, it may lead to low blood pressure. One of the manifestations is an accelerated heart rate and palpitations. If hypertension patients experience these symptoms, it is important to regularly measure blood pressure. If it is below 110/70 mmHg, please consult with your managing physician to discuss how to reduce or discontinue medication. | Feeling palpitations may be due to an electrolyte deficiency in a low-carbohydrate ketogenic diet, such as sodium, magnesium, and potassium. Here are some methods to cope with palpitations: Increase electrolyte intake: You can alleviate palpitation symptoms by increasing the intake of electrolytes such as sodium, magnesium, and potassium. This can be achieved by consuming foods rich in these elements or by taking electrolyte supplements. Reduce exercise intensity: Excessive exercise can cause the body to lose water and electrolytes, exacerbating palpitation symptoms. Therefore, it is advisable to reduce exercise intensity appropriately. Increase water intake: In a low-carbohydrate ketogenic diet, ketones produced from the breakdown of body fat need to be excreted through urine, so it is important to increase water intake. If the symptoms are severe or persistent, consult a doctor: If palpitation symptoms persist or worsen, it is important to consult a doctor to rule out other potential health issues. |
| 21 | *Why does exercise performance decrease in a low-carbohydrate ketogenic diet, and how to cope with it?* | During the initial weeks of adopting a low-carbohydrate ketogenic diet, you may experience a noticeable decrease in exercise performance. This is primarily due to two reasons. Firstly, the body may lack fluids and salt. This is the main reason for the decline in exercise performance during the adaptation phase of a low-carbohydrate ketogenic diet, especially when the activity level increases. To cope with this, it is recommended to drink a large glass of saltwater (approximately 2.5g of salt) 30 to 60 minutes before exercise, as this can significantly improve your performance. Secondly, it is a transitional effect during the adaptation period. Adopting a low-carbohydrate ketogenic diet typically requires several weeks for the body to shift from relying on glucose as its primary fuel source to prioritizing fat for energy. This transition applies to muscle tissue as well, and it can take anywhere from a few weeks to several months. However, during this low-carbohydrate, high-fat dietary pattern, it is still advisable to maintain your exercise routine, as the more you exercise, the shorter the adaptation period will be, and you will reap greater benefits. Overall, while exercise performance may temporarily decrease after starting a low-carbohydrate ketogenic diet, it tends to improve in the long run. Many professional athletes in our country follow a low-carbohydrate, high-fat diet, and they have achieved significant improvements in their performance, particularly in endurance and long-distance running. Unlike muscle glycogen, which has limited storage capacity, the body has a substantial amount of stored fat. Once athletes adapt to utilizing fat for fuel, they can engage in prolonged exercise without the need for additional energy supplementation or with much lower fat consumption compared to others. This not only reduces the risk of digestive system issues but also allows for ample blood flow to the muscles, providing oxygen and energy. Ultimately, adopting a low-carbohydrate ketogenic diet can help reduce body fat percentage, lighten the body's burden, and improve exercise performance. | In a low-carbohydrate ketogenic diet, the body's glycogen stores are depleted, and glycogen is one of the primary energy sources for muscles. Therefore, during high-intensity exercise, you may experience fatigue and a lack of endurance. Additionally, due to the lower intake of protein in a ketogenic diet, it may lead to a decrease in muscle mass. To avoid a decrease in exercise performance, it is recommended to increase protein intake appropriately while following a low-carbohydrate ketogenic diet to maintain muscle mass. It is also advised to choose suitable exercise types and intensity levels, gradually adapting to the energy changes brought by the diet, and avoiding starting with overly intense exercise. Sufficient sleep and rest are also important to help the body recover and repair muscles. |
| 22 | *What should I do if temporary hair loss occurs after adopting a low-carbohydrate ketogenic diet?* | There are many reasons why temporary hair loss may occur after adopting a low-carbohydrate ketogenic diet, including sudden dietary changes. Temporary hair loss is especially common when using strict low-carbohydrate ketogenic diets (such as fasting or meal replacements), but it can also occasionally happen with moderate or liberal low-carbohydrate diets. Temporary hair loss typically occurs 3 to 6 months after dietary changes and is most noticeable when combing the hair. However, this condition is generally temporary, and only a small portion of hair will shed, which may not be very noticeable. After a few months, new hair follicles will grow, and the hair volume will return to its previous level. For longer hair, the recovery time may be longer, taking up to a year or more. If you experience temporary hair loss, there is no need to worry. Let's first understand how hair grows. Each hair on your head has a growth cycle of 2 to 3 years, after which it automatically stops growing for 3 months because new hair will grow from the same follicle, replacing the old hair. This is a common phenomenon. You shed some hair every day, but not all hair sheds synchronously, so you may not notice it. Because old hair sheds while new hair grows, your hair volume remains constant. So why does temporary hair loss occur after adopting a low-carbohydrate ketogenic diet, and how should you deal with it? If you are currently under significant psychological stress, hair loss may be more pronounced. There are various other reasons that can cause hair loss, such as starvation (including energy restriction and meal replacements), illness, intense exercise, pregnancy, breastfeeding, nutritional deficiencies, and sudden significant dietary changes. These factors can disrupt the hair growth cycle. If hair loss is due to childbirth or the transition from a regular diet to a low-carbohydrate ketogenic diet, you don't need to take any specific measures because this type of hair loss is temporary. Once hair loss is triggered, it will continue regardless of any measures taken, and stopping the low-carbohydrate ketogenic diet will not accelerate hair regrowth. Some people may consider getting a blood test to check for nutritional deficiencies, which is particularly relevant for individuals following vegetarian or vegan diets who may not be adequately supplementing vitamins B1 and B2. However, for others, this test may not have much practical significance. Hair loss is relatively rare among low-carbohydrate ketogenic diet followers, and most people do not experience this issue. Currently, there is no research available on how to reduce the risk of hair loss for individuals following this diet. It is important for individuals adopting a low-carbohydrate diet to ensure they do not excessively restrict nutrient intake and to prioritize sufficient fat consumption. This means that while reducing carbohydrate intake, it is essential to ensure an adequate intake of fats to avoid being in a state of starvation. Additionally, during the first week of adopting a low-carbohydrate ketogenic diet, it is especially important to manage stress, ensure good sleep quality, and avoid participating in intense physical activities until a few weeks later. | After adopting a low-carbohydrate ketogenic diet, temporary hair loss may occur due to deficiencies in certain nutrients such as vitamin B, vitamin C, folate, iron, and zinc. These nutrients play an important role in hair health. Therefore, you can consider increasing the intake of these nutrients in your diet or using relevant nutritional supplements to supplement them.  In addition, maintaining adequate hydration, keeping the scalp clean, and avoiding excessive pulling of the hair can also help reduce hair loss. If your hair loss symptoms persist or worsen, it is advisable to consult a doctor or nutritionist to determine the most appropriate treatment for you. |
| 23 | *How to understand and deal with the issue of increased cholesterol in a low-carbohydrate ketogenic diet?* | In general, a low-carbohydrate ketogenic diet has a regulating effect on cholesterol and can reduce the risk of heart disease. However, unlike cholesterol-lowering drugs, it primarily leads to a mild increase in cholesterol levels, with a focus on benign cholesterol—high-density lipoprotein cholesterol (HDL-C) elevation. At the same time, it also lowers triglycerides, resulting in a relatively loose structure of low-density lipoprotein cholesterol (LDL-C) that is less prone to deposition in the blood vessel walls, thus reducing the risk of coronary artery plaque formation. Research suggests that adhering to a low-carbohydrate ketogenic diet for 2 years can improve indicators related to coronary atherosclerosis. However, the following three points should be noted: Concerning potential risks: A low-carbohydrate ketogenic diet is not a lipid-lowering medication, and it cannot completely eliminate the potential risks of cholesterol. Although total cholesterol and LDL-C only show a slight increase, 1% to 2% of individuals still need to approach this issue with caution. For example, a small portion of the population may have genetic issues related to high cholesterol. If total cholesterol exceeds 400 mg/dL (10.34 mmol/L) and LDL-C exceeds 250 mg/dL (6.46 mmol/L) after starting this diet, even if HDL-C increases and triglycerides decrease, it is still considered abnormal. Regular cholesterol monitoring is necessary for these individuals. If the monitoring results show elevated LDL-C, an increased ratio of apolipoprotein B to apolipoprotein A1, indicating an increased risk of heart disease, consultation with a physician and the use of medication may be required. Currently, there is limited research on this particular situation, and further exploration and proposed solutions for mitigating these potential risks are anticipated. How to address this issue: If you experience abnormal lipid profiles after starting a low-carbohydrate ketogenic diet, it is important to pay attention to the following points. Avoid consuming "bulletproof coffee" or drinks (referring to coffee or beverages containing added coconut oil, cream, butter, and medium-chain fatty acids) when not feeling hungry, as consuming a large amount of fat during this time can effectively lower cholesterol levels. Eat when feeling hungry and incorporate intermittent fasting, as this can also lower cholesterol levels. Consume more unsaturated fatty acids such as olive oil, fish oil, and avocado. Although it is uncertain whether this approach can improve health, it certainly can lower cholesterol levels. If these three methods are still ineffective, it may be necessary to reconsider your overall health status and determine if a low-carbohydrate ketogenic diet is truly suitable for you. If a moderate low-carbohydrate diet (50-100g of carbohydrates per day) still proves effective for you, you can consider slightly increasing carbohydrate intake to lower cholesterol levels. It is important to choose high-quality carbohydrates rather than refined flour or sugars. Regarding the use of statin medications: Some may suggest that instead of going through the effort of following a low-carbohydrate ketogenic diet, it would be easier to simply take statin medications. However, we must emphasize that the clinical use of statin cholesterol-lowering drugs has always been controversial. While these medications can indeed lower the risk of heart disease, they also increase the risk of liver damage, mental fatigue, muscle soreness, type 2 diabetes, and slight decreases in intelligence quotient (IQ). Current relevant guidelines indicate that taking statin medications for 5 years can effectively reduce the risk of heart disease by 1% and extend life by 3 days if you have a risk of heart disease. | Low-carbohydrate ketogenic diets may lead to changes in blood lipids, including an increase in cholesterol. This is because the intake of fats in the diet increases, and the intake of protein may also increase. This can potentially result in elevated levels of low-density lipoprotein cholesterol (LDL-C). However, this does not necessarily mean that a low-carbohydrate ketogenic diet increases the risk of cardiovascular disease. In fact, some studies suggest that during a low-carbohydrate ketogenic diet, levels of high-density lipoprotein cholesterol (HDL-C) typically increase, which is considered beneficial. Additionally, other indicators such as triglycerides and total cholesterol levels may also improve. While an increase in cholesterol is possible, it does not mean that a low-carbohydrate ketogenic diet is inherently unhealthy. If you are concerned about your cholesterol levels, it is advisable to consult a doctor who can conduct a more detailed assessment to understand your overall risk. Furthermore, a low-carbohydrate ketogenic diet has some potential risks and side effects, such as dehydration, constipation, headaches, and fatigue. Therefore, before adopting this diet, it is important to consult a doctor or a professional nutritionist to ensure that your dietary plan is reasonable and does not lead to malnutrition or other health issues. Statins are a class of medications used to lower cholesterol and blood lipid levels, but there are a few considerations to keep in mind when using them: It is essential to strictly follow the doctor's advice regarding the use of statin medications and not to adjust the dosage or discontinue them without medical guidance. When using statin medications, it is important to adhere to a healthy lifestyle, including a low-fat diet and moderate exercise. Statin medications can cause some side effects such as headaches, muscle pain, and diarrhea, so it is important to promptly inform the doctor and discontinue the medication if necessary. Statin medications may interact with other drugs, leading to adverse reactions or reducing their efficacy. When using statins, it is important to inform the doctor about any other medications being taken. Statin medications may increase the risk of liver function abnormalities, so regular liver function tests are necessary. In summary, when using statin medications, it is important to follow the doctor's advice, be aware of the medication's adverse effects and interactions, and undergo regular monitoring of relevant indicators to ensure safe and effective use of the medication. |
| 24 | *What are some common problems encountered with a low-carbohydrate ketogenic diet?* | (1) Lack of patience: Because the body is not accustomed to using ketones as the primary source of cellular energy, flu-like symptoms can often occur. Many people immediately attribute these symptoms to the low-carbohydrate ketogenic diet, but with patience, usually after two or three days, the flu-like symptoms will completely disappear, allowing you to enter a state of nutritional ketosis. (2) Insufficient fat intake: It is important to remember that at the beginning of a low-carbohydrate ketogenic diet, fat should ideally account for around 70% of total calories, which is already a very high amount. It's important to consume additional oil along with meat. This is contrary to previous dietary habits, as many people are often afraid of consuming too much fat. However, now you need to be prepared mentally and overcome the "fat phobia" that has been ingrained in you for many years. (3) Excessive protein intake: When too much protein is consumed, it can be converted into glucose in the body, leading to increased insulin levels. This prevents the body from efficiently switching its energy source from glucose to ketones, preventing you from experiencing the benefits of a low-carbohydrate ketogenic diet quickly. Therefore, it is important to control the amount of protein intake during meals and avoid excessive consumption. You can calculate the recommended protein intake based on approximately 1g of protein per 1kg of body weight. When buying meat, choose fatty cuts instead of lean ones. Choose chicken thigh meat instead of chicken breast meat and ideally consume the skin as well. When eating fish, it is best to consume the fatty parts. Remember, protein should only account for 15% to 25% of total calories. Consuming too much protein will prevent your body from entering ketosis. (4) Electrolyte deficiencies: Lack of sodium, magnesium, and potassium can lead to symptoms such as headaches, nausea, constipation, drowsiness, fatigue, and muscle cramps. Insulin helps the kidneys store sodium, and when starch intake decreases significantly in the diet, insulin secretion also decreases significantly. As a result, the kidneys' ability to store sodium is affected, leading to a significant loss of sodium in the urine. Therefore, it is important to add an adequate amount of salt to soups and dishes. It is recommended to avoid refined salt and instead opt for low-sodium, high-potassium salt, natural rock salt, lake salt, sea salt, or consider additional potassium supplementation. Potassium is important for heart and overall metabolic function, so it is necessary to consume a large amount of deep green vegetables rich in potassium. Additionally, magnesium plays a crucial role as a catalyst in most biochemical reactions in the body, and simply consuming vegetables may not provide sufficient magnesium, so it may be necessary to supplement with nutritional supplements. (5) Consuming too many hidden carbohydrates: Many processed foods such as fish balls, meatballs, sausages, imitation crab meat, and sweet radish often contain substances that are rich in starch and sugar. It is best to avoid these as much as possible. (6) Eating too few vegetables: Vegetables contain a large amount of vitamins and minerals, which are crucial for normal biochemical reactions in the body. The dietary fiber in vegetables has three major functions: promoting intestinal motility, feeding beneficial gut bacteria, and forming feces. For people who usually rely on starch as their main food source, if they don't consume enough vegetables, they can still obtain some vitamins, minerals, and fiber from whole grains and miscellaneous grains, so it seems to have no impact on their health. However, when switching to a low-carbohydrate ketogenic diet and significantly reducing starch intake, if vegetable intake is not increased, these three nutrients will be noticeably deficient, which may cause various discomfort symptoms such as nausea, sweating, muscle cramps, and constipation. (7) Worries about ketoacidosis and experiencing high psychological stress: Ketoacidosis only occurs in individuals with severely insufficient insulin, such as those with type 1 diabetes or end-stage type 2 diabetes. These individuals have a lack of insulin secretion from the pancreas, causing blood sugar to be unable to enter the cells from the bloodstream. As a result, the cells experience a state of starvation due to the lack of glucose as an energy source, and the body spontaneously initiates the breakdown of fat in the liver, resulting in the production of an excessive amount of ketones (up to 15-25 mmol/L). The blood becomes acidic as ketones are acidic, leading to ketoacidosis, which can be life-threatening. However, as long as there is insulin present, blood ketone levels can be controlled to prevent excessive production of ketones. Normal individuals do not experience uncontrolled ketone levels; they only produce physiological ketosis, which is a nutritional ketosis state and does not lead to pathological ketoacidosis. For generally healthy individuals who are concerned that a low-carbohydrate ketogenic diet may cause ketoacidosis, it is similar to sitting at home watching TV and worrying about a plane crashing into the living room screen—a bit of unnecessary worry. | The potential issues that may frequently occur with a low-carbohydrate ketogenic diet include, but are not limited to: Nutritional imbalance: A low-carbohydrate ketogenic diet may result in inadequate intake of certain nutrients, such as fiber and vitamin C. It is important to pay special attention to food combinations and consider nutritional supplements. Dehydration: A low-carbohydrate ketogenic diet can lead to insufficient water intake, which may cause symptoms like dry mouth and dizziness. It is necessary to increase fluid intake appropriately. Adverse reactions: As mentioned earlier, flu-like symptoms, leg cramps, constipation, and bad breath can occur. Medical history or medication use: It is advisable to consult a doctor before adopting a low-carbohydrate ketogenic diet, especially for individuals with medical history or those currently taking medications. Not suitable for everyone: Certain populations, such as pregnant women, breastfeeding women, children, the elderly, and individuals with impaired liver or kidney function, should avoid a low-carbohydrate ketogenic diet. In conclusion, before adopting a low-carbohydrate ketogenic diet, it is important to carefully evaluate one's physical condition and dietary needs. Seeking professional advice and avoiding unnecessary health risks is highly recommended. |
| 25 | *Why can a low-carbohydrate ketogenic diet help alleviate type 2 diabetes?* | The underlying mechanism of type 2 diabetes is progressive insulin resistance, where the body responds by increasing insulin secretion to overcome insulin resistance. Hyperinsulinemia leads to the storage of glucose and free fatty acids in adipose tissue, muscles, and visceral organs, resulting in continuous weight gain. As a result, insulin resistance worsens, relying on even higher circulating insulin levels. Over time, chronic hyperinsulinemia can lead to beta-cell failure and insulin secretion decline, despite insulin being a proliferative signaling molecule. Hyperinsulinemia is associated with hypertension, cardiovascular disease, and inflammatory conditions, and is an independent risk factor for cardiovascular calcification. Insulin resistance is a predictive factor for the formation of new atherosclerotic plaques and cardiovascular disease. Even seemingly healthy individuals with elevated insulin levels significantly increase their risk of developing type 2 diabetes. Weight loss can effectively increase insulin sensitivity, thereby reducing hyperinsulinemia. A low-carbohydrate ketogenic diet can independently alleviate hyperinsulinemia, even outside the context of weight loss. In other words, if you have hyperinsulinemia while having a normal body mass index, adopting a low-carbohydrate ketogenic diet can improve insulin resistance. When systemic insulin resistance exceeds the pancreas' ability to produce sufficient insulin, hyperglycemia occurs. A low-carbohydrate ketogenic diet restricts foods containing starch or sugar, minimizing blood glucose fluctuations and subsequent insulin demands. Therefore, a low-carbohydrate ketogenic diet can maintain normal blood glucose levels, reduce insulin requirements, improve insulin resistance, interrupt disease progression, and achieve diabetes control. Adopting a low-carbohydrate ketogenic diet can lower the insulin dosage or reduce the need for hypoglycemic medications in diabetic patients, with a lower risk of hypoglycemia. | The ability of a low-carbohydrate ketogenic diet to alleviate type 2 diabetes may be attributed to the following factors: Reduced insulin secretion: The main issue in type 2 diabetes is insulin resistance, where the body's cells have a diminished response to insulin. A low-carbohydrate ketogenic diet can decrease carbohydrate intake, leading to reduced insulin secretion, lower insulin resistance, and improved cellular response to insulin. Increased insulin sensitivity: A low-carbohydrate ketogenic diet can enhance the insulin sensitivity of body cells. This means that cells can utilize insulin more effectively, resulting in lower blood glucose levels and relief from diabetes symptoms. Enhanced glucose metabolism: A low-carbohydrate ketogenic diet promotes the utilization of fatty acids and ketones as an energy source, reducing dependence on glucose while improving the body's ability to utilize glucose. Weight loss: A low-carbohydrate ketogenic diet can aid in weight reduction, which is crucial for individuals with type 2 diabetes. By reducing weight, insulin resistance can be diminished, leading to an improvement in diabetes symptoms. It's important to note that while a low-carbohydrate ketogenic diet can have beneficial effects for individuals with type 2 diabetes, its suitability varies for each person and should be undertaken under the guidance of a medical professional. Prior to adopting a low-carbohydrate ketogenic diet, it is recommended to consult with a doctor and undergo a comprehensive health assessment. |
| 26 | *Does a low-carbohydrate ketogenic diet harm the kidneys?* | It is highly unlikely. However, there is still a common misconception that a low-carbohydrate diet necessarily involves very high protein intake, which could potentially burden the kidneys. This is an incorrect understanding. Firstly, a well-designed low-carbohydrate diet is high in fat, not high in protein. Like in most diets, the consumption of meat should be moderate. Consuming excessive protein is not beneficial because the excess protein can be converted into glucose. Since the recommended low-carbohydrate ketogenic diet does not have a high protein content, there is no need to fear these issues. Secondly, individuals with normal kidney function can handle excessive protein without any harm to the kidneys. Even if someone chooses to consume an excess of protein, it only becomes a problem in cases where the kidneys are already severely damaged. In principle, if you have severe kidney disease and have been advised to limit protein intake, it is important to follow the medical advice. However, even in such cases, it is still possible to successfully follow a low-carbohydrate ketogenic diet that primarily consists of high-fat foods. In summary, there is no reason to worry about excessive protein intake for individuals without kidney disease. Moreover, the most important point is that no one needs to consume excessive protein in a low-carbohydrate ketogenic diet. The diet can be beneficial for the kidneys as it helps protect them from the most common damage caused by high blood sugar levels. Specifically for diabetes patients, a low-carbohydrate ketogenic diet can save their kidneys by aiding in blood sugar control. | The impact of a low-carbohydrate ketogenic diet on the kidneys has been a subject of debate. Some studies suggest that it may cause harm to the kidneys, while others indicate that, under appropriate conditions, a low-carbohydrate ketogenic diet does not have a negative impact on the kidneys. Some studies suggest that a low-carbohydrate ketogenic diet may increase the burden on the kidneys. This is because people on this diet tend to consume higher amounts of protein, and excessive protein intake can potentially strain the kidneys. Additionally, a low-carbohydrate ketogenic diet may lead to dehydration and electrolyte imbalances, which can also burden the kidneys. However, there are also studies indicating that a low-carbohydrate ketogenic diet does not harm the kidneys under appropriate conditions. For example, some research has shown that it can improve kidney function in individuals with kidney disease. In conclusion, if you have kidney disease or other kidney issues, it is advisable to consult with a doctor before adopting a low-carbohydrate ketogenic diet. For the general population, a low-carbohydrate ketogenic diet may not harm the kidneys, but it is important to ensure adequate hydration and proper electrolyte intake to avoid potential side effects. |
| 27 | *Why does blood sugar rise in the morning during diabetes treatment?* | This situation is caused by two reasons: (1) Somogyi effect: Due to excessive insulin dosage, noticeable or unnoticed hypoglycemia often occurs around 2-3 am after midnight. This stimulates the reflex secretion of glucose-raising hormones in the body, such as growth hormone, cortisol, and glucagon, leading to an increase in blood sugar in the morning. (2) Dawn phenomenon: Due to increased physiological release of blood sugar-raising hormones after midnight and in the early morning, there is an increased release of liver glycogen to maintain normal fasting blood sugar levels. Since individuals with diabetes have insufficient or completely lacking insulin secretion, the elevated blood sugar cannot be lowered, resulting in elevated fasting blood sugar levels known as the "dawn phenomenon." The differentiation between the two mainly relies on checking blood sugar levels between 2-3 am after midnight. If the blood sugar is <3.3mmol/L, it is diagnosed as the Somogyi effect, and low blood sugar treatment should be followed by appropriately increasing carbohydrate intake or reducing intermediate-acting insulin injections before dinner. If the blood sugar is >3.9mmol/L, it is diagnosed as the dawn phenomenon, and carbohydrate intake should be reduced or intermediate-acting insulin should be increased before dinner. | In individuals with diabetes, the phenomenon of elevated blood sugar in the morning is known as the "dawn phenomenon" or "morning hyperglycemia." This occurrence can be attributed to several factors. One major factor is the release of glycogen by the liver. During sleep, the body requires energy to maintain normal physiological functions, but food intake is reduced, and the body needs to obtain energy from alternative sources. The liver can break down stored glycogen into glucose and release it into the bloodstream to provide energy. However, in individuals with diabetes, due to inadequate insulin secretion or increased insulin resistance in cells, the glucose in the blood cannot be effectively utilized, leading to elevated blood sugar levels. Another factor is the influence of hormone secretion. In the morning, the body secretes hormones such as adrenaline and glucagon, which can cause an increase in blood sugar levels. Additionally, the long-term use of certain medications, such as sedatives and beta-blockers, may also contribute to the occurrence of morning hyperglycemia. To prevent morning hyperglycemia, individuals with diabetes can take the following measures: adjusting medication dosages, selecting the appropriate medications, consuming a small meal before bedtime, increasing physical exercise, and so on. However, it is recommended that patients consult with their doctors before making any of these changes to ensure that the measures taken are safe and effective. |
| 28 | *How do the ketogenic diet and the Mediterranean diet differ in their effects on the gut microbiota? (Additional question)* | | Both the ketogenic diet and the Mediterranean diet can potentially have an impact on the gut microbiota, but these effects may differ. The ketogenic diet is a high-fat, low-carbohydrate dietary approach that primarily aims to induce a state of ketosis by restricting carbohydrate intake. Research suggests that the ketogenic diet may lead to changes in the gut microbiota, including a reduction in certain types of gut bacteria such as Bacteroides and an increase in other types of bacteria such as Akkermansia and Lactobacillus. These changes in the gut microbiota could potentially influence gut health and immune function, but further research is needed to understand the specific effects. In contrast, the Mediterranean diet is a low-fat, high-fiber dietary pattern that emphasizes the consumption of vegetables, fruits, whole grains, legumes, nuts, and olive oil. Studies indicate that the Mediterranean diet may increase the abundance of beneficial gut bacteria such as Bifidobacterium and Lactobacillus while decreasing the levels of certain unfavorable bacteria such as Clostridium perfringens. These changes in the gut microbiota may promote gut health, reduce the risk of chronic diseases, and enhance immune function. In summary, both the ketogenic diet and the Mediterranean diet may have an impact on the gut microbiota, but the specific effects could differ. Since the gut microbiota is closely associated with health, it is important to choose a dietary pattern that suits an individual's needs. |
